# Supplementary figures and images for: Are lipids always depleted? Comparison of hydrogen, carbon, and nitrogen isotopic values in the muscle and lipid of larval lampreys
Source: PLoS One. 2024 Jan 11;19(1):e0286535. doi: 10.1371/journal.pone.0286535 (PMC10783746; doi:10.1371/journal.pone.0286535)

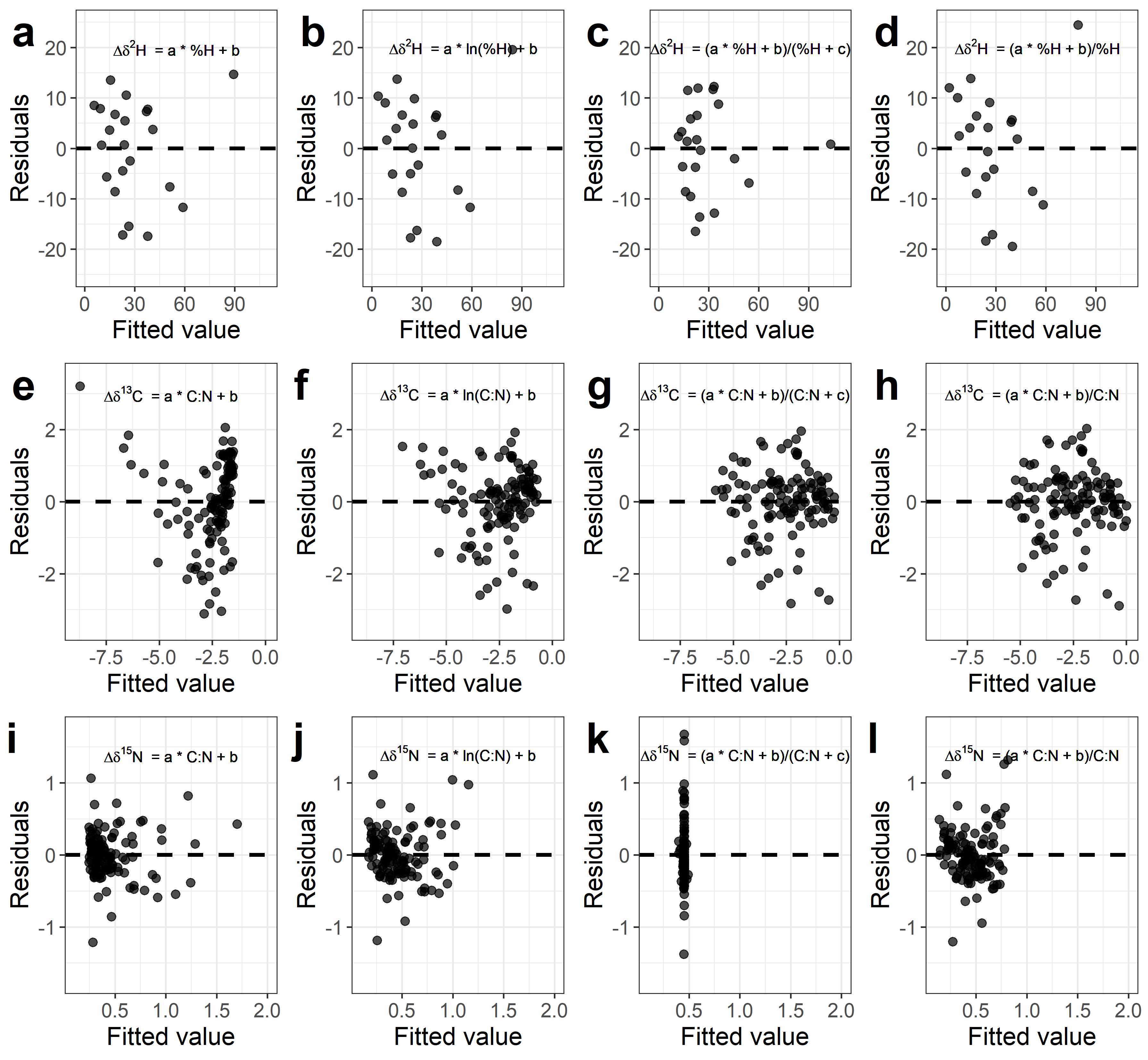

Supplement: S1 Fig — (TIFF) [file pone.0286535.s001.tiff]

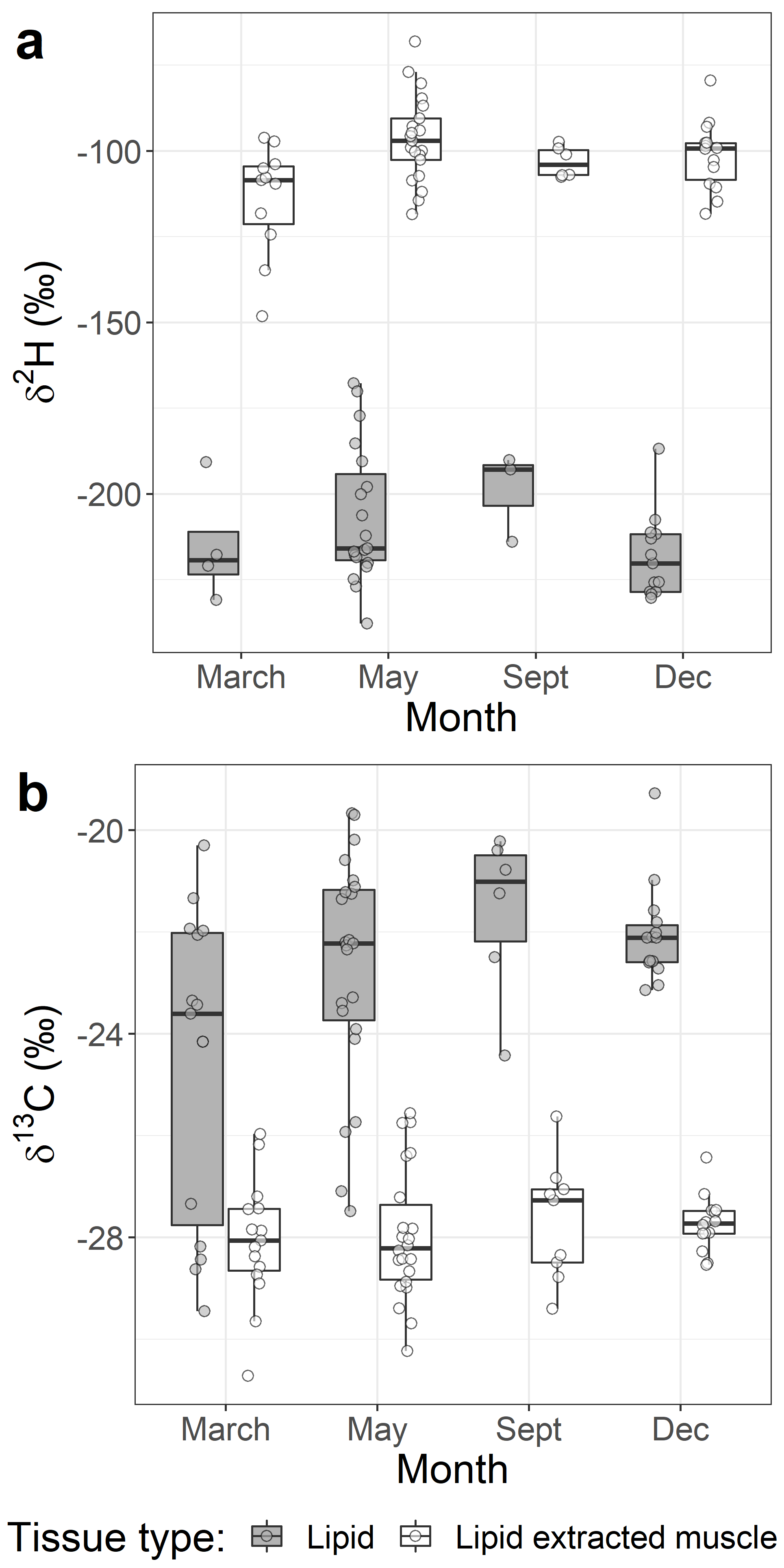

Supplement: S2 Fig — The a) δ2H, and b) δ13C for lipid (grey) and lipid extracted muscle (white) samples for lamprey collected in Maryland. Boxplot midlines are the median, the boxes enclose the 1st-3rd quartiles (i.e., 50% of the data points), and the whiskers are 1.5 times this interquartile range. Measured values that boxplots are drawn from are represented as points and are jittered to make them more easily distinguishable. (TIFF) [file pone.0286535.s002.tiff]
