# Supplementary material for: Are lipids always depleted? Comparison of hydrogen, carbon, and nitrogen isotopic values in the muscle and lipid of larval lampreys
Source: PLoS One. 2024 Jan 11;19(1):e0286535. doi: 10.1371/journal.pone.0286535 (PMC10783746; doi:10.1371/journal.pone.0286535)
Supplement: S1 File — (DOCX) [file pone.0286535.s003.docx]

Supplementary Tables for:

**Are lipids always depleted? Comparison of hydrogen, carbon, and nitrogen isotopic values in the muscle and lipid of larval lampreys**

Thomas M Evans, Shale Beharie

**Table S1.** Site location for lampreys reported in prior studies or collected outside of Maryland.

| Site | Date of Collection | River Name | Latitude | Longitude |
| --- | --- | --- | --- | --- |
| PR01 | 5/19/2010 | Pigeon River | 45.27182 | -84.45981 |
| JR01 | 6/10/2010 | Jordan River | 45.01463 | -85.02997 |
| JR02 | 6/10/2010 | Jordan River | 45.10208 | -85.09792 |
| CF01 | 7/8/2010 | Clearfork River | 40.59265 | -82.42055 |
| CF02 | 7/8/2010 | Clearfork River | 40.57258 | -82.40868 |
| MR01 | 7/1/2010 | Mad River | 40.25435 | -83.74767 |
| MR02 | 7/1/2010 | Mad River | 40.33646 | -83.67270 |
| PR01F | 10/2/2010 | Pigeon River | 45.27182 | -84.45981 |
| JR01F | 10/3/2010 | Jordan River | 45.01463 | -85.02997 |
| JR02F | 10/3/2010 | Jordan River | 45.10208 | -85.09792 |
| CF01F | 11/7/2010 | Clearfork River | 40.59265 | -82.42055 |
| CF02F | 11/7/2010 | Clearfork River | 40.57258 | -82.40868 |
| MR01F | 11/6/2010 | Mad River | 40.25435 | -83.74767 |
| MR02F | 11/6/2010 | Mad River | 40.33646 | -83.67270 |
| TB01 | 8/7/2017 | Trout Brook | 44.65528 | -74.59477 |
| TB02 | 8/7/2017 | Trout Brook | 44.69692 | -74.80573 |
| GR03 | 8/8/2017 | Cryder Creek | 42.04924 | -77.74846 |
| GR04 | 8/9/2017 | Genesee River | 42.15240 | -77.98070 |
| GR06 | 8/9/2017 | Genesee River | 42.01549 | -77.89455 |
| GR07 | 8/10/2017 | Dyke Creek | 42.15037 | -77.81740 |
| GR08 | 8/10/2017 | Dyke Creek | 42.14969 | -77.85477 |
| GR09 | 8/11/2017 | Genesee River | 42.20651 | -78.02282 |
| GR10 | 8/11/2017 | Genesee River | 42.07578 | -77.92450 |
| YR99 | 7/18/2017 | Yakima Hatchery | 46.21427 | -119.75978 |

**Table S2.** For samples reported in prior studies or collected outside of Maryland the species, length, and weight for each individual.

| Individual | Species | Length (mm) | Wet weight (g) |
| --- | --- | --- | --- |
| CF01.2010.1.L.001 | LBL | 84 | 1.63 |
| CF01.2010.1.L.002 | LBL | 100 | 2.34 |
| CF01.2010.1.L.003 | LBL | 103 | 2.50 |
| CF01.2010.1.L.004 | LBL | 116 | 4.08 |
| CF01.2010.1.L.005 | LBL | 121 | 4.31 |
| CF01.2010.1.L.006 | LBL | 48 | 0.66 |
| CF01.2010.1.L.007 | LBL | 100 | 2.47 |
| CF01.2010.1.L.008 | LBL | 90 | 1.85 |
| CF01F.2010.1.L.001 | LBL | 111 | 2.54 |
| CF01F.2010.1.L.002 | LBL | 132 | 5.29 |
| CF01F.2010.1.L.003 | LBL | 93 | 1.81 |
| CF01F.2010.1.L.004 | LBL | 71 | 0.91 |
| CF01F.2010.1.L.005 | LBL | 79 | 1.19 |
| CF01F.2010.1.L.006 | LBL | 121 | 4.34 |
| CF02.2010.1.L.001 | LBL | 119 | 3.69 |
| CF02.2010.1.L.002 | LBL | 82 | 1.56 |
| CF02.2010.1.L.003 | LBL | 82 | 1.31 |
| CF02.2010.1.L.004 | LBL | 146 | 7.14 |
| CF02.2010.1.L.005 | LBL | 30 | 0.08 |
| CF02.2010.1.L.006 | LBL |  |  |
| CF02.2010.1.L.007 | LBL |  |  |
| CF02.2010.1.L.008 | LBL |  |  |
| CF02F.2010.1.L.001 | LBL | 30 | 0.09 |
| CF02F.2010.1.L.002 | LBL | 35 | 0.13 |
| CF02F.2010.1.L.003 | LBL | 35 | 0.11 |
| CF02F.2010.1.L.004 | LBL | 92 | 2.18 |
| CF02F.2010.1.L.005 | LBL | 103 | 3.41 |
| CF02F.2010.1.L.006 | LBL | 94 | 2.42 |
| CF02F.2010.1.L.007 | LBL | 33 | 0.10 |
| CF02F.2010.1.L.008 | LBL | 108 | 4.08 |
| CF02F.2010.1.L.009 | LBL | 32 | 0.08 |
| CF02F.2010.1.L.010 | LBL | 52 | 0.43 |
| CF02F.2010.1.L.011 | LBL | 34 | 0.13 |
| JR01.2010.1.L.006 | SL | 33 | 0.20 |
| JR01.2010.1.L.007 | SL | 49 | 0.36 |
| JR01.2010.1.L.008 | SL | 34 | 0.16 |
| JR01.2010.1.L.009 | SL | 40 | 0.27 |
| JR01.2010.1.L.010 | SL | 45 | 0.35 |
| JR01.2010.1.L.011 | SL | 56 | 0.59 |
| JR01.2010.1.L.012 | SL | 43 | 0.31 |
| JR01.2010.1.L.013 | SL | 34 | 0.14 |
| JR01.2010.1.L.014 | SL | 55 | 0.52 |
| JR01F.2010.1.L.001 | SL | 74 | 0.99 |
| JR01F.2010.1.L.002 | SL | 69 | 0.81 |
| JR01F.2010.1.L.003 | SL | 39 | 0.18 |
| JR01F.2010.1.L.004 | SL | 40 | 0.18 |
| JR01F.2010.1.L.005 | SL | 71 | 0.72 |
| JR01F.2010.1.L.006 | SL | 60 | 0.51 |
| JR01F.2010.1.L.007 | SL | 43 | 0.26 |
| JR01F.2010.1.L.008 | SL | 17 | 0.02 |
| JR01F.2010.1.L.009 | SL | 19 | 0.02 |
| JR01F.2010.1.L.010 | SL | 43 | 0.21 |
| JR01F.2010.1.L.011 | SL | 46 | 0.30 |
| JR01F.2010.1.L.012 | SL | 33 | 0.14 |
| JR01F.2010.1.L.013 | SL | 38 | 0.14 |
| JR01F.2010.1.L.014 | SL | 20 | 0.02 |
| JR01F.2010.1.L.015 | SL | 17 | 0.01 |
| JR01F.2010.1.L.016 | SL | 47 | 0.32 |
| JR01F.2010.1.L.017 | SL | 42 | 0.22 |
| JR01F.2010.1.L.018 | SL | 54 | 0.39 |
| JR01F.2010.1.L.019 | SL | 19 | 0.02 |
| JR01F.2010.1.L.020 | SL | 14 | 0.01 |
| JR02.2010.1.L.017 | SL | 50 | 0.36 |
| JR02.2010.1.L.018 | SL | 25 | 0.07 |
| JR02.2010.1.L.019 | SL | 63 | 0.73 |
| JR02.2010.1.L.020 | SL | 56 | 0.49 |
| JR02F.2010.1.L.001 | SL | 80 | 1.17 |
| JR02F.2010.1.L.002 | SL | 18 | 0.02 |
| JR02F.2010.1.L.003 | SL | 19 | 0.03 |
| JR02F.2010.1.L.004 | SL | 18 | 0.02 |
| JR02F.2010.1.L.005 | SL | 17 | 0.02 |
| JR02F.2010.1.L.006 | SL | 20 | 0.02 |
| JR02F.2010.1.L.007 | SL | 14 | 0.01 |
| JR02F.2010.1.L.008 | SL | 53 | 0.36 |
| JR02F.2010.1.L.009 | SL | 20 | 0.02 |
| JR02F.2010.1.L.010 | SL | 21 | 0.03 |
| PR01.2010.1.L.001 | SL | 70 | 1.02 |
| PR01.2010.1.L.002 | SL | 42 | 0.34 |
| PR01.2010.1.L.003 | SL | 63 | 0.68 |
| PR01.2010.1.L.004 | SL | 52 | 0.52 |
| PR01.2010.1.L.005 | SL | 42 | 0.33 |
| PR01F.2010.1.L.001 | SL | 30 | 0.05 |
| PR01F.2010.1.L.002 | SL | 51 | 0.27 |
| PR01F.2010.1.L.003 | SL | 87 | 1.49 |
| PR01F.2010.1.L.004 | SL | 94 | 1.79 |
| PR01F.2010.1.L.005 | SL | 31 | 0.08 |
| PR01F.2010.1.L.006 | SL | 27 | 0.06 |
| PR01F.2010.1.L.007 | SL | 29 | 0.05 |
| PR01F.2010.1.L.008 | SL | 31 | 0.08 |
| PR01F.2010.1.L.009 | SL | 107 | 2.36 |
| PR01F.2010.1.L.010 | SL | 55 | 0.43 |
| PR01F.2010.1.L.011 | SL | 31 | 0.06 |
| PR01F.2010.1.L.012 | SL | 86 | 1.44 |
| PR01F.2010.1.L.013 | SL | 56 | 0.70 |
| PR01F.2010.1.L.014 | SL | 61 | 0.64 |
| MR01.2010.1.L.001 | ABL | 142 | 4.80 |
| MR01.2010.1.L.002 | ABL | 131 | 4.40 |
| MR01.2010.1.L.003 | ABL | 163 | 7.62 |
| MR01.2010.1.L.004 | ABL | 96 | 1.63 |
| MR01.2010.1.L.005 | ABL | 138 | 4.60 |
| MR01.2010.1.L.006 | ABL | 156 | 6.08 |
| MR01.2010.1.L.007 | ABL | 165 | 7.05 |
| MR01.2010.1.L.008 | ABL | 160 | 7.32 |
| MR01.2010.1.L.009 | ABL | 147 | 5.81 |
| MR01.2010.1.L.010 | ABL | 166 | 7.51 |
| MR01.2010.1.L.011 | ABL | 152 | 6.20 |
| MR01.2010.1.L.012 | ABL | 132 | 3.96 |
| MR01F.2010.1.L.001 | ABL | 95 | 1.23 |
| MR01F.2010.1.L.002 | ABL | 123 | 2.31 |
| MR01F.2010.1.L.003 | ABL | 188 | 9.89 |
| MR01F.2010.1.L.004 | ABL | 157 | 5.81 |
| MR01F.2010.1.L.005 | ABL | 145 | 4.58 |
| MR01F.2010.1.L.006 | ABL | 164 | 6.24 |
| MR01F.2010.1.L.007 | ABL | 158 | 6.63 |
| MR01F.2010.1.L.008 | ABL | 155 | 6.55 |
| MR02.2010.1.L.001 | ABL | 136 | 4.26 |
| MR02.2010.1.L.002 | ABL | 167 | 7.54 |
| MR02.2010.1.L.003 | ABL | 163 | 9.90 |
| MR02.2010.1.L.004 | ABL | 162 | 7.92 |
| MR02.2010.1.L.005 | ABL | 189 | 11.84 |
| MR02.2010.1.L.006 | ABL | 86 | 1.28 |
| MR02.2010.1.L.007 | ABL | 179 | 8.94 |
| MR02.2010.1.L.008 | ABL | 166 | 7.54 |
| MR02.2010.1.L.009 | ABL | 161 | 7.33 |
| MR02.2010.1.L.010 | ABL | 126 | 3.23 |
| MR02.2010.1.L.011 | ABL | 163 | 8.23 |
| MR02.2010.1.L.012 | ABL | 92 | 1.54 |
| MR02.2010.1.L.013 | ABL | 109 | 2.09 |
| MR02F.2010.1.L.001 | ABL | 165 | 6.88 |
| MR02F.2010.1.L.002 | ABL | 133 | 4.13 |
| MR02F.2010.1.L.003 | ABL | 80 | 1.00 |
| MR02F.2010.1.L.004 | ABL | 167 | 7.85 |
| MR02F.2010.1.L.005 | ABL | 100 | 1.80 |
| MR02F.2010.1.L.006 | ABL | 127 | 3.74 |
| MR02F.2010.1.L.007 | ABL | 142 | 4.46 |
| MR02F.2010.1.L.008 | ABL | 160 | 6.75 |
| TB01.2017.1.L.001 | ABL | 155 |  |
| TB01.2017.1.L.002 | ABL | 131 | 3.12 |
| TB01.2017.1.L.003 | ABL | 85 | 1.22 |
| TB01.2017.1.L.004 | ABL | 26 |  |
| TB01.2017.1.L.007 | ABL | 30 |  |
| TB02.2017.1.L.001 | ABL | 111 | 1.92 |
| TB02.2017.1.L.002 | ABL | 149 | 4.13 |
| TB02.2017.1.L.003 | ABL | 160 | 5.88 |
| TB02.2017.1.L.004 | ABL | 147 | 4.26 |
| TB02.2017.1.L.005 | ABL | 149 | 4.06 |
| TB02.2017.1.L.006 | ABL | 138 | 3.39 |
| TB02.2017.1.L.007 | ABL | 123 | 2.70 |
| TB02.2017.1.L.008 | ABL | 132 | 2.82 |
| TB02.2017.1.L.010 | ABL | 112 | 2.70 |
| TB02.2017.1.L.012 | ABL | 129 | 2.88 |
| TB02.2017.1.L.015 | ABL | 64 | 0.54 |
| TB02.2017.1.L.016 | ABL | 102 |  |
| GR03.2017.1.L.001 | ABL | 49 |  |
| GR03.2017.1.L.002 | ABL | 39 |  |
| GR03.2017.1.L.003 | ABL | 43 |  |
| GR03.2017.1.L.004 | ABL | 41 |  |
| GR03.2017.1.L.005 | ABL | 38 |  |
| GR03.2017.1.L.012 | ABL | 95 | 1.27 |
| GR03.2017.1.L.013 | ABL | 101 | 1.50 |
| GR03.2017.1.L.014 | ABL | 116 | 1.73 |
| GR03.2017.1.L.015 | ABL | 114 |  |
| GR03.2017.1.L.016 | ABL | 174 | 5.56 |
| GR03.2017.1.L.017 | ABL | 138 | 3.50 |
| GR03.2017.1.L.018 | ABL | 128 | 2.80 |
| GR03.2017.1.L.019 | ABL | 109 | 1.66 |
| GR03.2017.1.L.020 | ABL | 102 | 1.47 |
| GR03.2017.1.L.032 | ABL | 89 | 1.01 |
| GR03.2017.1.L.033 | ABL | 92 | 1.06 |
| GR03.2017.1.L.039 | ABL | 107 | 1.45 |
| GR03.2017.1.L.041 | ABL | 143 | 3.26 |
| GR03.2017.1.L.043 | ABL | 98 | 1.40 |
| GR03.2017.1.L.044 | ABL | 182 | 6.92 |
| GR03.2017.1.L.046 | ABL | 90 | 1.04 |
| GR03.2017.1.L.052 | ABL | 117 | 2.28 |
| GR03.2017.1.L.053 | ABL | 129 | 2.81 |
| GR04.2017.1.L.001 | ABL | 34 |  |
| GR04.2017.1.L.002 | ABL | 27 |  |
| GR04.2017.1.L.003 | ABL | 30 |  |
| GR04.2017.1.L.004 | ABL | 32 |  |
| GR04.2017.1.L.005 | ABL | 31 |  |
| GR06.2017.1.L.001 | ABL | 33 |  |
| GR06.2017.1.L.003 | ABL | 38 |  |
| GR06.2017.1.L.004 | ABL | 32 |  |
| GR06.2017.1.L.005 | ABL | 28 |  |
| GR06.2017.1.L.006 | ABL | 32 |  |
| GR06.2017.1.L.008 | ABL | 101 | 1.23 |
| GR06.2017.1.L.009 | ABL | 136 | 2.94 |
| GR06.2017.1.L.010 | ABL | 165 | 4.49 |
| GR06.2017.1.L.011 | ABL | 219 | 10.76 |
| GR06.2017.1.L.012 | ABL | 106 | 1.83 |
| GR06.2017.1.L.013 | ABL | 121 | 2.51 |
| GR06.2017.1.L.020 | ABL | 160 | 4.20 |
| GR06.2017.1.L.021 | ABL | 212 | 11.89 |
| GR07.2017.1.L.001 | ABL | 22 |  |
| GR07.2017.1.L.002 | ABL | 23 |  |
| GR07.2017.1.L.003 | ABL | 29 |  |
| GR07.2017.1.L.004 | ABL | 25 |  |
| GR07.2017.1.L.005 | ABL | 23 |  |
| GR07.2017.1.L.018 | ABL | 65 | 0.42 |
| GR07.2017.1.L.020 | ABL | 99 | 1.46 |
| GR07.2017.1.L.022 | ABL | 100 | 1.45 |
| GR07.2017.1.L.024 | ABL | 131 |  |
| GR07.2017.1.L.026 | ABL | 108 | 1.63 |
| GR07.2017.1.L.028 | ABL | 120 | 2.29 |
| GR07.2017.1.L.030 | ABL | 91 | 1.08 |
| GR07.2017.1.L.032 | ABL | 84 | 0.86 |
| GR07.2017.1.L.034 | ABL | 81 | 0.87 |
| GR07.2017.1.L.036 | ABL | 91 | 0.79 |
| GR07.2017.1.L.049 | ABL | 65 | 0.48 |
| GR08.2017.1.L.001 | ABL | 172 | 6.22 |
| GR08.2017.1.L.002 | ABL | 97 | 1.25 |
| GR08.2017.1.L.003 | ABL | 111 | 1.68 |
| GR08.2017.1.L.004 | ABL | 86 | 0.96 |
| GR08.2017.1.L.005 | ABL | 108 | 1.15 |
| GR08.2017.1.L.006 | ABL | 101 | 1.46 |
| GR08.2017.1.L.007 | ABL | 90 | 1.07 |
| GR08.2017.1.L.009 | ABL | 89 | 1.22 |
| GR08.2017.1.L.010 | ABL | 74 | 0.70 |
| GR08.2017.1.L.011 | ABL | 78 |  |
| GR08.2017.1.L.012 | ABL | 37 |  |
| GR08.2017.1.L.013 | ABL | 30 |  |
| GR08.2017.1.L.014 | ABL | 32 |  |
| GR08.2017.1.L.015 | ABL | 25 |  |
| GR08.2017.1.L.018 | ABL | 135 | 2.83 |
| GR08.2017.1.L.021 | ABL | 153 | 4.10 |
| GR08.2017.1.L.022 | ABL | 118 | 2.23 |
| GR08.2017.1.L.023 | ABL | 79 | 0.70 |
| GR08.2017.1.L.034 | ABL | 56 | 0.40 |
| GR08.2017.1.L.035 | ABL | 68 | 0.47 |
| GR09.2017.1.L.001 | ABL | 24 |  |
| GR09.2017.1.L.002 | ABL | 28 |  |
| GR09.2017.1.L.003 | ABL | 30 |  |
| GR09.2017.1.L.004 | ABL | 38 |  |
| GR09.2017.1.L.005 | ABL | 29 |  |
| GR09.2017.1.L.011 | ABL | 86 | 0.88 |
| GR09.2017.1.L.014 | ABL | 106 | 1.19 |
| GR09.2017.1.L.015 | ABL | 108 | 1.77 |
| GR09.2017.1.L.016 | ABL | 112 | 1.54 |
| GR09.2017.1.L.017 | ABL | 123 | 2.23 |
| GR09.2017.1.L.018 | ABL | 198 | 9.29 |
| GR09.2017.1.L.019 | ABL | 175 | 6.57 |
| GR09.2017.1.L.022 | ABL | 148 | 3.71 |
| GR09.2017.1.L.023 | ABL | 117 | 1.61 |
| GR09.2017.1.L.024 | ABL | 129 | 2.49 |
| GR09.2017.1.L.025 | ABL | 121 | 1.79 |
| GR09.2017.1.L.026 | ABL | 91 | 1.13 |
| GR09.2017.1.L.027 | ABL | 131 | 3.67 |
| GR09.2017.1.L.029 | ABL | 220 | 12.36 |
| GR10.2017.1.L.001 | ABL | 125 | 2.57 |
| GR10.2017.1.L.002 | ABL | 127 | 2.61 |
| GR10.2017.1.L.003 | ABL | 183 | 7.17 |
| GR10.2017.1.L.004 | ABL | 81 | 0.84 |
| GR10.2017.1.L.005 | ABL | 85 | 0.94 |
| GR10.2017.1.L.006 | ABL | 151 | 4.48 |
| GR10.2017.1.L.007 | ABL | 132 | 2.90 |
| GR10.2017.1.L.008 | ABL | 161 | 4.84 |
| GR10.2017.1.L.009 | ABL | 130 | 3.43 |
| GR10.2017.1.L.010 | ABL | 121 | 2.20 |
| GR10.2017.1.L.011 | ABL | 135 | 2.98 |
| GR10.2017.1.L.012 | ABL | 74 | 0.66 |
| GR10.2017.1.L.013 | ABL | 189 | 8.46 |
| GR10.2017.1.L.014 | ABL | 85 | 1.03 |
| GR10.2017.1.L.015 | ABL | 138 | 3.90 |
| GR10.2017.1.L.016 | ABL | 146 | 4.60 |
| GR10.2017.1.L.017 | ABL | 140 | 3.58 |
| YR99.2017.1.L.001 | PL | 123 | 2.41 |
| YR99.2017.1.L.002 | PL | 132 | 2.92 |
| YR99.2017.1.L.003 | PL | 123 | 2.64 |
| YR99.2017.1.L.004 | PL | 117 | 2.31 |
| YR99.2017.1.L.005 | PL | 115 | 2.17 |

Note: ABL is American brook lamprey (*Lethenteron appendix*), LBL is least brook lamprey (*Lampetra aepyptera*), PL is Pacific lamprey (*Entosphenus tridentatus*), SL is sea lamprey (*Petromyzon marinus*).

**Table S3.** The samples reported in prior studies or collected outside of Maryland submitted for isotope analysis, whether they were lipid extracted, or duplicated, and measurements of isotope ratios and percentage of each atom in the sample.

| Individual | Tissue | Lipid extracted? | Duplicate | % N | δ^15^N (‰) | % C | δ^13^C (‰) | % H | δ^2^H(‰) | |  |
| --- | --- | --- | --- | --- | --- | --- | --- | --- | --- | --- | --- |
| CF01.2010.1.L.001 | MUSCLE | NO | NO | 10.72 | 4.69 | 54.01 | -25.34 |  |  | |  |
| CF01.2010.1.L.002 | MUSCLE | NO | NO | 7.80 | 4.54 | 58.80 | -23.57 |  |  | |  |
| CF01.2010.1.L.003 | MUSCLE | NO | NO | 9.86 | 3.24 | 54.54 | -23.95 |  |  | |  |
| CF01.2010.1.L.004 | MUSCLE | NO | NO | 7.39 | 6.55 | 61.06 | -24.41 |  |  | |  |
| CF01.2010.1.L.004 | MUSCLE | NO | YES | 7.00 | 6.37 | 61.83 | -24.19 |  |  | |  |
| CF01.2010.1.L.005 | MUSCLE | NO | NO | 7.33 | 6.68 | 60.81 | -24.76 |  |  | |  |
| CF01.2010.1.L.006 | MUSCLE | NO | NO | 10.65 | 6.04 | 52.90 | -25.64 |  |  | |  |
| CF01.2010.1.L.007 | MUSCLE | NO | NO | 11.25 | 3.66 | 53.55 | -24.34 |  |  | |  |
| CF01.2010.1.L.008 | MUSCLE | NO | NO | 6.77 | 4.77 | 59.73 | -23.65 |  |  | |  |
| CF01F.2010.1.L.001 | MUSCLE | NO | NO | 9.72 | 3.75 | 55.75 | -24.67 | 7.50 | -174.80 | |  |
| CF01F.2010.1.L.002 | MUSCLE | NO | NO | 8.26 | 7.31 | 59.31 | -23.09 | 7.90 | -206.70 | |  |
| CF01F.2010.1.L.003 | MUSCLE | NO | NO | 12.87 | 4.95 | 48.61 | -28.42 | 6.30 | -137.40 | |  |
| CF01F.2010.1.L.004 | MUSCLE | NO | YES | 14.00 | 7.67 | 48.95 | -29.35 | 6.10 | -141.50 | |  |
| CF01F.2010.1.L.004 | MUSCLE | NO | YES | 12.73 | 7.37 | 49.27 | -26.54 |  |  | |  |
| CF01F.2010.1.L.004 | MUSCLE | NO | NO | 12.61 | 7.40 | 47.93 | -26.58 |  |  | |  |
| CF01F.2010.1.L.005 | MUSCLE | NO | NO | 13.51 | 6.15 | 49.41 | -26.77 | 6.20 | -133.40 | |  |
| CF01F.2010.1.L.006 | MUSCLE | NO | NO | 8.72 | 4.77 | 56.84 | -24.23 | 7.90 | -193.70 | |  |
| CF02.2010.1.L.001 | MUSCLE | NO | NO | 12.05 | 7.40 | 50.65 | -22.88 |  |  | |  |
| CF02.2010.1.L.002 | MUSCLE | NO | NO | 11.69 | 7.35 | 52.92 | -24.16 |  |  | |  |
| CF02.2010.1.L.003 | MUSCLE | NO | NO | 13.21 | 7.27 | 49.73 | -24.04 |  |  | |  |
| CF02.2010.1.L.004 | MUSCLE | NO | NO | 8.57 | 6.87 | 58.21 | -22.99 |  |  | |  |
| CF02.2010.1.L.004 | MUSCLE | NO | YES | 8.75 | 6.72 | 57.78 | -23.21 |  |  | |  |
| CF02.2010.1.L.005 | MUSCLE | NO | NO | 10.52 | 5.11 | 51.73 | -24.12 |  |  | |  |
| CF02.2010.1.L.006 | MUSCLE | NO | NO | 13.54 | 6.86 | 48.60 | -24.86 |  |  | |  |
| CF02.2010.1.L.007 | MUSCLE | NO | NO | 14.01 | 7.25 | 49.48 | -25.19 |  |  | |  |
| CF02.2010.1.L.008 | MUSCLE | NO | NO | 13.22 | 5.93 | 48.10 | -26.68 | 3.64 |  |  | |
| CF02F.2010.1.L.001 | MUSCLE | NO | NO | 12.97 | 7.79 | 47.07 | -27.79 | 3.63 |  |  | |
| CF02F.2010.1.L.002 | MUSCLE | NO | NO | 13.61 | 8.48 | 48.26 | -27.93 | 3.55 |  |  | |
| CF02F.2010.1.L.003 | MUSCLE | NO | NO | 13.85 | 6.91 | 48.54 | -28.71 | 3.51 |  |  | |
| CF02F.2010.1.L.004 | MUSCLE | NO | NO | 13.69 | 8.42 | 48.74 | -25.52 | 3.56 |  |  | |
| CF02F.2010.1.L.005 | MUSCLE | NO | NO | 13.69 | 7.68 | 49.15 | -23.96 | 3.59 |  |  | |
| CF02F.2010.1.L.006 | MUSCLE | NO | NO | 13.14 | 7.85 | 49.70 | -24.47 | 3.78 |  |  | |
| CF02F.2010.1.L.007 | MUSCLE | NO | NO | 13.09 | 9.04 | 48.36 | -27.82 | 3.69 |  |  | |
| CF02F.2010.1.L.008 | MUSCLE | NO | NO | 10.97 | 7.00 | 53.97 | -23.69 | 4.92 |  |  | |
| CF02F.2010.1.L.009 | MUSCLE | NO | NO | 13.65 | 6.86 | 48.06 | -28.83 | 3.52 |  |  | |
| CF02F.2010.1.L.010 | MUSCLE | NO | NO | 13.65 | 9.01 | 48.11 | -24.24 | 3.52 |  |  | |
| CF02F.2010.1.L.011 | MUSCLE | NO | NO | 12.42 | 8.23 | 50.56 | -28.86 | 4.07 |  |  | |
| JR01.2010.1.L.006 | MUSCLE | NO | NO | 8.94 | 5.16 | 55.03 | -25.78 | 6.15 |  |  | |
| JR01.2010.1.L.007 | MUSCLE | NO | NO | 9.87 | 3.99 | 55.15 | -26.70 | 5.59 |  |  | |
| JR01.2010.1.L.008 | MUSCLE | NO | NO | 11.34 | 4.07 | 50.68 | -28.42 | 4.47 |  |  | |
| JR01.2010.1.L.009 | MUSCLE | NO | NO | 9.54 | 4.37 | 54.49 | -26.89 | 5.71 |  |  | |
| JR01.2010.1.L.010 | MUSCLE | NO | NO | 8.28 | 3.54 | 57.69 | -26.61 | 6.97 |  |  | |
| JR01.2010.1.L.011 | MUSCLE | NO | NO | 8.25 | 3.41 | 58.85 | -25.33 | 7.13 |  |  | |
| JR01.2010.1.L.011 | MUSCLE | NO | YES | 7.14 | 3.06 | 59.03 | -25.56 | 8.26 |  |  | |
| JR01.2010.1.L.012 | MUSCLE | NO | NO | 10.06 | 4.79 | 52.90 | -27.83 | 5.26 |  |  | |
| JR01.2010.1.L.012 | MUSCLE | NO | YES | 10.10 | 4.69 | 53.17 | -27.81 | 5.27 |  |  | |
| JR01.2010.1.L.013 | MUSCLE | NO | NO | 8.81 | 4.04 | 55.12 | -27.57 | 6.25 |  |  | |
| JR01.2010.1.L.014 | MUSCLE | NO | NO | 7.53 | 3.74 | 59.99 | -24.91 | 7.97 |  |  | |
| JR01F.2010.1.L.001 | MUSCLE | NO | NO | 7.00 | 3.70 | 58.86 | -25.25 | 8.41 | 8.20 | -202.90 | |
| JR01F.2010.1.L.002 | MUSCLE | NO | NO | 6.17 | 3.46 | 57.05 | -24.61 | 9.25 | 8.70 | -220.90 | |
| JR01F.2010.1.L.003 | MUSCLE | NO | NO | 7.74 | 4.28 | 55.28 | -25.57 | 7.14 | 7.80 | -203.60 | |
| JR01F.2010.1.L.003 | MUSCLE | NO | YES |  |  |  |  |  | 7.70 | -203.90 | |
| JR01F.2010.1.L.004 | MUSCLE | NO | NO | 9.92 | 5.44 | 53.32 | -27.61 | 5.38 | 7.50 | -192.10 | |
| JR01F.2010.1.L.004 | MUSCLE | NO | YES | 8.92 | 5.07 | 71.04 | -25.08 | 7.96 |  |  | |
| JR01F.2010.1.L.005 | MUSCLE | NO | NO | 8.57 | 4.30 | 56.07 | -25.40 | 6.54 | 7.90 | -209.80 | |
| JR01F.2010.1.L.005 | MUSCLE | NO | YES | 13.16 | 4.44 | 69.79 | -30.22 | 5.30 |  |  | |
| JR01F.2010.1.L.006 | MUSCLE | NO | NO | 9.41 | 3.71 | 53.93 | -27.36 | 5.73 | 7.50 | -198.80 | |
| JR01F.2010.1.L.007 | MUSCLE | NO | NO | 9.59 | 3.74 | 56.59 | -27.01 | 5.90 | 7.70 | -204.10 | |
| JR01F.2010.1.L.008 | MUSCLE | NO | NO | 10.34 | 3.54 | 49.47 | -30.48 | 4.79 | 7.70 | -196.70 | |
| JR01F.2010.1.L.009 | MUSCLE | NO | NO | 11.84 | 3.55 | 51.56 | -32.08 | 4.36 |  |  | |
| JR01F.2010.1.L.010 | MUSCLE | NO | NO | 9.61 | 3.67 | 54.76 | -26.15 | 5.70 | 7.50 | -196.70 | |
| JR01F.2010.1.L.010 | MUSCLE | NO | YES |  |  |  |  |  | 7.40 | -193.60 | |
| JR01F.2010.1.L.011 | MUSCLE | NO | NO | 10.54 | 4.33 | 53.40 | -27.67 | 5.06 | 7.60 | -194.00 | |
| JR01F.2010.1.L.012 | MUSCLE | NO | NO | 10.32 | 3.38 | 55.69 | -28.45 | 5.40 | 7.20 | -182.80 | |
| JR01F.2010.1.L.013 | MUSCLE | NO | NO | 9.78 | 3.85 | 46.39 | -28.73 | 4.74 |  |  | |
| JR01F.2010.1.L.014 | MUSCLE | NO | NO | 10.64 | 3.64 | 49.19 | -29.76 | 4.62 |  |  | |
| JR01F.2010.1.L.015 | MUSCLE | NO | NO | 10.62 | 4.06 | 52.55 | -29.67 | 4.95 |  |  | |
| JR01F.2010.1.L.016 | MUSCLE | NO | NO | 8.41 | 3.81 | 55.34 | -25.46 | 6.58 | 8.00 | -208.30 | |
| JR01F.2010.1.L.017 | MUSCLE | NO | NO | 10.32 | 3.52 | 52.11 | -28.81 | 5.05 | 7.40 | -191.00 | |
| JR01F.2010.1.L.018 | MUSCLE | NO | NO | 8.09 | 5.41 | 46.84 | -25.69 | 5.79 | 7.10 | -191.90 | |
| JR01F.2010.1.L.019 | MUSCLE | NO | NO | 10.42 | 3.05 | 53.90 | -29.88 | 5.17 | 6.90 | -176.40 | |
| JR01F.2010.1.L.020 | MUSCLE | NO | NO | 11.30 | 6.08 | 48.70 | -29.57 | 4.31 |  |  | |
| JR02.2010.1.L.017 | MUSCLE | NO | NO | 9.78 | 3.38 | 54.91 | -26.33 | 5.62 |  |  | |
| JR02.2010.1.L.017 | MUSCLE | NO | YES | 9.66 | 3.56 | 53.86 | -26.49 | 5.58 |  |  | |
| JR02.2010.1.L.018 | MUSCLE | NO | NO | 8.59 | 2.46 | 56.74 | -25.14 | 6.61 |  |  | |
| JR02.2010.1.L.019 | MUSCLE | NO | NO | 7.33 | 2.81 | 56.79 | -24.88 | 7.75 |  |  | |
| JR02.2010.1.L.020 | MUSCLE | NO | NO | 9.19 | 3.23 | 55.63 | -26.52 | 6.06 |  |  | |
| JR02F.2010.1.L.002 | MUSCLE | NO | NO | 12.03 | 3.26 | 51.72 | -30.26 | 4.30 |  |  | |
| JR02F.2010.1.L.005 | MUSCLE | NO | NO | 10.88 | 2.80 | 52.58 | -31.57 | 4.83 |  |  | |
| JR02F.2010.1.L.010 | MUSCLE | NO | NO | 8.81 | 2.23 | 54.31 | -27.14 | 6.17 |  |  | |
| JR02F.2010.1.L.001 | MUSCLE | NO | NO | 6.59 | 2.20 | 62.31 | -23.50 | 9.46 |  |  | |
| JR02F.2010.1.L.003 | MUSCLE | NO | NO | 9.52 | 2.89 | 53.44 | -28.00 | 5.61 |  |  | |
| JR02F.2010.1.L.004 | MUSCLE | NO | NO | 8.70 | 2.95 | 52.21 | -30.27 | 6.00 |  |  | |
| JR02F.2010.1.L.009 | MUSCLE | NO | NO | 12.09 | 3.19 | 50.19 | -30.75 | 4.15 |  |  | |
| JR02F.2010.1.L.006 | MUSCLE | NO | NO | 6.97 | 2.53 | 58.70 | -27.46 | 8.42 |  |  | |
| JR02F.2010.1.L.007 | MUSCLE | NO | NO | 11.15 | 4.11 | 51.49 | -29.60 | 4.62 |  |  | |
| JR02F.2010.1.L.008 | MUSCLE | NO | NO | 8.97 | 1.88 | 54.38 | -28.18 | 6.06 |  |  | |
| PR01.2010.1.L.001 | MUSCLE | NO | NO | 11.93 | 4.76 | 69.52 | -27.35 | 5.83 |  |  | |
| PR01.2010.1.L.002 | MUSCLE | NO | NO | 10.03 | 3.85 | 55.31 | -24.92 | 5.51 |  |  | |
| PR01.2010.1.L.003 | MUSCLE | NO | NO | 6.56 | 5.44 | 35.51 | -27.62 | 5.42 |  |  | |
| PR01.2010.1.L.004 | MUSCLE | NO | NO | 6.37 | 5.49 | 41.32 | -26.27 | 6.49 |  |  | |
| PR01.2010.1.L.005 | MUSCLE | NO | NO | 8.46 | 4.82 | 58.31 | -23.22 | 6.89 |  |  | |
| PR01F.2010.1.L.001 | MUSCLE | NO | NO | 15.86 | 5.27 | 89.66 | -25.88 | 5.65 | 7.60 | -195.10 | |
| PR01F.2010.1.L.002 | MUSCLE | NO | NO | 9.72 | 3.87 | 53.82 | -26.12 | 5.54 | 7.60 | -193.80 | |
| PR01F.2010.1.L.003 | MUSCLE | NO | NO | 7.21 | 4.90 | 61.22 | -24.36 | 8.49 | 8.00 | -198.30 | |
| PR01F.2010.1.L.004 | MUSCLE | NO | NO | 7.13 | 4.12 | 55.31 | -23.73 | 7.76 | 7.10 | -182.10 | |
| PR01F.2010.1.L.004 | MUSCLE | NO | YES |  |  |  |  |  | 9.00 | -229.50 | |
| PR01F.2010.1.L.004 | MUSCLE | NO | YES |  |  |  |  |  | 8.50 | -230.20 | |
| PR01F.2010.1.L.005 | MUSCLE | NO | NO | 10.11 | 5.13 | 72.49 | -26.30 | 7.17 | 7.50 | -185.10 | |
| PR01F.2010.1.L.006 | MUSCLE | NO | NO | 10.17 | 4.52 | 55.94 | -27.25 | 5.50 | 7.30 | -186.00 | |
| PR01F.2010.1.L.007 | MUSCLE | NO | NO | 12.78 | 4.88 | 50.07 | -30.93 | 3.92 | 6.20 | -165.50 | |
| PR01F.2010.1.L.008 | MUSCLE | NO | NO | 11.69 | 5.38 | 51.77 | -29.71 | 4.43 | 8.50 | -220.80 | |
| PR01F.2010.1.L.009 | MUSCLE | NO | NO | 6.25 | 3.78 | 62.26 | -22.41 | 9.96 | 8.60 | -210.90 | |
| PR01F.2010.1.L.009 | MUSCLE | NO | YES | 7.01 | 3.94 | 62.59 | -22.49 | 8.93 | 8.20 | -211.20 | |
| PR01F.2010.1.L.010 | MUSCLE | NO | NO | 9.12 | 4.53 | 56.52 | -26.06 | 6.20 | 7.60 | -196.50 | |
| PR01F.2010.1.L.011 | MUSCLE | NO | NO | 11.63 | 5.12 | 52.55 | -28.02 | 4.52 | 7.60 | -196.90 | |
| PR01F.2010.1.L.012 | MUSCLE | NO | NO | 7.32 | 5.38 | 61.05 | -23.06 | 8.34 | 8.70 | -213.10 | |
| PR01F.2010.1.L.012 | MUSCLE | NO | YES |  |  |  |  |  | 8.50 | -211.90 | |
| PR01F.2010.1.L.013 | MUSCLE | NO | NO | 5.41 | 3.88 | 34.44 | -25.50 | 6.36 | 8.30 | -203.80 | |
| PR01F.2010.1.L.014 | MUSCLE | NO | NO | 9.79 | 3.68 | 54.92 | -27.95 | 5.61 | 7.40 | -198.30 | |
| MR01.2010.1.L.001 | MUSCLE | NO | NO | 8.56 | 7.02 | 57.48 | -27.03 | 6.72 |  |  | |
| MR01.2010.1.L.002 | MUSCLE | NO | NO | 10.22 | 6.88 | 52.97 | -27.19 | 5.18 |  |  | |
| MR01.2010.1.L.002 | MUSCLE | NO | YES | 9.70 | 6.82 | 55.12 | -27.14 | 5.68 |  |  | |
| MR01.2010.1.L.003 | MUSCLE | NO | NO | 10.57 | 7.64 | 54.70 | -27.06 | 5.18 |  |  | |
| MR01.2010.1.L.004 | MUSCLE | NO | NO | 11.81 | 7.54 | 52.22 | -28.48 | 4.42 |  |  | |
| MR01.2010.1.L.005 | MUSCLE | NO | NO | 11.83 | 8.24 | 49.78 | -27.70 | 4.21 |  |  | |
| MR01.2010.1.L.006 | MUSCLE | NO | NO | 9.61 | 8.13 | 55.52 | -27.14 | 5.78 |  |  | |
| MR01.2010.1.L.006 | MUSCLE | NO | YES | 9.56 | 8.22 | 55.99 | -27.19 | 5.86 |  |  | |
| MR01.2010.1.L.007 | MUSCLE | NO | NO | 8.57 | 7.23 | 58.31 | -25.36 | 6.81 |  |  | |
| MR01.2010.1.L.008 | MUSCLE | NO | NO | 10.35 | 7.89 | 54.34 | -26.63 | 5.25 |  |  | |
| MR01.2010.1.L.009 | MUSCLE | NO | NO | 12.72 | 7.91 | 50.83 | -27.35 | 4.00 |  |  | |
| MR01.2010.1.L.010 | MUSCLE | NO | NO | 11.64 | 7.89 | 51.30 | -27.95 | 4.41 |  |  | |
| MR01.2010.1.L.010 | MUSCLE | NO | YES | 11.61 | 7.72 | 51.84 | -27.98 | 4.47 |  |  | |
| MR01.2010.1.L.011 | MUSCLE | NO | NO | 8.80 | 8.05 | 57.72 | -25.51 | 6.56 |  |  | |
| MR01.2010.1.L.012 | MUSCLE | NO | NO | 10.12 | 7.41 | 47.53 | -27.56 | 4.70 |  |  | |
| MR01F.2010.1.L.001 | MUSCLE | NO | NO | 13.16 | 8.44 | 49.08 | -28.80 | 3.73 | 6.10 | -138.30 | |
| MR01F.2010.1.L.002 | MUSCLE | NO | NO | 13.52 | 7.75 | 48.92 | -28.67 | 3.62 | 6.20 | -141.40 | |
| MR01F.2010.1.L.003 | MUSCLE | NO | NO | 11.46 | 7.73 | 52.55 | -28.70 | 4.59 | 7.00 | -168.50 | |
| MR01F.2010.1.L.003 | MUSCLE | NO | YES |  |  |  |  |  | 6.80 | -167.00 | |
| MR01F.2010.1.L.004 | MUSCLE | NO | NO | 13.97 | 7.77 | 48.74 | -29.31 | 3.49 | 6.20 | -142.00 | |
| MR01F.2010.1.L.005 | MUSCLE | NO | NO | 13.59 | 8.16 | 48.77 | -29.60 | 3.59 | 6.10 | -149.80 | |
| MR01F.2010.1.L.006 | MUSCLE | NO | NO | 12.64 | 7.96 | 50.38 | -28.68 | 3.99 | 6.50 | -149.30 | |
| MR01F.2010.1.L.007 | MUSCLE | NO | NO | 13.93 | 8.00 | 48.97 | -29.51 | 3.52 | 6.20 | -147.10 | |
| MR01F.2010.1.L.008 | MUSCLE | NO | NO | 10.28 | 7.87 | 56.31 | -26.58 | 5.48 | 7.20 | -183.10 | |
| MR01F.2010.1.L.008 | MUSCLE | NO | YES | 10.40 | 7.92 | 55.70 | -26.64 | 5.36 |  |  | |
| MR02.2010.1.L.001 | MUSCLE | NO | NO | 9.67 | 8.21 | 56.90 | -25.27 | 5.89 |  |  | |
| MR02.2010.1.L.002 | MUSCLE | NO | NO | 7.85 | 6.84 | 59.78 | -22.81 | 7.61 |  |  | |
| MR02.2010.1.L.003 | MUSCLE | NO | NO | 8.42 | 7.34 | 59.17 | -23.21 | 7.03 |  |  | |
| MR02.2010.1.L.004 | MUSCLE | NO | NO | 7.56 | 7.35 | 61.21 | -23.69 | 8.10 |  |  | |
| MR02.2010.1.L.005 | MUSCLE | NO | NO | 7.98 | 6.99 | 61.39 | -22.96 | 7.70 |  |  | |
| MR02.2010.1.L.006 | MUSCLE | NO | NO | 8.27 | 7.21 | 59.35 | -26.09 | 7.18 |  |  | |
| MR02.2010.1.L.007 | MUSCLE | NO | NO | 8.09 | 7.46 | 60.06 | -25.68 | 7.42 |  |  | |
| MR02.2010.1.L.008 | MUSCLE | NO | NO | 8.93 | 7.91 | 58.54 | -25.25 | 6.56 |  |  | |
| MR02.2010.1.L.009 | MUSCLE | NO | NO | 8.37 | 7.47 | 59.32 | -24.34 | 7.09 |  |  | |
| MR02.2010.1.L.010 | MUSCLE | NO | NO | 9.86 | 7.24 | 56.80 | -24.83 | 5.76 |  |  | |
| MR02.2010.1.L.011 | MUSCLE | NO | NO | 8.04 | 7.51 | 58.68 | -24.67 | 7.30 |  |  | |
| MR02.2010.1.L.013 | MUSCLE | NO | NO | 10.36 | 7.97 | 54.60 | -26.55 | 5.27 |  |  | |
| MR02F.2010.1.L.001 | MUSCLE | NO | NO | 13.58 | 8.34 | 49.72 | -27.50 | 3.66 | 6.40 | -138.70 | |
| MR02F.2010.1.L.002 | MUSCLE | NO | YES | 10.03 | 6.52 | 55.32 | -24.72 | 5.51 | 6.90 | -184.10 | |
| MR02F.2010.1.L.002 | MUSCLE | NO | YES | 10.07 | 6.42 | 55.41 | -24.66 | 5.51 |  |  | |
| MR02F.2010.1.L.003 | MUSCLE | NO | NO | 13.25 | 8.26 | 47.18 | -27.63 | 3.56 | 6.00 | -130.50 | |
| MR02F.2010.1.L.004 | MUSCLE | NO | NO | 10.36 | 7.56 | 55.15 | -26.05 | 5.32 | 7.40 | -180.60 | |
| MR02F.2010.1.L.005 | MUSCLE | NO | NO | 12.93 | 7.85 | 48.34 | -27.16 | 3.74 | 6.00 | -135.10 | |
| MR02F.2010.1.L.006 | MUSCLE | NO | NO | 11.08 | 7.34 | 52.99 | -25.23 | 4.78 | 6.90 | -164.60 | |
| MR02F.2010.1.L.007 | MUSCLE | NO | NO | 9.16 | 7.26 | 56.53 | -23.17 | 6.17 | 7.80 | -177.00 | |
| MR02F.2010.1.L.008 | MUSCLE | NO | NO | 12.41 | 7.59 | 50.32 | -26.80 | 4.05 | 6.80 | -159.40 | |
| TB01.2017.1.L.001 | MUSCLE | NO | NO | 8.60 | 2.98 | 59.92 | -24.80 | 6.97 |  |  | |
| TB01.2017.1.L.002 | MUSCLE | NO | NO | 14.69 | 4.41 | 50.36 | -27.50 | 3.43 |  |  | |
| TB01.2017.1.L.003 | MUSCLE | NO | NO | 7.40 | 1.82 | 63.77 | -23.90 | 8.62 |  |  | |
| TB01.2017.1.L.004 | MUSCLE | NO | NO | 11.95 | 1.20 | 50.54 | -30.02 | 4.23 |  |  | |
| TB01.2017.1.L.007 | MUSCLE | NO | NO | 9.53 | 0.77 | 55.33 | -30.25 | 5.81 |  |  | |
| TB02.2017.1.L.001 | MUSCLE | NO | NO | 14.17 | 7.53 | 49.59 | -26.04 | 3.50 |  |  | |
| TB02.2017.1.L.002 | MUSCLE | NO | NO | 11.98 | 6.99 | 54.79 | -24.94 | 4.57 |  |  | |
| TB02.2017.1.L.003 | MUSCLE | NO | NO | 12.97 | 7.39 | 55.54 | -25.12 | 4.28 |  |  | |
| TB02.2017.1.L.004 | MUSCLE | NO | NO | 13.38 | 7.89 | 52.73 | -25.60 | 3.94 |  |  | |
| TB02.2017.1.L.005 | MUSCLE | NO | NO | 9.94 | 6.59 | 57.16 | -24.21 | 5.75 |  |  | |
| TB02.2017.1.L.006 | MUSCLE | NO | NO | 11.11 | 6.60 | 56.80 | -23.51 | 5.11 |  |  | |
| TB02.2017.1.L.007 | MUSCLE | NO | NO | 12.10 | 7.39 | 55.60 | -24.17 | 4.60 |  |  | |
| TB02.2017.1.L.008 | MUSCLE | NO | NO | 12.57 | 8.20 | 54.82 | -25.33 | 4.36 |  |  | |
| TB02.2017.1.L.010 | MUSCLE | NO | NO | 11.11 | 6.70 | 55.44 | -23.84 | 4.99 |  |  | |
| TB02.2017.1.L.012 | MUSCLE | NO | NO | 11.66 | 8.07 | 57.26 | -24.68 | 4.91 |  |  | |
| TB02.2017.1.L.015 | MUSCLE | NO | NO | 9.97 | 6.69 | 57.75 | -23.42 | 5.79 |  |  | |
| TB02.2017.1.L.016 | MUSCLE | NO | NO | 11.39 | 7.46 | 56.59 | -25.21 | 4.97 |  |  | |
| GR03.2017.1.L.001 | MUSCLE | NO | NO | 9.04 | 6.07 | 57.32 | -21.50 | 6.34 | 7.71 | -172.59 | |
| GR03.2017.1.L.002 | MUSCLE | NO | NO | 15.04 | 7.14 | 64.27 | -25.70 | 4.27 | 6.88 | -157.52 | |
| GR03.2017.1.L.003 | MUSCLE | NO | NO | 9.47 | 6.18 | 54.90 | -21.08 | 5.80 | 6.92 | -163.92 | |
| GR03.2017.1.L.004 | MUSCLE | NO | NO | 8.88 | 5.81 | 54.71 | -22.98 | 6.16 | 6.82 | -167.31 | |
| GR03.2017.1.L.005 | MUSCLE | NO | NO | 11.46 | 6.26 | 50.95 | -23.38 | 4.45 | 6.64 | -162.70 | |
| GR03.2017.1.L.012 | MUSCLE | NO | NO | 12.19 | 7.60 | 55.70 | -25.19 | 4.57 | 6.69 | -190.00 | |
| GR03.2017.1.L.013 | MUSCLE | NO | NO | 10.43 | 7.30 | 57.59 | -25.98 | 5.52 | 7.09 | -182.47 | |
| GR03.2017.1.L.014 | MUSCLE | NO | NO | 12.51 | 7.28 | 50.10 | -27.25 | 4.00 | 6.92 | -164.85 | |
| GR03.2017.1.L.015 | MUSCLE | NO | NO | 12.73 | 7.91 | 54.25 | -24.51 | 4.26 | 6.74 | -174.96 | |
| GR03.2017.1.L.016 | MUSCLE | NO | NO | 12.18 | 8.40 | 55.48 | -25.42 | 4.56 | 7.00 | -186.59 | |
| GR03.2017.1.L.017 | MUSCLE | NO | NO | 12.93 | 8.18 | 52.76 | -24.74 | 4.08 | 6.74 | -176.40 | |
| GR03.2017.1.L.018 | MUSCLE | NO | NO | 13.62 | 7.55 | 51.89 | -25.09 | 3.81 | 6.61 | -172.03 | |
| GR03.2017.1.L.019 | MUSCLE | NO | NO | 13.42 | 7.96 | 51.43 | -25.93 | 3.83 | 6.42 | -172.70 | |
| GR03.2017.1.L.020 | MUSCLE | NO | NO | 12.79 | 9.02 | 52.30 | -26.11 | 4.09 | 6.39 | -171.48 | |
| GR03.2017.1.L.032 | MUSCLE | NO | NO | 12.98 | 7.03 | 53.06 | -25.46 | 4.09 | 6.54 | -166.09 | |
| GR03.2017.1.L.033 | MUSCLE | NO | NO | 10.62 | 6.90 | 57.75 | -24.37 | 5.44 | 6.93 | -177.22 | |
| GR03.2017.1.L.039 | MUSCLE | NO | NO | 10.53 | 6.97 | 57.55 | -25.45 | 5.47 | 6.63 | -182.97 | |
| GR03.2017.1.L.041 | MUSCLE | NO | NO | 13.56 | 7.42 | 53.34 | -26.15 | 3.93 | 7.06 | -167.50 | |
| GR03.2017.1.L.043 | MUSCLE | NO | NO | 13.67 | 6.69 | 51.59 | -25.74 | 3.77 | 6.40 | -166.58 | |
| GR03.2017.1.L.044 | MUSCLE | NO | NO | 12.46 | 7.43 | 54.09 | -24.52 | 4.34 | 6.96 | -182.94 | |
| GR03.2017.1.L.046 | MUSCLE | NO | NO | 13.72 | 7.35 | 52.06 | -25.65 | 3.79 | 6.25 | -170.27 | |
| GR03.2017.1.L.052 | MUSCLE | NO | NO | 11.09 | 8.15 | 56.67 | -24.77 | 5.11 | 6.92 | -179.96 | |
| GR03.2017.1.L.053 | MUSCLE | NO | NO | 11.33 | 6.26 | 44.71 | -25.45 | 3.95 | 8.34 | -200.88 | |
| GR03.2017.1.L.013 | MUSCLE | YES | NO | 13.50 | 7.74 | 44.20 | -26.67 | 3.27 | 4.84 | -157.75 | |
| GR03.2017.1.L.014 | MUSCLE | YES | NO | 13.87 | 7.69 | 45.78 | -28.04 | 3.30 | 5.14 | -159.21 | |
| GR03.2017.1.L.015 | MUSCLE | YES | NO | 13.82 | 8.24 | 46.38 | -25.38 | 3.36 | 5.78 | -149.99 | |
| GR03.2017.1.L.016 | MUSCLE | YES | NO | 14.48 | 8.84 | 46.40 | -26.96 | 3.20 | 5.21 | -151.16 | |
| GR03.2017.1.L.017 | MUSCLE | YES | NO | 14.47 | 8.61 | 46.80 | -25.01 | 3.23 | 5.71 | -166.77 | |
| GR03.2017.1.L.018 | MUSCLE | YES | NO | 14.68 | 8.03 | 47.23 | -25.81 | 3.22 | 5.48 | -153.51 | |
| GR03.2017.1.L.019 | MUSCLE | YES | NO | 13.60 | 8.47 | 44.29 | -26.59 | 3.26 | 4.96 | -162.01 | |
| GR03.2017.1.L.020 | MUSCLE | YES | NO | 13.24 | 9.43 | 44.62 | -26.37 | 3.37 | 5.27 | -154.31 | |
| GR03.2017.1.L.032 | MUSCLE | YES | NO | 13.86 | 7.35 | 45.27 | -25.88 | 3.27 | 5.67 | -158.61 | |
| GR03.2017.1.L.039 | MUSCLE | YES | NO | 14.43 | 7.29 | 46.65 | -25.74 | 3.23 | 6.23 | -154.03 | |
| GR03.2017.1.L.041 | MUSCLE | YES | NO | 13.91 | 7.61 | 45.09 | -26.81 | 3.24 | 5.96 | -156.56 | |
| GR03.2017.1.L.044 | MUSCLE | YES | NO | 13.90 | 6.50 | 42.88 | -25.84 | 3.08 | 5.36 | -158.41 | |
| GR03.2017.1.L.046 | MUSCLE | YES | NO | 14.46 | 7.77 | 46.83 | -25.89 | 3.24 | 6.55 | -156 | |
| GR03.2017.1.L.052 | MUSCLE | YES | NO | 13.09 | 8.57 | 43.01 | -26.19 | 3.29 | 4.55 | -161.6 | |
| GR03.2017.1.L.053 | MUSCLE | YES | NO | 13.20 | 7.59 | 44.26 | -26.33 | 3.35 | 5.20 | -153.5 | |
| GR04.2017.1.L.001 | MUSCLE | NO | NO | 13.00 | 6.77 | 47.87 | -25.70 | 3.68 |  |  | |
| GR04.2017.1.L.002 | MUSCLE | NO | NO | 11.28 | 6.76 | 40.55 | -26.01 | 3.59 |  |  | |
| GR04.2017.1.L.003 | MUSCLE | NO | NO | 11.73 | 5.64 | 46.84 | -24.53 | 3.99 |  |  | |
| GR04.2017.1.L.004 | MUSCLE | NO | NO | 13.64 | 6.17 | 49.26 | -26.17 | 3.61 |  |  | |
| GR04.2017.1.L.005 | MUSCLE | NO | NO | 13.85 | 6.95 | 49.09 | -27.88 | 3.54 |  |  | |
| GR06.2017.1.L.001 | MUSCLE | NO | NO | 12.99 | 6.20 | 48.91 | -26.64 | 3.77 |  |  | |
| GR06.2017.1.L.003 | MUSCLE | NO | NO | 11.29 | 6.35 | 52.65 | -26.25 | 4.66 |  |  | |
| GR06.2017.1.L.004 | MUSCLE | NO | NO | 13.02 | 7.13 | 49.99 | -26.23 | 3.84 |  |  | |
| GR06.2017.1.L.005 | MUSCLE | NO | NO | 12.95 | 6.69 | 47.51 | -27.26 | 3.67 |  |  | |
| GR06.2017.1.L.006 | MUSCLE | NO | NO | 11.22 | 7.24 | 53.74 | -27.68 | 4.79 |  |  | |
| GR06.2017.1.L.008 | MUSCLE | NO | NO | 6.73 | 6.85 | 59.02 | -22.30 | 8.77 |  |  | |
| GR06.2017.1.L.009 | MUSCLE | NO | NO | 8.68 | 6.86 | 60.51 | -19.28 | 6.97 |  |  | |
| GR06.2017.1.L.010 | MUSCLE | NO | NO | 8.67 | 6.97 | 59.85 | -20.58 | 6.91 |  |  | |
| GR06.2017.1.L.011 | MUSCLE | NO | NO | 10.66 | 8.48 | 55.35 | -20.75 | 5.19 |  |  | |
| GR06.2017.1.L.012 | MUSCLE | NO | NO | 8.96 | 6.11 | 54.68 | -21.61 | 6.10 |  |  | |
| GR06.2017.1.L.013 | MUSCLE | NO | NO | 6.92 | 5.79 | 63.38 | -20.81 | 9.16 |  |  | |
| GR06.2017.1.L.020 | MUSCLE | NO | NO | 4.17 | 6.68 | 69.61 | -19.22 | 16.68 |  |  | |
| GR06.2017.1.L.021 | MUSCLE | NO | NO | 5.36 | 7.36 | 64.84 | -21.58 | 12.10 |  |  | |
| GR06.2017.1.L.004 | MUSCLE | YES | NO | 13.02 | 7.18 | 43.10 | -26.12 | 3.31 | 6.00 | -159.39 | |
| GR06.2017.1.L.008 | MUSCLE | YES | NO | 13.12 | 7.37 | 46.00 | -24.33 | 3.51 | 6.35 | -149.60 | |
| GR06.2017.1.L.009 | MUSCLE | YES | NO | 13.58 | 6.77 | 43.31 | -21.68 | 3.19 | 6.39 | -161.33 | |
| GR06.2017.1.L.010 | MUSCLE | YES | NO | 13.08 | 7.17 | 43.08 | -22.83 | 3.29 | 6.31 | -155.03 | |
| GR06.2017.1.L.011 | MUSCLE | YES | NO | 13.92 | 8.73 | 44.50 | -23.26 | 3.20 | 6.37 | -155.85 | |
| GR06.2017.1.L.012 | MUSCLE | YES | NO | 13.69 | 6.69 | 45.00 | -22.44 | 3.29 | 5.99 | -162.56 | |
| GR06.2017.1.L.013 | MUSCLE | YES | NO | 14.61 | 6.14 | 47.74 | -22.84 | 3.27 | 6.35 | -152.57 | |
| GR06.2017.1.L.020 | MUSCLE | YES | NO | 14.04 | 7.26 | 45.53 | -22.97 | 3.24 | 6.51 | -165.34 | |
| GR06.2017.1.L.021 | MUSCLE | YES | NO | 13.63 | 8.01 | 45.24 | -26.11 | 3.32 | 6.40 | -147.10 | |
| GR07.2017.1.L.001 | MUSCLE | NO | NO | 12.75 | 8.43 | 49.42 | -28.99 | 3.88 |  |  | |
| GR07.2017.1.L.002 | MUSCLE | NO | NO | 13.91 | 8.47 | 47.62 | -25.70 | 3.42 |  |  | |
| GR07.2017.1.L.003 | MUSCLE | NO | NO | 13.68 | 8.30 | 47.18 | -25.56 | 3.45 |  |  | |
| GR07.2017.1.L.004 | MUSCLE | NO | NO | 8.86 | 8.49 | 30.24 | -26.39 | 3.42 |  |  | |
| GR07.2017.1.L.005 | MUSCLE | NO | NO | 14.06 | 7.74 | 47.92 | -25.32 | 3.41 |  |  | |
| GR07.2017.1.L.018 | MUSCLE | NO | NO | 13.46 | 8.51 | 48.90 | -23.67 | 3.63 |  |  | |
| GR07.2017.1.L.020 | MUSCLE | NO | NO | 9.55 | 8.13 | 56.68 | -25.89 | 5.93 |  |  | |
| GR07.2017.1.L.022 | MUSCLE | NO | NO | 7.59 | 7.24 | 59.03 | -23.35 | 7.78 |  |  | |
| GR07.2017.1.L.024 | MUSCLE | NO | NO | 8.50 | 7.79 | 56.69 | -23.23 | 6.67 |  |  | |
| GR07.2017.1.L.026 | MUSCLE | NO | NO | 12.60 | 7.48 | 46.58 | -25.18 | 3.70 |  |  | |
| GR07.2017.1.L.028 | MUSCLE | NO | NO | 9.72 | 6.46 | 52.69 | -24.48 | 5.42 |  |  | |
| GR07.2017.1.L.030 | MUSCLE | NO | NO | 10.13 | 7.14 | 54.74 | -25.79 | 5.40 |  |  | |
| GR07.2017.1.L.032 | MUSCLE | NO | NO | 11.76 | 8.14 | 52.90 | -25.25 | 4.50 |  |  | |
| GR07.2017.1.L.034 | MUSCLE | NO | NO | 8.84 | 7.60 | 58.95 | -25.22 | 6.67 |  |  | |
| GR07.2017.1.L.036 | MUSCLE | NO | NO | 12.61 | 8.43 | 53.24 | -25.18 | 4.22 |  |  | |
| GR07.2017.1.L.049 | MUSCLE | NO | NO | 10.76 | 6.74 | 55.37 | -25.18 | 5.15 |  |  | |
| GR07.2017.1.L.022 | MUSCLE | YES | NO | 12.64 | 7.64 | 44.97 | -26.07 | 3.56 | 6.29 | -149.94 | |
| GR07.2017.1.L.024 | MUSCLE | YES | NO | 13.32 | 8.57 | 45.37 | -25.80 | 3.41 | 6.28 | -149.77 | |
| GR07.2017.1.L.028 | MUSCLE | YES | NO | 12.65 | 7.01 | 43.18 | -25.18 | 3.41 | 6.43 | -155.84 | |
| GR07.2017.1.L.034 | MUSCLE | YES | NO | 13.02 | 8.06 | 43.81 | -26.49 | 3.36 | 6.34 | -153.84 | |
| GR07.2017.1.L.049 | MUSCLE | YES | NO | 13.38 | 7.01 | 43.71 | -25.02 | 3.27 | 6.30 | -168.39 | |
| GR08.2017.1.L.001 | MUSCLE | NO | NO | 13.20 | 8.74 | 52.50 | -23.85 | 3.98 |  |  | |
| GR08.2017.1.L.002 | MUSCLE | NO | NO | 11.35 | 7.30 | 53.17 | -22.05 | 4.68 |  |  | |
| GR08.2017.1.L.003 | MUSCLE | NO | NO | 12.42 | 9.39 | 52.90 | -23.58 | 4.26 |  |  | |
| GR08.2017.1.L.004 | MUSCLE | NO | NO | 10.15 | 7.45 | 54.90 | -23.00 | 5.41 |  |  | |
| GR08.2017.1.L.005 | MUSCLE | NO | NO | 10.85 | 8.39 | 53.24 | -22.67 | 4.91 |  |  | |
| GR08.2017.1.L.006 | MUSCLE | NO | NO | 11.17 | 7.25 | 56.75 | -22.01 | 5.08 |  |  | |
| GR08.2017.1.L.007 | MUSCLE | NO | NO | 12.17 | 8.86 | 52.66 | -22.68 | 4.33 |  |  | |
| GR08.2017.1.L.009 | MUSCLE | NO | NO | 10.41 | 7.84 | 53.14 | -22.26 | 5.11 |  |  | |
| GR08.2017.1.L.010 | MUSCLE | NO | NO | 12.45 | 9.66 | 52.32 | -24.19 | 4.20 |  |  | |
| GR08.2017.1.L.011 | MUSCLE | NO | NO | 11.81 | 7.36 | 52.81 | -23.21 | 4.47 |  |  | |
| GR08.2017.1.L.012 | MUSCLE | NO | NO | 12.89 | 7.23 | 46.35 | -25.26 | 3.59 |  |  | |
| GR08.2017.1.L.013 | MUSCLE | NO | NO | 13.16 | 7.34 | 48.24 | -25.40 | 3.66 |  |  | |
| GR08.2017.1.L.014 | MUSCLE | NO | NO | 12.00 | 7.81 | 47.65 | -24.94 | 3.97 |  |  | |
| GR08.2017.1.L.015 | MUSCLE | NO | NO | 12.88 | 8.01 | 47.52 | -24.84 | 3.69 |  |  | |
| GR08.2017.1.L.018 | MUSCLE | NO | NO | 11.97 | 9.64 | 49.27 | -24.59 | 4.12 |  |  | |
| GR08.2017.1.L.021 | MUSCLE | NO | NO | 12.62 | 9.13 | 54.06 | -23.05 | 4.28 |  |  | |
| GR08.2017.1.L.022 | MUSCLE | NO | NO | 13.25 | 8.82 | 48.70 | -23.08 | 3.68 |  |  | |
| GR08.2017.1.L.023 | MUSCLE | NO | NO | 10.57 | 8.71 | 55.08 | -23.17 | 5.21 |  |  | |
| GR08.2017.1.L.034 | MUSCLE | NO | NO | 11.87 | 9.55 | 52.80 | -24.78 | 4.45 |  |  | |
| GR08.2017.1.L.035 | MUSCLE | NO | NO | 13.98 | 9.68 | 49.71 | -24.46 | 3.56 |  |  | |
| GR08.2017.1.L.001 | MUSCLE | YES | NO | 14.03 | 9.02 | 45.40 | -24.59 | 3.24 | 6.46 | -137.79 | |
| GR08.2017.1.L.002 | MUSCLE | YES | NO | 13.65 | 8.30 | 45.89 | -22.64 | 3.36 | 6.57 | -169.69 | |
| GR08.2017.1.L.003 | MUSCLE | YES | NO | 13.22 | 10.03 | 44.88 | -23.88 | 3.39 | 6.59 | -173.24 | |
| GR08.2017.1.L.004 | MUSCLE | YES | NO | 13.52 | 8.23 | 44.56 | -23.70 | 3.29 | 6.72 | -166.41 | |
| GR08.2017.1.L.005 | MUSCLE | YES | NO | 13.12 | 8.99 | 43.32 | -24.29 | 3.30 | 6.72 | -168.58 | |
| GR08.2017.1.L.006 | MUSCLE | YES | NO | 13.08 | 7.26 | 42.37 | -23.60 | 3.24 | 6.56 | -167.81 | |
| GR08.2017.1.L.007 | MUSCLE | YES | NO | 13.39 | 8.91 | 43.87 | -23.51 | 3.28 | 6.71 | -169.32 | |
| GR08.2017.1.L.011 | MUSCLE | YES | NO | 13.40 | 7.74 | 43.95 | -23.94 | 3.28 | 6.60 | -164.92 | |
| GR09.2017.1.L.001 | MUSCLE | NO | NO | 13.87 | 8.62 | 47.39 | -25.08 | 3.42 |  |  | |
| GR09.2017.1.L.002 | MUSCLE | NO | NO | 13.72 | 9.65 | 46.73 | -23.26 | 3.41 |  |  | |
| GR09.2017.1.L.003 | MUSCLE | NO | NO | 12.86 | 9.39 | 44.47 | -24.13 | 3.46 |  |  | |
| GR09.2017.1.L.004 | MUSCLE | NO | NO | 14.34 | 9.18 | 50.81 | -24.03 | 3.54 |  |  | |
| GR09.2017.1.L.005 | MUSCLE | NO | NO | 13.41 | 7.76 | 46.48 | -23.91 | 3.46 |  |  | |
| GR09.2017.1.L.011 | MUSCLE | NO | NO | 12.54 | 8.24 | 51.46 | -20.22 | 4.10 |  |  | |
| GR09.2017.1.L.014 | MUSCLE | NO | NO | 10.73 | 8.62 | 55.28 | -19.16 | 5.15 |  |  | |
| GR09.2017.1.L.015 | MUSCLE | NO | NO | 9.74 | 8.31 | 54.61 | -19.07 | 5.61 |  |  | |
| GR09.2017.1.L.016 | MUSCLE | NO | NO | 8.99 | 8.49 | 57.93 | -18.18 | 6.44 |  |  | |
| GR09.2017.1.L.017 | MUSCLE | NO | NO | 9.69 | 8.13 | 54.59 | -19.21 | 5.63 |  |  | |
| GR09.2017.1.L.018 | MUSCLE | NO | NO | 7.06 | 7.88 | 57.09 | -17.97 | 8.08 |  |  | |
| GR09.2017.1.L.019 | MUSCLE | NO | NO | 6.17 | 7.52 | 56.30 | -14.69 | 9.12 |  |  | |
| GR09.2017.1.L.022 | MUSCLE | NO | NO | 9.22 | 8.28 | 53.93 | -15.37 | 5.85 |  |  | |
| GR09.2017.1.L.023 | MUSCLE | NO | NO | 13.49 | 8.75 | 51.18 | -18.16 | 3.79 |  |  | |
| GR09.2017.1.L.024 | MUSCLE | NO | NO | 10.19 | 7.62 | 54.64 | -15.95 | 5.36 |  |  | |
| GR09.2017.1.L.025 | MUSCLE | NO | NO | 10.51 | 8.05 | 54.85 | -18.71 | 5.22 |  |  | |
| GR09.2017.1.L.026 | MUSCLE | NO | NO | 12.62 | 8.12 | 52.91 | -17.04 | 4.19 |  |  | |
| GR09.2017.1.L.027 | MUSCLE | NO | NO | 10.68 | 10.13 | 55.24 | -17.68 | 5.17 |  |  | |
| GR09.2017.1.L.029 | MUSCLE | NO | NO | 7.98 | 8.13 | 55.28 | -18.04 | 6.93 |  |  | |
| GR09.2017.1.L.002 | MUSCLE | YES | NO |  |  |  |  |  | 5.38 | -172.79 | |
| GR09.2017.1.L.004 | MUSCLE | YES | NO |  |  |  |  |  | 6.84 | -155.39 | |
| GR09.2017.1.L.005 | MUSCLE | YES | NO |  |  |  |  |  | 6.08 | -159.65 | |
| GR09.2017.1.L.011 | MUSCLE | YES | NO | 13.55 | 8.60 | 43.97 | -20.80 | 3.25 | 3.7 | -170.03 | |
| GR09.2017.1.L.014 | MUSCLE | YES | NO | 13.84 | 8.84 | 44.93 | -20.46 | 3.25 | 5.49 | -159.24 | |
| GR09.2017.1.L.015 | MUSCLE | YES | NO | 13.95 | 8.43 | 45.22 | -21.38 | 3.24 | 5.69 | -157.93 | |
| GR09.2017.1.L.016 | MUSCLE | YES | NO | 14.49 | 8.98 | 46.29 | -20.43 | 3.19 | 5.20 | -159.59 | |
| GR09.2017.1.L.017 | MUSCLE | YES | NO | 13.91 | 8.62 | 45.71 | -21.64 | 3.29 | 5.39 | -160.61 | |
| GR09.2017.1.L.018 | MUSCLE | YES | NO | 12.82 | 8.59 | 41.45 | -23.44 | 3.23 | 5.48 | -162.36 | |
| GR09.2017.1.L.019 | MUSCLE | YES | NO | 13.53 | 8.48 | 43.75 | -20.69 | 3.23 | 5.3 | -171.95 | |
| GR09.2017.1.L.022 | MUSCLE | YES | NO | 14.11 | 8.67 | 46.00 | -20.49 | 3.26 | 5.3 | -158.08 | |
| GR09.2017.1.L.023 | MUSCLE | YES | NO | 14.74 | 9.34 | 47.40 | -21.39 | 3.22 | 5.13 | -168.49 | |
| GR09.2017.1.L.024 | MUSCLE | YES | NO | 14.02 | 8.01 | 44.98 | -19.80 | 3.21 | 5.58 | -176.68 | |
| GR09.2017.1.L.025 | MUSCLE | YES | NO | 13.86 | 8.60 | 45.20 | -21.98 | 3.26 | 5.33 | -160.72 | |
| GR09.2017.1.L.026 | MUSCLE | YES | NO | 13.04 | 8.68 | 42.09 | -20.50 | 3.23 | 5.23 | -166.42 | |
| GR09.2017.1.L.029 | MUSCLE | YES | NO | 12.57 | 8.37 | 40.41 | -22.89 | 3.21 | 5.64 | -163.13 | |
| GR10.2017.1.L.001 | MUSCLE | NO | NO | 11.99 | 7.94 | 48.88 | -20.70 | 4.08 |  |  | |
| GR10.2017.1.L.002 | MUSCLE | NO | NO | 9.63 | 7.60 | 55.34 | -19.59 | 5.75 |  |  | |
| GR10.2017.1.L.003 | MUSCLE | NO | NO | 7.87 | 6.40 | 60.71 | -20.28 | 7.71 |  |  | |
| GR10.2017.1.L.004 | MUSCLE | NO | NO | 11.55 | 7.18 | 56.96 | -19.76 | 4.93 |  |  | |
| GR10.2017.1.L.005 | MUSCLE | NO | NO | 13.93 | 7.19 | 50.00 | -22.11 | 3.59 |  |  | |
| GR10.2017.1.L.006 | MUSCLE | NO | NO | 10.38 | 7.15 | 55.97 | -21.96 | 5.39 |  |  | |
| GR10.2017.1.L.007 | MUSCLE | NO | NO | 9.94 | 6.55 | 59.94 | -20.74 | 6.03 |  |  | |
| GR10.2017.1.L.008 | MUSCLE | NO | NO | 9.80 | 7.10 | 55.95 | -21.96 | 5.71 |  |  | |
| GR10.2017.1.L.009 | MUSCLE | NO | NO | 8.25 | 6.19 | 61.61 | -19.63 | 7.47 |  |  | |
| GR10.2017.1.L.010 | MUSCLE | NO | NO | 12.37 | 7.43 | 53.73 | -20.72 | 4.34 |  |  | |
| GR10.2017.1.L.011 | MUSCLE | NO | NO | 12.76 | 7.13 | 53.34 | -20.02 | 4.18 |  |  | |
| GR10.2017.1.L.012 | MUSCLE | NO | NO | 13.41 | 6.59 | 52.84 | -23.10 | 3.94 |  |  | |
| GR10.2017.1.L.013 | MUSCLE | NO | NO | 9.78 | 7.19 | 56.42 | -19.93 | 5.77 |  |  | |
| GR10.2017.1.L.014 | MUSCLE | NO | NO | 8.64 | 5.54 | 58.56 | -19.46 | 6.78 |  |  | |
| GR10.2017.1.L.015 | MUSCLE | NO | NO | 9.11 | 6.36 | 55.70 | -18.95 | 6.11 |  |  | |
| GR10.2017.1.L.016 | MUSCLE | NO | NO | 10.65 | 6.51 | 57.44 | -20.64 | 5.39 |  |  | |
| GR10.2017.1.L.017 | MUSCLE | NO | NO | 7.70 | 6.94 | 60.54 | -17.74 | 7.86 |  |  | |
| GR10.2017.1.L.001 | MUSCLE | YES | NO | 13.79 | 8.29 | 45.05 | -21.34 | 3.27 | 6.22 | -166.99 | |
| GR10.2017.1.L.002 | MUSCLE | YES | NO | 13.75 | 7.87 | 44.96 | -21.27 | 3.27 | 7.05 | -166.39 | |
| GR10.2017.1.L.003 | MUSCLE | YES | NO | 13.83 | 6.82 | 45.18 | -23.00 | 3.27 | 7.07 | -156.95 | |
| GR10.2017.1.L.004 | MUSCLE | YES | NO | 14.42 | 7.31 | 46.87 | -21.02 | 3.25 | 7.05 | -167.17 | |
| GR10.2017.1.L.005 | MUSCLE | YES | NO | 13.46 | 7.33 | 44.38 | -22.66 | 3.30 | 6.82 | -161.14 | |
| GR10.2017.1.L.006 | MUSCLE | YES | NO | 14.16 | 7.31 | 46.13 | -23.88 | 3.26 | 7.21 | -165.99 | |
| GR10.2017.1.L.007 | MUSCLE | YES | NO | 14.06 | 6.61 | 46.33 | -22.77 | 3.30 | 7.31 | -156.51 | |
| GR10.2017.1.L.008 | MUSCLE | YES | NO | 13.95 | 7.13 | 46.63 | -24.20 | 3.34 | 7.13 | -155.79 | |
| GR10.2017.1.L.009 | MUSCLE | YES | NO | 13.88 | 6.56 | 45.47 | -22.17 | 3.28 | 7.03 | -160.13 | |
| GR10.2017.1.L.010 | MUSCLE | YES | NO | 13.84 | 7.84 | 44.98 | -21.67 | 3.25 | 7.32 | -167.64 | |
| GR10.2017.1.L.014 | MUSCLE | YES | NO | 14.00 | 5.93 | 45.93 | -21.72 | 3.28 | 6.87 | -161.41 | |
| GR10.2017.1.L.015 | MUSCLE | YES | NO | 13.64 | 6.75 | 44.66 | -21.35 | 3.27 | 7.34 | -165.32 | |
| GR10.2017.1.L.016 | MUSCLE | YES | NO | 14.17 | 6.88 | 45.90 | -21.98 | 3.24 | 7.23 | -165.69 | |
| GR10.2017.1.L.017 | MUSCLE | YES | NO | 13.89 | 7.15 | 45.77 | -20.19 | 3.30 | 7.61 | -162.34 | |
| YR99.2017.1.L.001 | MUSCLE | NO | NO | 11.77 | 2.77 | 54.25 | -18.52 | 4.61 | 7.48 | -212.23 | |
| YR99.2017.1.L.002 | MUSCLE | NO | NO | 10.55 | 3.43 | 56.90 | -18.48 | 5.39 | 8.03 | -225.43 | |
| YR99.2017.1.L.003 | MUSCLE | NO | NO | 11.06 | 5.01 | 53.88 | -18.43 | 4.87 | 6.97 | -207.13 | |
| YR99.2017.1.L.004 | MUSCLE | NO | NO | 11.76 | 2.82 | 53.91 | -18.80 | 4.58 | 7.63 | -222.48 | |
| YR99.2017.1.L.005 | MUSCLE | NO | NO | 12.39 | 4.14 | 50.12 | -19.08 | 4.05 | 7.51 | -197.15 | |
| YR99.2017.1.L.001 | MUSCLE | YES | NO | 14.09 | 2.76 | 44.56 | -19.34 | 3.16 | 7.20 | -167.81 | |
| YR99.2017.1.L.002 | MUSCLE | YES | NO | 14.28 | 3.74 | 44.85 | -19.11 | 3.14 | 7.45 | -181.88 | |
| YR99.2017.1.L.003 | MUSCLE | YES | NO | 14.24 | 5.17 | 44.53 | -18.98 | 3.13 | 6.32 | -177.57 | |
| YR99.2017.1.L.004 | MUSCLE | YES | NO | 14.01 | 2.80 | 44.67 | -19.75 | 3.19 | 7.37 | -177.75 | |
| YR99.2017.1.L.005 | MUSCLE | YES | NO | 14.10 | 4.44 | 44.37 | -19.68 | 3.15 | 6.99 | -176.65 | |

**Table S4.** Site location for lampreys collected in Maryland.

| Site | River name | Date of collection | DO conc. (mg/L) | % DO | Conductivity (µS/cm) | Temp. (°C) |
| --- | --- | --- | --- | --- | --- | --- |
| HR1 | Henderson Run | 9/22/2020 | NA | NA | NA | NA |
| JC1 | Johns Creek | 9/24/2020 | 7.74 | 79.9 | 0.0845 | 17.1 |
| GH1 | Greenhill Run | 9/25/2020 | 7.87 | 82.0 | 0.0432 | 17.8 |
| HR2 | Henderson Run | 12/9/2020 | 12.64 | 102.3 | 0.0711 | 6.4 |
| JC2 | Johns Creek | 12/10/2020 | 11.36 | 93.7 | 0.0855 | 7.6 |
| HR1 | Henderson Run | 3/17/2021 | 11.08 | 94.3 | 0.0553 | 8.6 |
| FR1 | Johns Creek | 3/22/2021 | 9.66 | 87.5 | 0.0727 | 11.4 |
| FR2 | Johns Creek | 5/11/2021 | 8.22 | 77.2 | 0.0571 | 12.9 |
| HR2 | Henderson Run | 5/11/2021 | 8.39 | 79.2 | 0.064 | 13 |
| PR1 | Little Patuxent River | 5/14/2021 | NA | NA | NA | NA |
| DC1 | Deer Creek | 5/24/2021 | NA | NA | NA | NA |
| FB1 | Falling Branch | 5/24/2021 | NA | NA | NA | NA |

**Table S5.** Species, length, and weight for each individual collected in Maryland.

| Individual | Species | Stage | Length (mm) | Wet weight (g) | Sex (M/F/U) | Note |
| --- | --- | --- | --- | --- | --- | --- |
| HR1.2020.3.L.001 | LBL | L | 91 | 1.10 | U |  |
| HR1.2020.2.L.001 | LBL | T | 123 | 3.08 | U |  |
| HR1.2020.2.L.002 | LBL | L | 74 | 0.60 | U |  |
| HR1.2020.1.L.001 | LBL | L | 74 | 0.63 | U |  |
| HR1.2020.1.L.002 | LBL | L | 66 | 0.46 | U |  |
| JC1.2020.3.L.001 | LBL | T | 98 | 1.93 | U |  |
| JC1.2020.2.L.001 | LBL | L | 109 | 2.02 | U |  |
| JC1.2020.1.L.001 | LBL | L | 57 | 0.33 | U |  |
| GH1.2020.1.L.001 | LBL | L | 110 | 2.14 | U | No gut |
| GH1.2020.1.L.002 | LBL | T | 130 | 4.19 | U |  |
| GH1.2020.1.L.003 | LBL | T | 116 | 2.93 | U |  |
| GH1.2020.2.L.001 | LBL | L | 75 | 0.68 | U |  |
| GH1.2020.2.L.002 | LBL | T | 95 | 1.34 | U |  |
| GH1.2020.3.L.001 | LBL | L | 101 | 1.59 | U |  |
| GH1.2020.3.L.002 | LBL | L | 85 | 0.97 | U |  |
| GH1.2020.3.L.003 | LBL | L | 78 | 0.88 | U |  |
| HR2.2020.1.L.001 | LBL | L | 69 | 0.69 | U |  |
| HR2.2020.2.L.001 | LBL | L | 99 | 1.44 | U |  |
| HR2.2020.2.L.002 | LBL | L | 123 | 2.65 | U |  |
| HR2.2020.3.L.001 | LBL | L | 99 | 1.30 | U | Frozen |
| HR2.2020.3.L.002 | LBL | L | 98 | 1.70 | U | Frozen |
| HR2.2020.3.L.003 | LBL | L | 115 | 2.31 | U | Frozen |
| HR2.2020.3.L.004 | LBL | L | 94 | 1.29 | U | Frozen |
| HR2.2020.3.L.005 | LBL | L | 88 | 1.09 | U | Frozen |
| HR2.2020.3.L.006 | LBL | L | 110 | 2.24 | U | Frozen |
| JC2.2020.1.L.001 | LBL | L | 99 | 1.83 | U | Frozen |
| JC2.2020.1.L.002 | LBL | L | 125 | 2.98 | U | Frozen |
| JC2.2020.1.L.003 | LBL | L | 70 | 0.70 | U | Frozen |
| JC2.2020.2.L.001 | LBL | L |  |  | U |  |
| JC2.2020.2.L.002 | LBL | L |  |  | U |  |
| JC2.2020.2.L.003 | LBL | L |  |  | U |  |
| JC2.2020.3.L.001 | LBL | L |  |  | U |  |
| JC2.2020.3.L.002 | LBL | L |  |  | U |  |
| JC2.2020.3.L.003 | LBL | L |  |  | U |  |
| FR1.2021.1.L.001 | LBL | L | 114 | 2.37 | U |  |
| FR1.2021.1.L.002 | LBL | L | 69 | 0.98 | U |  |
| FR1.2021.1.L.003 | LBL | L | 71 | 0.64 | U |  |
| FR1.2021.2.L.001 | LBL | L | 66 | 0.41 | U |  |
| FR1.2021.2.L.002 | LBL | L | 40 |  | U |  |
| FR1.2021.3.L.001 | LBL | L | 119 | 3.67 | U |  |
| FR1.2021.3.L.002 | LBL | L | 90 | 1.50 | U |  |
| HR1.2021.1.L.001 | LBL | L | 131 | 3.21 | U |  |
| HR1.2021.1.L.002 | LBL | L | 85 | 0.89 | U |  |
| HR1.2021.1.L.003 | LBL | L | 90 | 1.03 | U |  |
| HR1.2021.2.L.001 | LBL | L | 135 | 4.09 | U |  |
| HR1.2021.2.L.002 | LBL | L | 87 | 1.06 | U |  |
| HR1.2021.2.L.003 | LBL | L | 97 | 1.46 | U |  |
| HR1.2021.3.L.001 | LBL | L | 73 | 0.61 | U |  |
| HR1.2021.3.L.002 | LBL | L | 83 | 0.79 | U |  |
| FR2.2021.1.L.001 | LBL | L | 84 | 1.10 | U | Frozen |
| FR2.2021.1.L.002 | LBL | L | 80 | 1.64 | U | Frozen |
| FR2.2021.1.L.003 | LBL | L | 86 | 1.16 | U | Frozen |
| FR2.2021.2.L.001 | LBL | L | 83 | 1.64 | U | Frozen |
| FR2.2021.2.L.002 | LBL | L | 70 | 0.74 | U | Frozen |
| FR2.2021.2.L.003 | LBL | L | 93 | 1.21 | U | Frozen |
| FR2.2021.3.L.001 | LBL | L | 115 | 3.02 | U | Frozen |
| FR2.2021.3.L.002 | LBL | L | 62 | 0.51 | U | Frozen |
| FR2.2021.3.L.003 | LBL | L | 64 | 0.54 | U | Frozen |
| HR2.2021.1.L.001 | LBL | L | 125 | 3.72 | U | Frozen |
| HR2.2021.1.L.002 | LBL | L | 82 | 1.49 | U | Frozen |
| HR2.2021.2.L.001 | LBL | L | 121 | 3.65 | U | Frozen |
| HR2.2021.2.L.002 | LBL | L | 91 | 1.40 | U | Frozen |
| HR2.2021.2.L.003 | LBL | L | 90 | 1.30 | U | Frozen |
| HR2.2021.2.L.004 | LBL | L | 45 | 0.23 | U | Frozen |
| HR2.2021.3.L.001 | LBL | L | 111 | 3.74 | U | Frozen |
| HR2.2021.3.L.002 | LBL | L | 66 | 0.77 | U | Frozen |
| HR2.2021.3.L.003 | LBL | L | 66 | 0.93 | U | Frozen |
| PR1.2021.1.L.001 | SL | L | 158 | 7.36 | U | Frozen |
| PR1.2021.1.L.002 | SL | L | 87 | 1.32 | U | Frozen |
| PR1.2021.1.L.003 | SL | L | 132 | 4.50 | U | Frozen |
| PR1.2021.1.L.004 | SL | L | 89 | 1.36 | U | Frozen |
| PR1.2021.1.L.005 | SL | L | 143 | 4.17 | U | Frozen |

Note: LBL is least brook lamprey (*Lampetra aepyptera*)

**Table S6.** For samples collected in Maryland, the list of samples submitted for isotope analysis, whether they were lipid extracted, or duplicated, and measurements of isotope ratios and percentage of each atom in the sample.

| Individual | Tissue | Lipid extracted? | Duplicate | % N | δ^15^N (‰) | % C | δ^13^C (‰) | C:N | % H | δ ^2^H (‰) |
| --- | --- | --- | --- | --- | --- | --- | --- | --- | --- | --- |
| GH1.2020.1.L.003 | MUSCLE | NO | NO | 9.33 | 5.00 | 56.97 | -25.37 | 6.11 |  |  |
| HR1.2020.2.L.002 | MUSCLE | NO | NO | 9.58 | 3.64 | 41.14 | -27.50 | 4.29 |  |  |
| HR1.2020.2.L.001 | MUSCLE | NO | NO | 7.69 | 4.04 | 55.00 | -25.46 | 7.15 |  |  |
| HR1.2020.1.L.001 | MUSCLE | NO | NO | 10.93 | 4.50 | 50.97 | -26.50 | 4.66 |  |  |
| GH1.2020.1.L.002 | MUSCLE | NO | YES | 7.49 | 4.28 | 62.51 | -23.44 | 8.35 |  |  |
| JC1.2020.2.L.001 | MUSCLE | NO | NO | 5.40 | 4.47 | 66.56 | -22.94 | 12.33 |  |  |
| JC1.2020.2.L.001 | MUSCLE | NO | YES | 13.54 | 4.44 | 84.01 | -25.19 | 6.20 |  |  |
| GH1.2020.1.L.001 | MUSCLE | NO | NO | 9.31 | 5.27 | 54.93 | -24.49 | 5.90 |  |  |
| JC1.2020.3.L.001 | MUSCLE | NO | NO | 6.41 | 4.34 | 69.58 | -22.88 | 10.85 |  |  |
| GH1.2020.3.L.002 | MUSCLE | NO | NO | 8.02 | 4.59 | 56.47 | -24.54 | 7.04 |  |  |
| GH1.2020.1.L.002 | MUSCLE | NO | NO | 9.46 | 4.51 | 53.64 | -24.73 | 5.67 |  |  |
| GH1.2020.2.L.002 | MUSCLE | NO | NO | 10.76 | 6.27 | 57.58 | -24.95 | 5.35 |  |  |
| JC1.2020.1.L.001 | MUSCLE | NO | NO | 9.98 | 5.15 | 53.85 | -23.81 | 5.40 |  |  |
| HR1.2020.1.L.002 | MUSCLE | NO | NO | 7.96 | 3.62 | 50.94 | -26.64 | 6.40 |  |  |
| GH1.2020.3.L.001 | MUSCLE | NO | NO | 7.73 | 5.41 | 61.78 | -23.29 | 7.99 |  |  |
| HR1.2020.3.L.001 | MUSCLE | NO | NO | 8.52 | 4.65 | 54.74 | -24.02 | 6.42 |  |  |
| GH1.2020.2.L.001 | MUSCLE | NO | NO | 7.06 | 3.84 | 63.00 | -22.77 | 8.92 |  |  |
| GH1.2020.3.L.003 | MUSCLE | NO | NO | 6.50 | 3.49 | 56.98 | -23.04 | 8.77 |  |  |
| GH1.2020.2.L.002 | MUSCLE | YES | NO | 11.41 | 6.28 | 43.96 | -27.37 | 3.85 | 5.04 | -99.66 |
| HR1.2020.2.L.001 | MUSCLE | YES | NO | 13.14 | 4.13 | 45.38 | -28.30 | 3.45 | 5.58 | -102.14 |
| JC1.2020.2.L.001 | MUSCLE | YES | NO | 14.13 | 4.68 | 45.75 | -28.78 | 3.24 | 4.59 | -97.31 |
| GH1.2020.1.L.002 | MUSCLE | YES | NO | 12.02 | 4.45 | 39.97 | -27.45 | 3.33 | 4.07 | -93.49 |
| HR1.2020.1.L.002 | MUSCLE | YES | NO | 12.68 | 3.83 | 44.58 | -29.40 | 3.52 | 5.39 | -101.02 |
| GH1.2020.1.L.001 | MUSCLE | YES | NO | 13.26 | 5.38 | 44.66 | -27.27 | 3.37 |  |  |
| HR1.2020.3.L.001 | MUSCLE | YES | NO | 13.69 | 5.10 | 45.01 | -26.83 | 3.29 | 5.72 | -107.56 |
| HR1.2020.2.L.002 | MUSCLE | YES | NO | 13.24 | 3.77 | 43.23 | -28.49 | 3.27 | 6.44 | -106.93 |
| GH1.2020.1.L.002 | MUSCLE | YES | YES | 12.90 | 4.38 | 44.69 | -27.23 | 3.46 |  |  |
| GH1.2020.3.L.002 | MUSCLE | YES | NO | 12.19 | 4.81 | 43.38 | -27.15 | 3.56 |  |  |
| GH1.2020.1.L.003 | MUSCLE | YES | NO | 13.19 | 4.90 | 43.73 | -27.71 | 3.32 |  |  |
| HR1.2020.1.L.001 | MUSCLE | YES | NO | 12.87 | 4.53 | 39.48 | -28.35 | 3.07 | 6.92 | -107.03 |
| JC1.2020.3.L.001 | MUSCLE | YES | NO | 12.81 | 5.02 | 41.98 | -27.46 | 3.28 | 6.06 | -105.19 |
| GH1.2020.3.L.001 | MUSCLE | YES | NO | 12.05 | 5.02 | 41.52 | -27.06 | 3.45 |  |  |
| JC1.2020.1.L.001 | MUSCLE | YES | NO | 10.66 | 4.90 | 41.60 | -25.63 | 3.90 | 5.40 | -99.26 |
| JC1.2020.1.L.001 | LIPID | NO | NO |  |  | 69.61 | -20.22 |  |  |  |
| HR1.2020.2.L.002 | LIPID | NO | NO |  |  | 74.56 | -24.43 |  | 11.22 | -192.88 |
| HR1.2020.3.L.001 | LIPID | NO | NO |  |  | 71.13 | -21.24 |  | 14.04 | -214.00 |
| JC1.2020.3.L.001 | LIPID | NO | NO |  |  | 73.12 | -20.91 |  | 11.23 | -187.35 |
| GH1.2020.1.L.003 | LIPID | NO | NO |  |  | 71.75 | -22.75 |  |  |  |
| GH1.2020.1.L.002 | LIPID | NO | YES |  |  | 71.30 | -20.44 |  |  |  |
| HR1.2020.1.L.002 | LIPID | NO | NO |  |  | 59.66 | -23.42 |  | 12.38 | -219.11 |
| JC1.2020.2.L.001 | LIPID | NO | NO |  |  | 76.22 | -21.05 |  | 11.89 | -214.24 |
| GH1.2020.2.L.002 | LIPID | NO | NO |  |  | 72.59 | -21.57 |  |  |  |
| HR1.2020.1.L.001 | LIPID | NO | NO |  |  | 58.74 | -22.50 |  | 11.23 | -190.24 |
| GH1.2020.1.L.002 | LIPID | NO | NO |  |  | 68.64 | -20.44 |  |  |  |
| GH1.2020.3.L.001 | LIPID | NO | NO |  |  | 74.04 | -20.40 |  |  |  |
| HR1.2020.2.L.001 | LIPID | NO | NO |  |  | 74.95 | -22.81 |  | 9.65 | -217.78 |
| GH1.2020.1.L.001 | LIPID | NO | NO |  |  | 70.17 | -20.78 |  |  |  |
| GH1.2020.3.L.002 | LIPID | NO | NO |  |  | 73.36 | -21.85 |  |  |  |
| FR1.2021.3.L.002 | MUSCLE | NO | NO | 6.65 | 5.25 | 53.74 | -24.10 | 8.08 |  |  |
| FR1.2021.3.L.002 | MUSCLE | YES | NO | 13.64 | 5.58 | 46.34 | -27.85 | 3.40 | 6.81 | -105.10 |
| FR1.2021.3.L.001 | MUSCLE | NO | NO | 7.70 | 4.07 | 61.85 | -23.14 | 8.03 |  |  |
| FR1.2021.3.L.001 | MUSCLE | YES | NO | 14.36 | 4.36 | 48.75 | -27.45 | 3.39 | 6.03 | -107.77 |
| FR1.2021.1.L.003 | MUSCLE | YES | NO | 13.22 | 5.59 | 46.30 | -28.37 | 3.50 | 6.84 | -97.21 |
| FR1.2021.1.L.003 | MUSCLE | NO | NO | 9.31 | 5.27 | 57.03 | -25.60 | 6.13 |  |  |
| FR1.2021.1.L.002 | MUSCLE | NO | NO | 8.23 | 5.26 | 56.15 | -25.35 | 6.82 |  |  |
| FR1.2021.1.L.002 | MUSCLE | YES | NO | 16.91 | 5.52 | 54.03 | -28.19 | 3.20 | 5.42 | -118.29 |
| JC2.2020.2.L.001 | MUSCLE | YES | NO | 11.85 | 5.24 | 42.68 | -28.54 | 3.60 | 5.65 | -114.77 |
| JC2.2020.2.L.002 | MUSCLE | YES | NO | 12.85 | 4.60 | 48.43 | -27.92 | 3.77 | 6.01 | -118.40 |
| FR1.2021.2.L.002 | MUSCLE | YES | NO | 12.65 | 4.71 | 41.59 | -30.72 | 3.29 | 5.17 | -96.18 |
| FR1.2021.2.L.001 | MUSCLE | YES | NO | 13.52 | 5.47 | 44.17 | -28.91 | 3.27 | 5.48 | -109.54 |
| JC2.2020.1.L.002 | MUSCLE | YES | NO | 13.68 | 4.90 | 45.85 | -27.76 | 3.35 | 7.83 | -110.69 |
| JC2.2020.1.L.003 | MUSCLE | YES | NO | 12.34 | 5.42 | 47.25 | -27.69 | 3.83 | 5.15 | -79.41 |
| HR2.2020.3.L.002 | MUSCLE | YES | NO | 12.21 | 3.23 | 45.34 | -27.46 | 3.71 | 7.32 | -99.41 |
| FR1.2021.1.L.001 | MUSCLE | YES | NO | 13.48 | 4.86 | 48.21 | -28.73 | 3.58 | 6.44 | -108.48 |
| JC2.2020.1.L.001 | MUSCLE | YES | NO | 13.60 | 4.45 | 45.82 | -28.28 | 3.37 | 6.43 | -104.77 |
| HR2.2020.3.L.003 | MUSCLE | YES | NO | 12.55 | 3.75 | 44.84 | -27.93 | 3.57 | 5.58 | -97.66 |
| HR1.2021.3.L.002 | MUSCLE | YES | NO | 14.79 | 5.01 | 50.26 | -27.43 | 3.40 | 5.66 | -124.37 |
| HR1.2021.2.L.001 | MUSCLE | YES | NO | 10.47 | 3.42 | 52.72 | -26.18 | 5.04 |  |  |
| HR1.2021.2.L.002 | MUSCLE | YES | NO | 11.61 | 4.28 | 45.07 | -28.58 | 3.88 | 5.32 | -103.89 |
| HR1.2021.2.L.003 | MUSCLE | YES | NO | 9.74 | 3.95 | 54.64 | -27.20 | 5.61 | 6.84 | -148.27 |
| HR1.2021.3.L.001 | MUSCLE | YES | NO | 11.27 | 4.49 | 48.76 | -25.97 | 4.33 | 6.64 | -134.78 |
| HR1.2021.1.L.002 | MUSCLE | YES | NO | 14.15 | 3.51 | 48.68 | -29.65 | 3.44 |  |  |
| HR1.2021.1.L.001 | MUSCLE | YES | NO | 9.12 | 2.85 | 39.37 | -27.87 | 4.32 |  |  |
| HR1.2021.1.L.003 | MUSCLE | YES | NO | 12.40 | 4.52 | 42.16 | -28.06 | 3.40 |  |  |
| HR2.2020.3.L.003 | MUSCLE | NO | NO | 7.13 | 3.32 | 54.57 | -24.39 | 7.65 |  |  |
| JC2.2020.1.L.001 | MUSCLE | NO | NO | 5.77 | 3.74 | 46.32 | -24.85 | 8.03 |  |  |
| JC2.2020.1.L.003 | MUSCLE | NO | NO | 7.85 | 5.17 | 59.83 | -25.09 | 7.62 |  |  |
| HR2.2020.3.L.002 | MUSCLE | NO | NO | 5.11 | 2.97 | 63.00 | -23.50 | 12.33 |  |  |
| FR1.2021.1.L.001 | MUSCLE | NO | NO | 6.13 | 4.33 | 65.16 | -24.03 | 10.63 |  |  |
| JC2.2020.1.L.002 | MUSCLE | NO | NO | 3.66 | 3.58 | 64.89 | -22.40 | 17.73 |  |  |
| FR1.2021.2.L.001 | MUSCLE | NO | NO | 13.24 | 4.84 | 47.41 | -28.80 | 3.58 |  |  |
| FR1.2021.2.L.002 | MUSCLE | NO | NO | 12.53 | 4.23 | 55.72 | -28.90 | 4.45 |  |  |
| JC2.2020.2.L.002 | MUSCLE | NO | NO | 3.28 | 4.05 | 67.14 | -22.98 | 20.47 |  |  |
| JC2.2020.2.L.001 | MUSCLE | NO | NO | 6.37 | 4.70 | 64.54 | -24.94 | 10.13 |  |  |
| JC2.2020.2.L.003 | MUSCLE | NO | NO | 9.68 | 5.69 | 56.27 | -24.58 | 5.81 | 7.50 | -148.22 |
| HR2.2020.2.L.001 | MUSCLE | NO | NO | 4.93 | 3.64 | 83.60 | -23.18 | 16.96 | 9.53 | -195.85 |
| JC2.2020.3.L.003 | MUSCLE | NO | NO | 5.52 | 3.87 | 63.91 | -21.09 | 11.58 |  |  |
| JC2.2020.3.L.001 | MUSCLE | NO | NO | 2.97 | 2.89 | 68.04 | -22.20 | 22.91 |  |  |
| JC2.2020.3.L.002 | MUSCLE | NO | NO | 7.37 | 4.58 | 57.82 | -24.57 | 7.85 |  |  |
| HR2.2020.2.L.002 | MUSCLE | NO | NO | 3.02 | 3.27 | 70.66 | -23.30 | 23.40 |  |  |
| HR2.2020.1.L.001 | MUSCLE | NO | NO | 8.23 | 4.10 | 55.19 | -25.39 | 6.71 |  |  |
| JC2.2020.2.L.003 | MUSCLE | YES | NO | 12.06 | 6.18 | 39.58 | -27.15 | 3.28 | 5.40 | -102.74 |
| HR2.2020.2.L.001 | MUSCLE | YES | NO | 12.11 | 3.97 | 44.01 | -27.47 | 3.63 | 5.26 | -91.80 |
| JC2.2020.3.L.003 | MUSCLE | YES | NO | 12.96 | 4.06 | 43.07 | -26.43 | 3.32 | 4.19 | -93.01 |
| JC2.2020.3.L.001 | MUSCLE | YES | NO | 12.33 | 4.93 | 43.38 | -27.51 | 3.52 | 5.80 | -97.68 |
| JC2.2020.3.L.002 | MUSCLE | YES | NO | 13.47 | 5.07 | 46.73 | -27.70 | 3.47 | 5.33 | -98.61 |
| HR2.2020.2.L.002 | MUSCLE | YES | NO | 12.36 | 4.13 | 45.02 | -27.91 | 3.64 | 6.02 | -109.63 |
| HR2.2020.1.L.001 | MUSCLE | YES | NO | 13.44 | 4.44 | 44.84 | -28.51 | 3.34 | 4.99 | -99.11 |
| JC2.2020.2.L.003 | LIPID | NO | NO |  |  | 69.19 | -22.11 |  | 11.86 | -213.13 |
| HR2.2020.2.L.001 | LIPID | NO | NO |  |  | 76.30 | -22.10 |  | 11.10 | -228.64 |
| JC2.2020.3.L.003 | LIPID | NO | NO |  |  |  | -19.28 |  | 13.00 | -225.91 |
| JC2.2020.3.L.001 | LIPID | NO | NO |  |  | 70.00 | -21.58 |  | 14.17 | -220.30 |
| JC2.2020.3.L.002 | LIPID | NO | NO |  |  | 70.25 | -22.58 |  | 13.30 | -207.66 |
| HR2.2020.2.L.002 | LIPID | NO | NO |  |  | 69.49 | -22.60 |  | 12.30 | -211.80 |
| HR2.2020.1.L.001 | LIPID | NO | NO |  |  | 67.06 | -22.72 |  | 11.20 | -186.84 |
| JC2.2020.2.L.001 | LIPID | NO | NO |  |  | 75.85 | -23.05 |  | 12.43 | -228.57 |
| JC2.2020.2.L.002 | LIPID | NO | NO |  |  | 79.74 | -22.11 |  |  |  |
| FR1.2021.2.L.002 | LIPID | NO | NO |  |  | 60.51 | -27.34 |  |  |  |
| FR1.2021.2.L.001 | LIPID | NO | NO |  |  | 55.77 | -28.18 |  |  |  |
| JC2.2020.1.L.002 | LIPID | NO | NO |  |  | 77.14 | -20.98 |  | 10.81 | -229.26 |
| JC2.2020.1.L.003 | LIPID | NO | NO |  |  | 86.88 | -23.14 |  | 12.75 | -225.78 |
| HR2.2020.3.L.002 | LIPID | NO | NO |  |  | 72.06 | -22.02 |  | 13.21 | -230.34 |
| FR1.2021.1.L.001 | LIPID | NO | NO |  |  | 84.23 | -21.94 |  | 12.57 | -231.03 |
| JC2.2020.1.L.001 | LIPID | NO | NO |  |  | 67.68 | -22.57 |  | 13.36 | -211.31 |
| HR2.2020.3.L.003 | LIPID | NO | NO |  |  | 73.09 | -21.81 |  | 10.52 | -217.80 |
| HR1.2021.1.L.003 | LIPID | NO | NO |  |  | 76.80 | -24.16 |  | 13.51 | -220.96 |
| HR1.2021.1.L.001 | LIPID | NO | NO |  |  | 69.24 | -23.61 |  |  |  |
| HR1.2021.1.L.002 | LIPID | NO | NO |  |  | 39.68 | -29.45 |  |  |  |
| HR1.2021.3.L.001 | LIPID | NO | NO |  |  |  | -23.35 |  |  |  |
| HR1.2021.2.L.003 | LIPID | NO | NO |  |  | 72.42 | -24.16 |  |  |  |
| HR1.2021.2.L.002 | LIPID | NO | NO |  |  | 51.51 | -28.63 |  |  |  |
| HR1.2021.2.L.001 | LIPID | NO | NO |  |  | 76.72 | -21.34 |  | 9.65 | -217.78 |
| HR1.2021.3.L.002 | LIPID | NO | NO |  |  | 53.24 | -28.44 |  |  |  |
| FR1.2021.3.L.002 | LIPID | NO | NO |  |  | 66.21 | -21.98 |  |  |  |
| FR1.2021.3.L.001 | LIPID | NO | NO |  |  | 72.10 | -20.30 |  | 11.98 | -190.84 |
| FR1.2021.1.L.003 | LIPID | NO | NO |  |  | 80.11 | -22.06 |  |  |  |
| FR1.2021.1.L.002 | LIPID | NO | NO |  |  | 53.05 | -23.43 |  |  |  |
| PR1.2021.1.L.001 | MUSCLE | NO | NO | 2.86 | 3.24 | 69.43 | -20.55 | 24.28 |  |  |
| PR1.2021.1.L.002 | MUSCLE | NO | NO | 8.85 | 5.06 | 54.74 | -22.83 | 6.19 |  |  |
| PR1.2021.1.L.003 | MUSCLE | NO | NO | 5.88 | 4.24 | 61.54 | -21.38 | 10.47 |  |  |
| PR1.2021.1.L.004 | MUSCLE | NO | NO | 4.29 | 4.50 | 68.49 | -21.12 | 15.98 |  |  |
| PR1.2021.1.L.005 | MUSCLE | NO | NO | 4.54 | 5.25 | 65.39 | -21.53 | 14.40 |  |  |
| PR1.2021.1.L.001 | MUSCLE | YES | NO | 12.55 | 4.68 | 43.62 | -25.74 | 3.48 | 6.71 | -118.43 |
| PR1.2021.1.L.002 | MUSCLE | YES | NO | 13.00 | 5.58 | 41.42 | -25.56 | 3.19 |  |  |
| PR1.2021.1.L.003 | MUSCLE | YES | NO | 13.22 | 4.58 | 42.16 | -26.40 | 3.19 | 5.85 | -114.38 |
| PR1.2021.1.L.004 | MUSCLE | YES | NO | 13.60 | 5.10 | 44.74 | -26.34 | 3.29 | 5.65 | -94.82 |
| PR1.2021.1.L.005 | MUSCLE | YES | NO | 12.70 | 5.55 | 40.61 | -25.76 | 3.20 | 6.09 | -111.92 |
| FB1.2021.1.L.001 | MUSCLE | NO | NO | 11.00 | 11.78 | 46.83 | -18.43 | 4.26 | 6.94 | -131.61 |
| FB1.2021.1.L.002 | MUSCLE | NO | NO | 14.39 | 12.43 | 49.27 | -18.27 | 3.42 | 5.43 | -102.10 |
| DC1.2021.1.L.001 | MUSCLE | NO | NO | 7.04 | 12.06 | 24.14 | -18.02 | 3.43 | 9.36 | -105.28 |
| DC1.2021.1.L.002 | MUSCLE | NO | NO | 15.00 | 11.99 | 49.46 | -17.41 | 3.30 | 8.88 | -92.41 |
| DC1.2021.1.L.002 | MUSCLE | YES | NO | 13.59 | 11.89 | 43.03 | -17.47 | 3.17 | 2.51 | -52.10 |
| DC1.2021.1.L.001 | MUSCLE | YES | NO | 13.32 | 12.56 | 42.75 | -17.40 | 3.21 | 5.65 | -77.02 |
| FB1.2021.1.L.002 | MUSCLE | YES | NO | 15.41 | 13.04 | 48.28 | -17.73 | 3.13 | 5.87 | -60.66 |
| FB1.2021.1.L.001 | MUSCLE | YES | NO | 14.40 | 12.60 | 44.81 | -17.44 | 3.11 | 6.28 | -53.32 |
| PB1.2021.1.L.001 | MUSCLE | NO | NO | 10.97 | 12.76 | 49.67 | -19.07 | 4.53 |  |  |
| PB1.2021.1.L.002 | MUSCLE | NO | NO | 13.43 | 13.05 | 47.35 | -17.63 | 3.52 |  |  |
| PB1.2021.1.L.003 | MUSCLE | NO | NO | 12.01 | 12.28 | 56.52 | -18.33 | 4.71 |  |  |
| PB1.2021.1.L.004 | MUSCLE | NO | NO | 11.29 | 11.60 | 48.81 | -18.43 | 4.32 |  |  |
| PB1.2021.1.L.005 | MUSCLE | NO | NO | 11.67 | 13.41 | 53.17 | -18.12 | 4.56 |  |  |
| PB1.2021.1.L.006 | MUSCLE | NO | NO | 13.93 | 12.63 | 46.68 | -17.16 | 3.35 |  |  |
| PB1.2021.1.L.007 | MUSCLE | NO | NO | 10.12 | 12.48 | 52.61 | -18.63 | 5.20 |  |  |
| PB1.2021.1.L.008 | MUSCLE | NO | NO | 7.75 | 13.97 | 57.11 | -19.57 | 7.37 |  |  |
| PB1.2021.1.L.009 | MUSCLE | NO | NO | 14.41 | 13.08 | 48.97 | -17.01 | 3.40 |  |  |
| HD1.2021.1.L.001 | MUSCLE | NO | NO | 16.02 | 11.84 | 54.69 | -17.47 | 3.41 | 5.81 | -88.58 |
| HD1.2021.1.L.002 | MUSCLE | NO | NO | 8.84 | 12.94 | 44.61 | -18.08 | 5.05 |  |  |
| HD1.2021.1.L.003 | MUSCLE | NO | NO | 9.41 | 11.78 | 41.38 | -17.80 | 4.40 |  |  |
| HD1.2021.1.L.004 | MUSCLE | NO | NO | 12.55 | 12.93 | 51.38 | -17.96 | 4.09 |  |  |
| HD1.2021.1.L.005 | MUSCLE | NO | NO | 11.37 | 14.21 | 55.58 | -18.03 | 4.89 |  |  |
| HR2.2021.1.L.001 | LIPID | NO | NO |  |  | 286.37 | -21.35 |  |  |  |
| HR2.2021.1.L.002 | LIPID | NO | NO |  |  | 162.63 | -24.10 |  |  |  |
| FR2.2021.3.L.001 | LIPID | NO | NO |  |  | 74.27 | -21.25 |  | 10.13 | -220.22 |
| FR2.2021.2.L.003 | LIPID | NO | NO |  |  | 76.29 | -22.16 |  |  |  |
| FR2.2021.1.L.003 | LIPID | NO | NO |  |  | 79.09 | -22.27 |  |  |  |
| DC1.2021.1.L.001 | LIPID | NO | NO |  |  | 41.53 | -21.53 |  | 6.25 | -217.75 |
| FB1.2021.1.L.002 | LIPID | NO | NO |  |  | 46.58 | -22.18 |  | 6.50 | -208.73 |
| HR2.2021.2.L.002 | LIPID | NO | NO |  |  | 82.89 | -22.22 |  | 11.55 | -218.58 |
| FR2.2021.3.L.003 | LIPID | NO | NO |  |  | 76.00 | -25.93 |  | 11.42 | -177.31 |
| FR2.2021.2.L.001 | LIPID | NO | NO |  |  | 61.19 | -23.40 |  | 8.53 | -212.29 |
| FR2.2021.1.L.001 | LIPID | NO | NO |  |  | 73.60 | -22.20 |  | 9.41 | -227.10 |
| FR2.2021.3.L.002 | LIPID | NO | NO |  |  | 63.67 | -27.49 |  | 10.17 | -167.80 |
| FR2.2021.2.L.002 | LIPID | NO | NO |  |  | 86.76 | -23.55 |  | 11.46 | -185.35 |
| FR2.2021.1.L.002 | LIPID | NO | NO |  |  | 67.73 | -20.99 |  | 11.47 | -216.34 |
| HR2.2021.3.L.003 | LIPID | NO | NO |  |  | 70.03 | -22.35 |  | 10.67 | -215.88 |
| HR2.2021.3.L.001 | LIPID | NO | NO |  |  | 70.85 | -21.12 |  | 10.02 | -216.91 |
| HR2.2021.2.L.003 | LIPID | NO | NO |  |  | 66.68 | -23.91 |  | 11.18 | -221.23 |
| HR2.2021.2.L.001 | LIPID | NO | NO |  |  | 77.55 | -27.10 |  | 11.71 | -217.91 |
| HR2.2021.2.L.004 | LIPID | NO | NO |  |  | 73.43 | -25.74 |  | 12.37 | -170.20 |
| HR2.2021.3.L.002 | LIPID | NO | NO |  |  | 60.42 | -23.29 |  | 10.52 | -197.98 |
| PR1.2021.1.L.001 | LIPID | NO | NO |  |  | 75.38 | -19.67 |  | 12.31 | -224.90 |
| PR1.2021.1.L.005 | LIPID | NO | NO |  |  | 70.57 | -20.59 |  | 12.00 | -237.88 |
| PR1.2021.1.L.003 | LIPID | NO | NO |  |  | 69.25 | -19.70 |  | 11.69 | -190.55 |
| PR1.2021.1.L.004 | LIPID | NO | NO |  |  | 73.31 | -20.19 |  | 10.92 | -206.26 |
| PR1.2021.1.L.002 | LIPID | NO | NO |  |  | 58.20 | -21.22 |  | 10.17 | -200.15 |
| HR2.2021.1.L.001 | MUSCLE | NO | NO | 6.39 | 3.177 | 59.6131 | -24.27 | 9.32 |  |  |
| HR2.2021.1.L.002 | MUSCLE | NO | NO | 4.92 | 3.348 | 67.1232 | -25.15 | 13.65 |  |  |
| HR2.2021.2.L.001 | MUSCLE | NO | NO | 5.42 | 3.142 | 65.0281 | -27.33 | 12.01 |  |  |
| HR2.2021.2.L.002 | MUSCLE | NO | NO | 3.87 | 2.906 | 68.8898 | -23.49 | 17.82 |  |  |
| HR2.2021.2.L.003 | MUSCLE | NO | NO | 5.16 | 3.161 | 62.8014 | -25.14 | 12.18 |  |  |
| HR2.2021.2.L.004 | MUSCLE | NO | NO | 9.74 | 3.969 | 50.1705 | -26.96 | 5.15 |  |  |
| HR2.2021.3.L.001 | MUSCLE | NO | NO | 4.51 | 2.822 | 63.4554 | -22.60 | 14.07 |  |  |
| HR2.2021.3.L.002 | MUSCLE | NO | NO | 9.53 | 4.025 | 54.6757 | -26.54 | 5.74 |  |  |
| HR2.2021.3.L.003 | MUSCLE | NO | NO | 6.71 | 3.776 | 56.9578 | -24.32 | 8.49 |  |  |
| FR2.2021.1.L.001 | MUSCLE | NO | NO | 2.14 | 2.909 | 69.601 | -22.64 | 32.47 |  |  |
| FR2.2021.1.L.002 | MUSCLE | NO | NO | 7.44 | 6.241 | 60.8569 | -23.54 | 8.18 |  |  |
| FR2.2021.1.L.003 | MUSCLE | NO | NO | 5.33 | 4.467 | 63.6821 | -23.74 | 11.95 |  |  |
| FR2.2021.2.L.001 | MUSCLE | NO | NO | 6.47 | 3.839 | 58.101 | -24.68 | 8.98 |  |  |
| FR2.2021.2.L.002 | MUSCLE | NO | NO | 11.10 | 4.587 | 52.29 | -26.29 | 4.71 |  |  |
| FR2.2021.2.L.003 | MUSCLE | NO | NO | 5.98 | 4.178 | 64.6203 | -23.81 | 10.80 |  |  |
| FR2.2021.3.L.001 | MUSCLE | NO | NO | 7.05 | 4.717 | 68.1967 | -23.37 | 9.67 |  |  |
| FR2.2021.3.L.002 | MUSCLE | NO | NO | 13.35 | 5.117 | 48.6184 | -28.17 | 3.64 |  |  |
| FR2.2021.3.L.003 | MUSCLE | NO | NO | 13.53 | 6.127 | 54.5919 | -27.05 | 4.04 |  |  |
| HR2.2021.1.L.001 | MUSCLE | YES | NO | 13.59 | 3.484 | 44.6791 | -29.39 | 3.29 | 6.31 | -108.62 |
| HR2.2021.1.L.002 | MUSCLE | YES | NO | 14.52 | 4.56 | 47.6143 | -28.67 | 3.28 | 5.53 | -95.67 |
| HR2.2021.2.L.001 | MUSCLE | YES | NO |  |  |  |  |  | 5.19 | -105.91 |
| HR2.2021.2.L.002 | MUSCLE | YES | NO | 14.02 | 4.073 | 47.8246 | -30.23 | 3.41 | 5.50 | -102.55 |
| HR2.2021.2.L.003 | MUSCLE | YES | NO | 12.49 | 3.992 | 48.3058 | -28.43 | 3.87 | 5.75 | -86.78 |
| HR2.2021.2.L.004 | MUSCLE | YES | NO | 13.76 | 4.618 | 46.0159 | -28.88 | 3.34 | 5.70 | -77.03 |
| HR2.2021.3.L.001 | MUSCLE | YES | NO | 13.52 | 4.071 | 48.6529 | -27.21 | 3.60 | 6.08 | -107.31 |
| HR2.2021.3.L.002 | MUSCLE | YES | NO | 12.80 | 4.1 | 46.5524 | -29.69 | 3.64 | 5.58 | -90.51 |
| HR2.2021.3.L.003 | MUSCLE | YES | NO | 14.77 | 4.571 | 48.7735 | -27.81 | 3.30 | 5.92 | -94.06 |
| FR2.2021.1.L.001 | MUSCLE | YES | NO | 11.89 | 5.039 | 44.7997 | -28.16 | 3.77 | 6.38 | -68.03 |
| FR2.2021.1.L.002 | MUSCLE | YES | NO | 12.31 | 6.581 | 41.9603 | -28.26 | 3.41 | 5.29 | -84.69 |
| FR2.2021.1.L.003 | MUSCLE | YES | NO | 12.28 | 5.387 | 46.1514 | -27.99 | 3.76 | 5.76 | -80.35 |
| FR2.2021.2.L.001 | MUSCLE | YES | NO | 13.47 | 5.075 | 44.7993 | -27.83 | 3.33 | 5.82 | -92.88 |
| FR2.2021.2.L.002 | MUSCLE | YES | NO | 11.76 | 4.938 | 44.2621 | -28.03 | 3.76 | 5.93 | -99.06 |
| FR2.2021.2.L.003 | MUSCLE | YES | NO | 13.12 | 4.956 | 43.2234 | -28.99 | 3.29 | 6.05 | -99.94 |
| FR2.2021.3.L.001 | MUSCLE | YES | NO | 14.60 | 5.734 | 48.5238 | -28.44 | 3.32 | 5.62 | -101.32 |
| FR2.2021.3.L.002 | MUSCLE | YES | NO | 13.40 | 5.333 | 44.4998 | -28.95 | 3.32 | 5.31 | -100.24 |
| FR2.2021.3.L.003 | MUSCLE | YES | NO | 13.37 | 6.558 | 44.093 | -28.42 | 3.30 | 5.49 | -96.96 |
