# Supplementary material for: Are lipids always depleted? Comparison of hydrogen, carbon, and nitrogen isotopic values in the muscle and lipid of larval lampreys
Source: PLoS One. 2024 Jan 11;19(1):e0286535. doi: 10.1371/journal.pone.0286535 (PMC10783746; doi:10.1371/journal.pone.0286535)
Supplement: Attachment — Submitted filename: PONE-D-22-27112_reviewer comments.pdf [file pone.0286535.s004.pdf]

PLOS ONE

Are lipids always light? Lipids in larval lampreys are high in 13C but low in 2H relative to muscle

--Manuscript Draft--

**Manuscript Number:**	PONE-D-22-27112
**Article Type:**	Research Article
**Full Title:**	Are lipids always light? Lipids in larval lampreys are high in 13C but low in 2H relative to muscle
**Short Title:**	Unusual lipid stable isotope ratios in larval lampreys
**Corresponding Author:**	Thomas Evans, Ph.D.<br>Cornell University<br>Ithaca, UNITED STATES
**Keywords:**	stable isotope ratios; lipids; C:N ratios; deuterium; carbon; ammocoete; larval lampreys
**Abstract:**	Stable isotope ratios in organisms can be used to estimate source contributions, but lipids must first be accounted for to interpret these values meaningfully. This is because during lipid synthesis light isotopes of carbon (12C) and hydrogen (1H) are preferentially incorporated into the lipids and as a result lipids are depleted in heavy isotopes. Prior work in lampreys has noted unusual lipid effects, which suggest lipids are enriched in heavy isotopes of carbon (13C), but still depleted in heavy hydrogen (deuterium; 2H). Isotopes ratios of δ2H, δ13C, δ15N were measured from muscle of four lamprey species before and after lipid extraction. In addition, least brook lamprey (Lampetra aepyptera) were collected every three months for a year from two streams in Maryland and the isotope ratios of muscle before and after lipid extraction, and the extracted lipid were measured. The difference between muscle samples before and after lipid extraction (Δδ2H, Δδ13C, Δδ15N) was positively related to lipid proxy (%H or C:N ratio) and were fit best by linear models in Δδ2H and Δδ15N, but a non-linear model for Δδ13C. Unexpectedly, the measured difference between lipid-extracted muscle and lipid δ13C (ΔMLδ13C) was different between months (ANOVA, $F_{3,53}$ = 5.05, p < 0.005) and was always negative. Our work suggests that while lipids are often depleted in 13C, this is not a universal rule; however, the depletion of 2H in lipid synthesis appears broadly true. The physiological mechanism(s) for generating heavy carbon-backbones in larval lamprey lipids remains unknown and requires exploration.
**Order of Authors:**	Thomas Evans, Ph.D.
	Shale Beharie
**Additional Information:**	
**Question**	**Response**
**Financial Disclosure**<br><br>Enter a financial disclosure statement that describes the sources of funding for the work included in this submission. Review the submission guidelines for detailed requirements. View published research articles from *PLOS ONE* for specific examples.<br><br>This statement is required for submission and **will appear in the published article** if the submission is accepted. Please make sure it is accurate.	The author(s) received no specific funding for this work.

Powered by Editorial Manager® and ProduXion Manager® from Aries Systems Corporation

**Unfunded studies**<br>Enter: *The author(s) received no specific funding for this work.*<br><br>**Funded studies**<br>Enter a statement with the following details:<br>• Initials of the authors who received each award<br>• Grant numbers awarded to each author<br>• The full name of each funder<br>• URL of each funder website<br>• Did the sponsors or funders play any role in the study design, data collection and analysis, decision to publish, or preparation of the manuscript?<br>• **NO** - Include this sentence at the end of your statement: *The funders had no role in study design, data collection and analysis, decision to publish, or preparation of the manuscript.*<br>• **YES** - Specify the role(s) played.<br><br>* typeset	
**Competing Interests**<br><br>Use the instructions below to enter a competing interest statement for this submission. On behalf of all authors, disclose any competing interests that could be perceived to bias this work—acknowledging all financial support and any other relevant financial or non-financial competing interests.<br><br>This statement is required for submission and **will appear in the published article** if the submission is accepted. Please make sure it is accurate and that any funding sources listed in your Funding Information later in the submission form are also declared in your Financial Disclosure statement.<br><br>View published research articles from *PLOS ONE* for specific examples.	The authors have declared that no competing interests exist.

**NO authors have competing interests**<br><br>Enter: *The authors have declared that no competing interests exist.*<br><br>**Authors with competing interests**<br><br>Enter competing interest details beginning with this statement:<br><br>*I have read the journal's policy and the authors of this manuscript have the following competing interests: [insert competing interests here]*<br><br>* typeset	
**Ethics Statement**<br><br>Enter an ethics statement for this submission. This statement is required if the study involved:<br><br>• Human participants<br>• Human specimens or tissue<br>• Vertebrate animals or cephalopods<br>• Vertebrate embryos or tissues<br>• Field research<br><br>Write "N/A" if the submission does not require an ethics statement.<br><br>General guidance is provided below. Consult the submission guidelines for detailed instructions. **Make sure that all information entered here is included in the Methods section of the manuscript.**	Animal Research: St. Mary's College of Maryland IACUC, , MS-222 and clove oil to anesthetize before pithing<br><br>Field Research: SCP202090, St. Mary's College of Maryland

Powered by Editorial Manager® and ProduXion Manager® from Aries Systems Corporation

**Format for specific study types**<br><br>**Human Subject Research (involving human participants and/or tissue)**<br>• Give the name of the institutional review board or ethics committee that approved the study<br>• Include the approval number and/or a statement indicating approval of this research<br>• Indicate the form of consent obtained (written/oral) or the reason that consent was not obtained (e.g. the data were analyzed anonymously)<br><br>**Animal Research (involving vertebrate animals, embryos or tissues)**<br>• Provide the name of the Institutional Animal Care and Use Committee (IACUC) or other relevant ethics board that reviewed the study protocol, and indicate whether they approved this research or granted a formal waiver of ethical approval<br>• Include an approval number if one was obtained<br>• If the study involved *non-human primates*, add *additional details* about animal welfare and steps taken to ameliorate suffering<br>• If anesthesia, euthanasia, or any kind of animal sacrifice is part of the study, include briefly which substances and/or methods were applied<br><br>**Field Research**<br><br>Include the following details if this study involves the collection of plant, animal, or other materials from a natural setting:<br>• Field permit number<br>• Name of the institution or relevant body that granted permission	
**Data Availability**<br><br>Authors are required to make all data underlying the findings described fully available, without restriction, and from the time of publication. PLOS allows rare exceptions to address legal and ethical concerns. See the PLOS Data Policy and FAQ for detailed information.	Yes - all data are fully available without restriction

A Data Availability Statement describing where the data can be found is required at submission. Your answers to this question constitute the Data Availability Statement and **will be published in the article**, if accepted.<br><br>**Important:** Stating 'data available on request from the author' is not sufficient. If your data are only available upon request, select 'No' for the first question and explain your exceptional situation in the text box.<br><br>Do the authors confirm that all data underlying the findings described in their manuscript are fully available without restriction?	
**Describe where the data may be found in full sentences. If you are copying our sample text, replace any instances of XXX with the appropriate details.**<br><br>• If the data are **held or will be held in a public repository**, include URLs, accession numbers or DOIs. If this information will only be available after acceptance, indicate this by ticking the box below. For example: *All XXX files are available from the XXX database (accession number(s) XXX, XXX.).*<br>• If the data are all contained **within the manuscript and/or Supporting Information files**, enter the following: *All relevant data are within the manuscript and its Supporting Information files.*<br>• If neither of these applies but you are able to provide **details of access elsewhere**, with or without limitations, please do so. For example:<br><br>*Data cannot be shared publicly because of [XXX]. Data are available from the XXX Institutional Data Access / Ethics Committee (contact via XXX) for researchers who meet the criteria for access to confidential data.*<br><br>*The data underlying the results presented in the study are available from (include the name of the third party*	All relevant data are within the manuscript and its Supporting Information files.

and contact information or URL).<br>• This text is appropriate if the data are owned by a third party and authors do not have permission to share the data.<br><br>* typeset	
Additional data availability information:	

Manuscript

Click here to access/download;Manuscript;Are lipids always depleted 2022_09_28.docx

Full Title: Are lipids always light? Lipids in larval lampreys are high in ^{13}C but low in ^{2}H relative to muscle

Short Title: Unusual lipid stable isotope ratios in larval lampreys

Thomas M Evans[a,*,†], Shale Beharie[a]

[a]St. Mary's College of Maryland, Biology Department, St. Mary's City, MD

*Corresponding author, e-mail: thomas.mark.evans@gmail.com

†Current address: Cornell University, Department of Natural Resources and the Environment, Ithaca, NY

Abstract

Stable isotope ratios in organisms can be used to estimate source contributions, but lipids must first be accounted ~~for~~ to interpret these values meaningfully. This is because during lipid synthesis light isotopes of carbon (^{12}C) and hydrogen (^{1}H) are preferentially incorporated into the lipids and as a result lipids are depleted in heavy isotopes. Prior work in lampreys has noted unusual lipid effects~~, which suggest~~ lipids are enriched in heavy isotopes of carbon (^{13}C)~~, but still depleted in heavy hydrogen (deuterium; ^{2}H)~~. Isotopes ratios of $\delta^{2}H$, $\delta^{13}C$, $\delta^{15}N$ were measured from muscle of four lamprey species before and after lipid extraction. In addition, least brook lamprey (*Lampetra aepyptera*) were collected every three months for a year from two streams in Maryland and the isotope ratios of muscle before and after lipid extraction, and the extracted lipid were measured. The difference between muscle samples before and after lipid extraction ($\Delta\delta^{2}H$, $\Delta\delta^{13}C$, $\Delta\delta^{15}N$) was positively related to lipid proxy (%H or C:N ratio) and were fit best by linear models ~~in~~ $\Delta\delta^{2}H$ and $\Delta\delta^{15}N$, ~~but~~ a non-linear model for $\Delta\delta^{13}C$. ~~Unexpectedly,~~ the measured difference between lipid-extracted muscle and lipid $\delta^{13}C$ ($\Delta_{ML}\delta^{13}C$) was different between months (ANOVA, $F_{3,53} = 5.05$, $p < 0.005$) and was ~~always~~ negative. Our work suggests that while lipids are often depleted in ^{13}C, this is not a universal rule; however, the depletion of ^{2}H in lipid synthesis appears broadly true. The physiological mechanism(s) for generating heavy carbon-backbones in larval lamprey lipids remains unknown and requires exploration.

Introduction

Stable isotope ratios are a powerful tool which have been used to deepen our understanding of numerous biological processes including physiology, nutrient cycling, food web structure, diet, and migration [1]. In natural systems elements generally have ~~only~~ two stable isotopes, a light isotope that is ~~extremely~~ common (>95% of all atoms) and ~~one~~ rare heavier isotope [2]. The ratio of the heavy to the light isotopes varies between organisms and their environment because lighter isotopes are favored during reactions [2,3]. Lighter isotopes have a lower activation energy, and therefore proceed more rapidly when reacting than heavy isotopes [2,4]. The difference between an organism and its starting materials (i.e., discrimination, although sometimes called fractionation [3]) is predictable and has been the subject of much study [5–7].

An organism's stable isotope signature not only reflects materials incorporated from external sources, but also the manipulation of internal sources [8]. For instance, while food sources can be used to build cellular components (e.g., proteins), they can also provide resources to synthesize lipids. Enzymatic synthesis of lipids favors lighter isotopes at a high rate and as a result, lipids are depleted (i.e., they contain lower amounts than the starting materials) in the heavy isotopes of carbon (^{13}C) and hydrogen (^{2}H) and higher amounts of the light isotopes of carbon (^{12}C) [6,9,10] and hydrogen (^{1}H) [11,12], which are the primary atoms in lipids. In contrast, nitrogen is much rarer in lipids [9] and is not altered from dietary sources during lipid synthesis.

The depletion of heavy isotopes of carbon [6] and hydrogen [13] in lipids is well documented and is the result of conserved biochemical pathways [10,11]. Lipids are ubiquitous in living organisms, and researchers using carbon and hydrogen stable isotope ratios need to

account for lipids to make meaningful interpretations [6,14] either by extracting them from a sample or mathematically accounting for them prior to interpretation [6,14–16]. However, measuring the amount of lipids in an organism can increase the cost of a study and the number of samples necessary. ~~Thankfully,~~ stable isotopes of carbon ($\delta^{13}C$) are often measured simultaneously to stable isotopes of nitrogen ($\delta^{15}N$) and the ratio of C:N reported; the C:N ratio increases as carbon is sequestered into lipids and C:N can be used as a proxy for lipids [6,9,17]. As a result, as C:N values increase the $\delta^{13}C$ values in tissues decline (because of the aforementioned reductions in ^{13}C); this phenomenon has been demonstrated in a wide-range of animals [6,9]. However, in numerous studies of larval lampreys authors have reported that as C:N ratios increased $\delta^{13}C$ also increased [18–20].

Lampreys are jawless fishes, which belong to the most basal extant vertebrate group [21], with a complex life cycle including a larval period and a true metamorphosis [22]. Although there are ~40 species of lamprey, all species have a similar larval period. The larval period usually lasts from 3-7 years (although it can be longer) and is used to collect sufficient lipid reserves to fuel metamorphosis [23]. Larval lampreys are primary consumers which feed by collecting detritus (e.g., decaying organic matter and bacteria) [24,25], often primarily allochthonous in origin (i.e., terrestrial) [26,27], and to varying extents algae in stream sediments [23]. Larvae grow slowly, often reaching only a few grams in weight [28], but accumulate large quantities of lipids in their bodies (~15% of their wet weight) [29,30]. However, larval growth is not constant, larvae grow most during the spring and to a lesser extent the fall [31], often declining in condition throughout the summer [29,32]. Larvae eventually undergo a metamorphosis at the end of the summer during which they do not feed, instead they rely on accumulated lipid stores [23,30].

Larval lamprey isotope ratios are often highly variable [18–20,33,34], although many studies have not accounted for lipids [18–20,33]. However, if larval lamprey $\delta^{13}C$ is mathematically corrected for lipids following published equations [6,9], larval lamprey $\delta^{13}C$ become unexplainably high [18], appearing to be more like those of marine predators instead of stream detritivores. Interestingly, although more limited work on larval lampreys using $\delta^{2}H$ has been done [26,34], when lipids were removed $\delta^{2}H$ values have risen [26], as would be expected based on theoretical expectations. Prior work has variably interpreted stable isotopes in larvae, either as larvae consuming a range of food sources but often incorporating large quantities of algae [18,19] or, if lipids were removed, as larvae being almost entirely dependent on allochthonous detritus [26,34]. Larval lamprey lipids may be unusual and contributing to these remarkable signatures, but this needs to be formally established in the literature body through measurements of stable isotope ratios in both the muscle and the lipid.

It is ~~critical~~ to establish if lipid stable isotopes can deviate from expectations to ensure larval lamprey $\delta^{2}H$ and $\delta^{13}C$ are useful in predicting source dependence, but more broadly because lipids are a common concern in stable isotope analysis and lampreys may suggest alternative isotope portioning during lipid synthesis. Additionally, the use of $\delta^{2}H$ in stable isotope ecology is growing, and recommendations for interpreting their values are increasingly important to allow broader use [13]. Based on prior isotope ratio measurements in larval lampreys [18,19], we hypothesized that larval lamprey lipids would be enriched in ^{13}C relative to muscle, and therefore the $\delta^{13}C$ would decline after lipids were extracted. In contrast, we hypothesized that larval lamprey lipids would be depleted in ^{2}H, as expected based on other organisms [11], and the $\delta^{2}H$ in muscle would increase after lipid extraction. Finally, because

there is relatively little nitrogen in lipids, we hypothesized that muscle $\delta^{15}N$ would be unchanged by lipid extraction.

Methods

Larval lamprey samples

Larval least brook lamprey (*Lampetra aepyptera*) were sampled ~~from~~ two rivers (Henderson Run and Furnace Brook) near St. Mary's City, Maryland in September and December of 2020, and then in March and May of 2021 (Fig 1). Larvae were collected along a stream reach of ~100 m by backpack electrofisher, sampling habitat likely to contain larvae (soft sediments and depositional areas) [23]. Larvae were collected alive by backpack electrofisher (LR-24, Smith Root) using two power settings. Larvae were encouraged to leave the sediment with a low-power setting (3 hz, 100 V, 2:2 pulse pattern), and ~~immobilized when they were observed with a high-power setting (30 Hz, 120 V, continuous output) was activated, which immobilized them for collection~~. ~~In addition,~~ sea lamprey larvae were collected once from the Little Patuxent River (Maryland, USA) in May 2021, ~~and~~ least brook lamprey from Greenhill Run in September 2020 (Maryland, USA). Within two hours of collection, larvae were transported in stream water to the ~~lab~~. Larvae were immediately euthanized by overdose in MS-222 and then dissected, or if time was insufficient to process all larvae rapidly, frozen at -20°C until they could be processed later. The collection and handling of these lampreys was approved by St. Mary's College of Maryland institutional animal care and use committee (IACUC).

Muscle samples were placed into a microcentrifuge tube and then dried at 60°C for 24 hours. After drying, a subsample was lipid extracted following a modified Folch method [35]. Briefly, subsamples were washed with ~1 mL of 2:1, by volume, chloroform to methanol three

times. Samples were spun in a centrifuge at 5000 rpm for five minutes between each wash before the supernatant was decanted and more chloroform methanol was added. All supernatants were collected and dried for 24 hours at 60°C. After three washes the muscle was considered lipid free, as the supernatant was clear, and the muscle had become brittle and lighter in color. The container with evaporated supernatant had a viscous yellow to orange liquid, assumed to be lipid.

To increase the number of muscle samples, data from larvae collected from other locations were also used in this study. Least brook lamprey from Clearfork River (Ohio, USA) and American brook lamprey (*Lethenteron appendix*) from the Mad River (Ohio, USA) were collected in 2010. The collection and handling of these lampreys was approved by The Ohio State University IACUC. The $\delta^{13}C$ and $\delta^{15}N$ from these lampreys have already been published, but $\delta^{2}H$ was also measured in these samples and was only included in a thesis [36]. Additionally, American brook lamprey collected in the Genesee River in August 2017 were also used for analysis in this paper. Again, muscle $\delta^{2}H$, $\delta^{13}C$, $\delta^{15}N$ were previously published, but the study only reported on lipid-extracted muscle (or muscle with relatively low C:N values: C:N <4). Lastly, Pacific lamprey (*Entosphenus tridentatus*) larvae from [37], which were tank reared their entire lives in Washington, USA, were also included in the analyses.

The way in which these larvae were collected is reported in full in each study, but ~~for completeness~~ a short summary is provided here. Wild caught larvae were collected using a backpack electrofisher with two shock settings. All larvae were euthanized by overdose in MS-222 or clove oil and then dissected by filleting and skinning lateral muscle, being careful to avoid any organs or the notochord. Muscle samples were dried and stored as for muscle samples collected in Maryland. Lipids were extracted following the Folch method but these studies did not retain the lipids, discarding them ~~instead~~ after all washes. Lipids are rarely measured in

stable isotope studies examining food webs, ~~because they are mostly a nuisance factor which prevent~~ interpretation of trophic linkages; only larval lipid samples from Maryland were measured.

Stable isotope ratios are reported in δ (delta) notation using the equation: $\delta^{x}Y = [(R_{sample}/R_{standard}) - 1] * 1000$, where Y is the element, x is the heavy isotope measured, and R is the ratio of heavy to light isotope in the sample and universal standards. As a result, isotope ratios are reported in per mil (‰) and can be positive or negative. Positive values mean the sample is enriched in the heavy isotope, while negative values mean the sample is depleted in the heavy isotope relative to the standard.

Stable isotope samples for $\delta^{13}C$ and $\delta^{15}N$ were weighed and packed into tin capsules, while those for $\delta^{2}H$ (sometimes denoted as δD) were packed in silver capsules. Lamprey samples collected in Maryland were sent to the Cornell University Stable Isotope Laboratory (COIL). At COIL, samples were analyzed for $\delta^{13}C$ and $\delta^{15}N$ on a Thermo Finnigan Delta Plus and $\delta^{2}H$ samples were analyzed on a Delta V IRMS through a ConFlo III. Standard deviations for 3-4 internal standards (5-8 standard samples for each type per run) were 0.2‰ for $\delta^{13}C$, 0.3‰ for $\delta^{15}N$, ~~or~~ 2‰ for $\delta^{2}H$. Samples from prior studies were either analyzed by COIL on the same instruments or at the University of California (UC) Davis Stable Isotope Facility (SIF) using a PDZ Europa ANCA- GSL (EA) attached to a PDZ Europa 20- 20 isotope ratio mass spectrometer (IRMS). We did not send replicate samples between ~~labs~~ to test the reliability of measurements on different instruments, but internal standards ~~suggest such~~ differences would be small and contribute little, if at all, to interpretive differences.

Statistical Analysis

To confirm the direction and magnitude the effect of lipid extraction has on muscle, the difference between muscle and lipid-extracted muscle was calculated as follows: $\Delta\delta^{2}H = \delta^{2}H_{extracted} - \delta^{2}H_{untreated}$, $\Delta\delta^{13}C = \delta^{13}C_{extracted} - \delta^{13}C_{untreated}$, and $\Delta\delta^{15}N = \delta^{15}N_{extracted} - \delta^{15}N_{untreated}$. We fit the $\Delta\delta^{13}C$ and $\Delta\delta^{15}N$ against the C:N ratio (a common proxy for lipid content [6,9]) with four models, all of which have been used to fit similar data in prior studies [6,9]. Our simplest model was a linear model (fit by the lm function in R):

$$\Delta\delta^{x}Y = a * C{:}N + b, \text{ [eqn. 1]}$$

where b is the y-intercept and therefore -b/a is the estimate of C:N in a lipid-free sample (x-intercept). We also ln-transformed the C:N data [9] (fit by the nls function in R):

$$\Delta\delta^{x}Y = a * \ln(C{:}N) + b, \text{ [eqn. 2]}$$

and as a result, $e^{(-a/b)}$ would be the model estimate of C:N in a lipid-free sample.

We also fit the data with a non-linear model [9] (fit by the nls function in R):

$$\Delta\delta^{x}Y = (a * C{:}N + b) / (C{:}N + c), \text{ [eqn. 3]}$$

where a is an asymptote (i.e., an estimate of the difference between lipid and muscle), -b/a is the estimate of C:N in a lipid-free sample (x-intercept). Lastly, we also fit a simplified version of eqn. 3 (fit by the nls function in R):

$$\Delta\delta^{x}Y = (a * C{:}N + b) / (C{:}N), \text{ [eqn. 4]}$$

where a remains the asymptote and -b/a the estimate of C:N in a lipid-free sample. For these models we pooled all samples. We examined model residuals to test for homoscedasticity and compared models using Akaike Information Criterion (AIC) values using the AIC function in R v4.2.1. If models had similar support based on AIC scores (AIC scores < 4 units different), the simpler model was selected as the top model. Samples from all sites in Maryland, Evans and Lampman and Evans and Weber contributed to this analysis.

For $\Delta\delta^2H$ we used the equations 1, 2, and 4, but we used the percentage of hydrogen (%H) in the untreated sample instead of C:N ratio. We chose to use percent hydrogen because this was measured on each δ^2H sample (C:N was measured on a separate sample submitted for $\delta^{13}C/\delta^{15}N$). The percentage hydrogen in the body should increase with lipids [38] since lipids include numerous hydrogens, the other cellular components (e.g., proteins and carbohydrates) were not expected to increase. Model selection followed the same pattern as described for $\delta^{13}C/\delta^{15}N$. Samples from Maryland (Henderson Creek and Johns Creek), [37] and [26] contributed to this analysis.

We did not include species, location, or date of collection in $\Delta\delta^xY$ models. Larval lampreys all have similar life history and are not expected to synthesize lipids using different biochemical pathways, regardless of species or where they are collected. We anticipated lipid synthesis would be similar year-round because there is little published stable isotope data for larval lamprey outside of the summer growing season.

We assumed that after lipid extraction muscles were lipid-free, so we used a t-test to compare the top Δ^xY model estimate of the percentage hydrogen in a lipid-free sample or C:N in lipid-free sample with those measured in lipid-extracted muscle. We also evaluated the reliability of the top Δ^xY model by calculating the difference between muscle and lipid samples ($\Delta_{ML}\delta^2H = \delta^2H_{extracted} - \delta^2H_{lipid}$ and $\Delta_{ML}\delta^{13}C = \delta^{13}C_{extracted} - \delta^{13}C_{lipid}$) and comparing it to the asymptote (if present). Variation in $\Delta_{ML}\delta^{13}C$ appeared larger in some months than others, so an analysis of variance (ANOVA) with $\Delta_{ML}\delta^{13}C$ as the response and month as a predictor was used; a Tukey honest significant difference test was used to compare contrasts. All analyses were performed in R v4.2.1 [39].

Results

Isotope ratios models

The ~~range of~~ C:N ratios in muscle ~~was large, ranging~~ from 3.4 to 32.5 (Fig 1). Muscle $\delta^{13}C$ was positively related to the C:N ratio, and the relationship ~~often~~ appeared non-linear (Fig 1a). Muscle $\delta^{15}N$ ~~sometimes~~ declined with increasing the C:N ratio but the pattern was not universal, and variance was often high (Fig 1b).

Figure 1. a) The $\delta^{13}C$ and b) the $\delta^{15}N$ of lamprey muscle samples regressed against their C:N ratio, colored by the month in which they were collected. The species collected is denoted by shape (ABL = American brook lamprey, LBL = least brook lamprey, SL = sea lamprey).

Percent hydrogen was positively related to the C:N ratio measured in a separate sample, although variation increased as C:N values increased (Fig 2a). Muscle $\delta^{2}H$ was negatively related to the percentage of hydrogen in the measured sample (Fig 3b).

Figure 2. a) The percent hydrogen in a $\delta^{2}H$ muscle sample regressed against the C:N ratio measured for $\delta^{13}C/\delta^{15}N$ in a replicate. b) The $\delta^{2}H$ muscle sample regressed against the percent hydrogen in the muscle sample. Samples are colored by month of collection, and the species collected is denoted by shape (ABL = American brook lamprey, LBL = least brook lamprey, SL = sea lamprey).

Regressing $\Delta\delta^2H$ against the percent hydrogen resulted in non-linear model having the lowest AIC score, but only one of its three terms was significant (Fig 3a; Table 1). The linear relationship had a slightly higher AIC score (3 units), but all its terms were significant (Fig 3a; Table 1). Therefore, we selected a linear model as the top model for $\Delta\delta^2H$ (eqn. 1) and as a result there was no calculable maximum difference between muscle and lipid. The $\Delta\delta^2H$ top model (eqn. 1) estimated the precent hydrogen in a lipid-free sample would be 6.02% (Table 1) and was not different from the measured value in the quarterly lipid-free samples (6.04%; $T_{20,117} = 0.06$, p-value > 0.5).

Figure 3. **a) The $\Delta\delta^2H$ ($\delta^2H_{extracted} - \delta^2H_{untreated}$) of muscle samples regressed against the percent hydrogen in the unextracted muscle sample.** b) The $\Delta\delta^{13}C$ ($\delta^{13}C_{extracted} - \delta^{13}C_{untreated}$) and the c) $\Delta\delta^{15}N$ ($\delta^{15}N_{extracted} - \delta^{15}N_{untreated}$) of muscle samples regressed against the C:N ratio in the unextracted muscle sample. The lines of best fit are overlain; the solid line is y = ax +b, the dashed line is y = a*ln(x) + b, the dotted line is y = (ax + b)/(x + c), and the dot-dash line is y = (ax + b)/x.

Table 1. The model, equation number used in the present study, their AIC values, number of samples (n), and parameter estimates (mean ± SE).

Isotope Ratio	Model	Eqn. #	AIC	n	a	b	c
δ^2H	$\Delta\delta^2H$ ~ a * %H + b	1	168	22	25.5 ± 2.9***	-154 ± 21***	NA
δ^2H	$\Delta\delta^2H$ ~ a * ln (%H) + b	2	171	22	-347± 47***	-191± 24***	NA
δ^2H	$\Delta\delta^2H$ ~ (a * %H + b) / (%H + c)	3	165	22	-76 ± 56	398 ± 346	-12.7 ± 1.94***
δ^2H	$\Delta\delta^2H$ ~ (a * %H + b) / (%H)	4	174	22	227 ± 27***	-1405 ± 191***	NA
$\delta^{13}C$	$\Delta\delta^{13}C$ ~ a * C:N + b	1	385	121	-0.250 ± 0.022***	-0.607 ± 0.250***	NA
$\delta^{13}C$	$\Delta\delta^{13}C$ ~ a * ln (C:N) + b	2	339	121	2.94 ± 0.36***	-2.87 ± 0.18***	NA
$\delta^{13}C$	$\Delta\delta^{13}C$ ~ (a * C:N + b) / (C:N + c)	3	328	121	-6.79 ± 0.61***	-23.17 ± 1.76***	1.41 ± 1.30***

$\delta^{13}C$	$\Delta\delta^{13}C \sim (a * C{:}N + b) / (C{:}N)$	4	328	121	-6.15 ± 0.23***	-22.05 ± 1.31***	NA
$\delta^{15}N$	$\Delta\delta^{15}N \sim a * C{:}N + b$	1	71	121	0.050 ± 0.0060***	0.064 ± 0.054	NA
$\delta^{15}N$	$\Delta\delta^{15}N \sim a * \ln(C{:}N) + b$	2	87	121	-0.40 ± 0.13***	0.45 ± 0.065***	NA
$\delta^{15}N$	$\Delta\delta^{15}N \sim (a * C{:}N + b) / (C{:}N + c)$	3	129	121	0.46 ± 0.04***	-1.70 ± 0.22***	-3.73 ± 0.37***
$\delta^{15}N$	$\Delta\delta^{15}N \sim (a * C{:}N + b) / (C{:}N)$	4	103	121	0.90 ± 0.091***	-2.71 ± 0.52***	NA

Note: *** denotes p-values < 0.001

Fitting of $\Delta\delta^{13}C$ provided strong support for non-linear models (Fig. 3b), which always had AIC values >40 units different from the linear model (Table 1) and more homoscedastic residuals (data not shown). Equations 3 and 4 had the lowest AIC scores, and all their terms were significant, but Equation 4 was a simpler model and was selected as the top model. The top model for $\Delta\delta^{13}C$ (eqn. 4) estimated $\Delta_{ML}\delta^{13}C$ to be -6.15 ± 0.23‰ (mean ± SE, n= 119; Table 1) which was lower than the mean measured value (-5.03 ± 0.23‰, n=57). The model also overestimated the C:N ratio (3.59 ± 0.001, mean ± SE) of a lipid-free sample ($T_{119,121}$ = 15.72, p-value < 0.0001), in measured samples it was 3.34 ± 0.02 (mean ± SE, n = 121). Visual inspection of the top model for $\Delta\delta^{13}C$ (Fig. 3b) and its residuals (data not shown) showed that it was not entirely homoscedastic, fitting values farther from a C:N of 3.5 better than those close to it.

Lastly, $\Delta\delta^{15}N$ was best fit by a linear model based on AIC scores (Table 1) and homoscedastic residuals (data not shown). The slope of $\Delta\delta^{15}N$ in the linear model (eq. 1) was different from zero (0.050 ± 0.006‰, mean ± SE), but the intercept was not (Fig 3c; Table 1). Visually, as C:N approached seven, $\Delta\delta^{15}N$ appeared to increase, suggesting for low C:N samples, lipids had only a small, or no, influence on $\Delta\delta^{15}N$ (Fig 3c). The data were heavily right skewed, violating assumptions of normality in the models. If instead, the average difference was considered for samples with C:N < 10, mean $\Delta\delta^{15}N$ after lipid extraction was 0.36‰ (SD ± 0.30, n=98); the mean difference of all data (n=121) raised the mean and SD by ~0.1‰.

Seasonal difference between muscle and lipid

Visually there was no evidence that the measured difference between muscle and lipid δ^2H ($\Delta_{ML}\delta^2H$) varied between months (Fig. 4a). An ANOVA confirmed this ($F_{3, 31} = 1.56$, $p > 0.2$); the overall mean difference was 111‰ (SE = ± 21, n = 35; Fig. 4a). In contrast, the $\Delta_{ML}\delta^{13}C$ was different between months (ANOVA, $F_{3,53} = 5.05$, $p < 0.005$); March was different from all months, but no other month was different from another (Fig 4b). The mean $\Delta_{ML}\delta^{13}C$ in March was -3.52‰ (SE = ± 0.67, n = 15) and was lower than other months (mean = -5.58‰, SE = ± 0.20, n = 42; Fig 4b). In March, the variance was also larger (6.8‰) than other months (May = 2.9‰, Sept = 0.87‰, Dec = 0.46‰) and March included samples where the muscle and lipid had identical $\delta^{13}C$ values (Fig 4b). The top model for $\Delta\delta^{13}C$ (eqn. 4) was lower than the $\Delta_{ML}\delta^{13}C$ measured value in March (-3.52 ±0.67‰, n= 15) or all other months (-5.58 ±0.20‰, n = 42).

Figure 4. a) The $\Delta_{ML}\delta^2H$ ($\delta^2H_{untreated} - \delta^2H_{lipid}$) and b) the $\Delta_{ML}\delta^{13}C$ ($\delta^{13}C_{untreated} - \delta^{13}C_{lipid}$) between muscle and lipid samples if measured in the same individual plotted by the month of collection and color for their origin.

Discussion

The carbon backbone of larval lamprey lipids is often enriched in ^{13}C relative to muscle, but the attached hydrogens are depleted in the heavy isotope (^2H; and therefore, enriched in the lighter isotope ^1H). It is unclear why lipid synthesis should prefer a lighter isotope in one case (hydrogen), but not another (carbon). Larval lampreys are known to have unusual lipid origins, relying on muscle instead of the liver cells for synthesis [29]. Alternatively, larval lampreys may

retain lipid scaffolding from other organisms they feed on, such as bacteria and algae [40], and then saturate any double bonds that occur primarily with ^{1}H. Stream algae $\delta^{13}C$ is variable and dependent on water velocity [41] and their lipids may be relatively enriched in $\delta^{13}C$ compared to larval lamprey muscle.

Additionally, larval lampreys have low metabolisms, sometimes requiring a decade or more to grow only a few grams [23]. Larval lampreys consume only a small fraction of lignin or cellulose in their diet (<10%) [42], ensuring much of the material they consume passes undigested through the gut; some ingested algae pass through larval lamprey guts and emerge alive [42,43]. Presumably when feeding, larval lampreys quickly consume the few carbohydrates they encounter, and then rely upon energy from amino acids and lipids for most metabolic costs. Only after quickly utilizing the relatively fast reacting ^{12}C and excreting it as $^{12}CO_2$ or incorporating it into their own amino acids, would larvae begin to synthesize lipids. Since ^{12}C is expelled or sequestered the body fatty acids may be relatively enriched in ^{13}C.

The seasonal difference in carbon between muscle and lipid ($\Delta_{ML}\delta^{13}C$; Fig 4b) suggests the enrichment in ^{13}C occurs primarily after the spring algal bloom and may be related to a physiological shift in the larvae. Enriched ^{13}C lipids appear to exist year-round in some larvae, but during spring months some individuals have lipids which can be produced with $\delta^{13}C$ almost identical to muscle (Fig 4b). As the year proceeds these lipids appear to be reworked and enriched with ^{13}C (Fig 4b). Larval lampreys deposit the greatest amount of lipids during periods of high diatom density [31]. Therefore, larval lipid isotopic signatures may partially be the result of their low metabolism and the manipulation of lipids during prolonged periods of slow or negative growth.

We found lipid extraction increased $\delta^{15}N$ (Fig 3c), and our value (~0.4‰) was close to the value (0.3‰) reported in a metanalysis of lipid extraction impacts on $\delta^{15}N$ [44]. Removal of lipids relies on solvents, and these solvents are likely to remove proteins that are lipophilic, which could alter $\delta^{15}N$ [45]. Our work reinforces findings from prior work that researchers should measure $\delta^{15}N$ prior to lipid extraction [44]. The increase we observed in lamprey $\delta^{15}N$ as C:N ratios increased (Fig. 3c), suggest that either: 1) $\delta^{15}N$ is also manipulated in the body (albeit to a lesser extent than $\delta^{2}H$ or $\delta^{13}C$) or 2) larval lampreys with higher lipid stores were feeding on isotopically different food sources than those with lower lipid stores.

The use of $\delta^{2}H$ is an emerging field [13], and lipids are known to alter bulk isotope ratios [11,12], but the work in this study underscores the strong effects lipids can have on measures of $\delta^{2}H$ (Fig. 2). The C:N ratio has often been used to correct for lipids in samples [6,9], but researchers measuring $\delta^{2}H$ may not have access to C:N values. Even if C:N ratios are available, $\delta^{2}H$ and C:N ratios are often measured in a different subsample, as in the present study, because fewer isotope facilities can measure $\delta^{2}H/\delta^{13}C/\delta^{15}N$ simultaneously and the cost often increases for such an analysis. As a result, the C:N ratio of the $\delta^{2}H$ sample is often unknown, albeit it is likely similar to the value reported when measuring $\delta^{13}C/\delta^{15}N$ because the samples were homogenized. However, it is challenging to ensure samples remain truly homogeneous given that melting point of lipids is near room temperature and lipids tend to separate from the protein component (T. M. Evans, personal observation); the increased variance seen as C:N ratios increased (Fig. 2a) is likely a result of incomplete homogenization between samples. Additionally, $\delta^{2}H$ samples are small (<0.4 mg), making them vulnerable to small changes in the percentage of lipid within a sample. We utilized the percentage of hydrogen in a sample (Fig 2b), which provided better fits than C:N values (Fig 3), and presumably better estimates of the effect

of lipid on samples. As has been done for $\delta^{13}C$ [6,9,15], to increase the efficacy of $\delta^{2}H$, a comprehensive examination across multiple groups correlating the percentage of hydrogen with the percentage of lipid and their effect on the isotope ratio is needed.

Our work suggests, that while lipids are often depleted in ^{13}C relative to muscle [6], this is not a universal rule. However, larval lampreys follow expectations for hydrogen [11,12], where lipids are depleted in the heavier isotope (^{2}H) relative to the muscle. Scientists working with isotopes in larval lampreys should anticipate lipid effects. To ensure they interpret their stable isotope ratios meaningfully, larval lamprey samples should be run in duplicate before and after lipid extraction, as mathematical correction appears to depend on the season. These findings should not be assumed to be true of juvenile and adult lamprey lipids. The physiological mechanism(s) to explain larval lamprey lipids is unknown but is necessary to fully explain this phenomenon. Such work will provide deeper insights into how isotopes in animal bodies can be manipulated.

Acknowledgements

We thank St. Mary's College of Maryland for providing support and sampling equipment and Dr. Jeffrey Lombardo and three anonymous reviewers for reading and making comments on an earlier draft.

References

1. Michener RH, Lajtha K. Stable Isotopes in Ecology and Environmental Science. 2nd ed. Cambridge, MA: Blackwell Scientific Publications; 2007.

2. Fry B. Stable Isotope Ecology. Springer; 2006.

3. Ben-David M, Flaherty EA. Stable isotopes in mammalian research: a beginner's guide. Journal of Mammalogy. 2012;93: 312–328. doi:10.1644/11-MAMM-S-166.1

4. Dawson TE, Mambelli S, Plamboeck AH, Templer PH, Tu KP. Stable Isotopes in Plant Ecology. Annual Review of Ecology and Systematics. 2002;33: 507–559. doi:10.1146/annurev.ecolsys.33.020602.095451

5. Post DM. Using stable isotopes to estimate trophic position: Models, methods, and assumptions. Ecology. 2002;83: 703–718.

6. Post DM, Layman CA, Arrington DA, Takimoto G, Quattrochi J, Montaña CG. Getting to the fat of the matter: models, methods and assumptions for dealing with lipids in stable isotope analyses. Oecologia. 2007;152: 179–189.

7. Stephens RB, Ouimette AP, Hobbie EA, Rowe RJ. Reevaluating trophic discrimination factors ($\Delta\delta^{13}C$ and $\Delta\delta^{15}N$) for diet reconstruction. Ecological Monographs. 2022;n/a: e1525. doi:10.1002/ecm.1525

8. Quinby BM, Creighton JC, Flaherty EA. Stable isotope ecology in insects: a review. Ecological Entomology. 2020;45: 1231–1246. doi:10.1111/een.12934

9. Logan JM, Jardine TD, Miller TJ, Bunn SE, Cunjak RA, Lutcavage ME. Lipid Corrections in Carbon and Nitrogen Stable Isotope Analyses: Comparison of Chemical Extraction and Modelling Methods. Journal of Animal Ecology. 2008;77: 838–846.

10. DeNiro M, Epstein S. Mechanism of carbon isotope fractionation associated with lipid synthesis. Science. 1977;197: 261–263. doi:10.1126/science.327543

11. Sessions AL, Burgoyne TW, Schimmelmann A, Hayes JM. Fractionation of hydrogen isotopes in lipid biosynthesis. Organic Geochemistry. 1999;30: 1193–1200. doi:10.1016/S0146-6380(99)00094-7

12. Pilecky M, Winter K, Wassenaar LI, Kainz MJ. Compound-specific stable hydrogen isotope (δ^2H) analyses of fatty acids: A new method and perspectives for trophic and movement ecology. Rapid Communications in Mass Spectrometry. 2021;35: e9135. doi:10.1002/rcm.9135

13. Vander Zanden HB, Soto DX, Bowen GJ, Hobson KA. Expanding the Isotopic Toolbox: Applications of Hydrogen and Oxygen Stable Isotope Ratios to Food Web Studies. Frontiers in Ecology and Evolution. 2016;4. doi:10.3389/fevo.2016.00020

14. Wilkinson GM, Cole JJ, Pace ML. Deuterium as a food source tracer: Sensitivity to environmental water, lipid content, and hydrogen exchange. Limnology and Oceanography: Methods. 2015;13: 213–223. doi:10.1002/lom3.10019

15. Sweeting CJ, Polunin NVC, Jennings S. Effects of chemical lipid extraction and arithmetic lipid correction on stable isotope ratios of fish tissues. Rapid Commun Mass Spectrom. 2006;20: 595–601. doi:10.1002/rcm.2347

16. Kiljunen M, Grey J, Sinisalo T, Harrod C, Immonen H, Jones RI. A revised model for lipid-normalizing $\delta^{13}C$ values from aquatic organisms, with implications for isotope mixing models. J Appl Ecol. 2006;43: 1213–1222. doi:10.1111/j.1365-2664.2006.01224.x

17. Hoffman JC, Sierszen ME, Cotter AM. Fish tissue lipid-C:N relationships for correcting $\delta^{13}C$ values and estimating lipid content in aquatic food-web studies: Fish tissue lipid-C:N relationships. Rapid Commun Mass Spectrom. 2015;29: 2069–2077. doi:10.1002/rcm.7367

18. Evans TM, Bauer JE. Identification of the nutritional resources of larval sea lamprey in two Great Lakes tributaries using stable isotopes. Journal of Great Lakes Research. 2016;42: 99–107. doi:10.1016/j.jglr.2015.11.010

19. Evans TM, Bauer JE. Using stable isotopes and C:N ratios to examine the life-history strategies and nutritional sources of larval lampreys. J Fish Biol. 2016;88: 638–654. doi:10.1111/jfb.12858

20. Dias E, Miranda ML, Sousa R, Antunes C. Riparian vegetation subsidizes sea lamprey ammocoetes in a nursery area. Aquat Sci. 2019;81: 44. doi:10.1007/s00027-019-0641-4

21. Heimberg AM, Cowper-Sal·lari R, Sémon M, Donoghue PCJ, Peterson KJ. microRNAs reveal the interrelationships of hagfish, lampreys, and gnathostomes and the nature of the ancestral vertebrate. PNAS. 2010;107: 19379–19383. doi:10.1073/pnas.1010350107

22. Evans TM, Janvier P, Docker MF. The evolution of lamprey (Petromyzontida) life history and the origin of metamorphosis. Reviews in Fish Biology and Fisheries. 2018;28: 825–838. doi:10.1007/s11160-018-9536-z

23. Dawson HA, Quintella BR, Almeida PR, Treble AJ, Jolley JC. The ecology of larval and metamorphosing lampreys. In: Docker MF, editor. Lampreys: Biology, Conservation and Control. New York, NY: Springer; 2015. pp. 75–138.

24. Sutton TM, Bowen SH. Significance of organic detritus in the diet of larval lampreys in the Great Lakes basin. Can J Fish Aquat Sci. 1994;51: 2380–2387. doi:10.1139/f94-239

25. Sutton TM. Distribution and ecology of lampreys *Lethenteron* spp. in interior Alaskan rivers. J Fish Biol. 2016; n/a-n/a. doi:10.1111/jfb.13216

26. Evans TM, Weber AM. Multiple stable isotopes identify sediment organic matter as the primary nutritional source to American brook lamprey larvae along a stream gradient. Can J Fish Aquat Sci. 2020;77: 81–89. doi:10.1139/cjfas-2018-0270

27. Evans TM, Bellamy AR, Bauer JE. Radioisotope and stable isotope ratios ($\Delta^{14}C$, $\delta^{15}N$) suggest larval lamprey growth is dependent on both fresh and aged organic matter in streams. Ecology of Freshwater Fish. 2019;28: 365–375. doi:10.1111/eff.12459

28. Youson JH, Holmes JA, Guchardi JA, Seelye JG, Beaver RE, Gersmehl E, et al. Importance of condition factor and the influence of water temperature and photoperiod on

metamorphosis of sea lamprey *Petromyzon marinus*. Can J Fish Aquat Sci. 1993;50: 2448–2456.

29. O'Boyle R, Beamish F. Growth and intermediary metabolism of larval and metamorphosing stages of the landlocked sea lamprey, *Petromyzon marinus* L. Environ Biol Fishes. 1977;2: 103–120.

30. Manzon RG, Youson JH, Holmes JA. Lamprey Metamorphosis. 1st ed. In: Docker MF, editor. Lampreys: Biology, Conservation and Control. 1st ed. New York: Springer; 2015. pp. 139–214.

31. Lowe DR, Beamish FWH, Potter IC. Changes in the proximate body composition of the landlocked sea lamprey *Petromyzon marinus* (L.) during larval life and metamorphosis. J Fish Biol. 1973;5: 673–682. doi:10.1111/j.1095-8649.1973.tb04503.x

32. Hardisty MW. The growth of larval lampreys. J Anim Ecol. 1961;30: 357–371.

33. Hollett A. A feasibility study to determine the immediate source of carbon filtered by *Petromyzon marinus* ammocoetes from the Root River, Sault Ste. Marie through the use of stable isotopes analysis. Senior Honors Thesis, University of Waterloo. 1995. Available: http://www.glfc.org/research/reports/Hollet.PDF

34. Hayden B, Ferron M, Cunjak R, Samways K. Fruit of the forest - larval sea lamprey *Petromyzon marinus* are fuelled by allochthonous resources. Journal of Fish Biology. 2019;95: 781–792. doi:10.1111/jfb.14064

35. Folch J, Lees M, Stanley GHS. A simple method for the isolation and purification of total lipids from animal tissues. Journal of Biological Chemistry. 1957;226: 497–509.

36. Evans TM. Assessing food and nutritional resources of native and invasive lamprey larvae using natural abundance isotopes. M.S. Thesis, The Ohio State University. 2012. Available: http://etd.ohiolink.edu/view.cgi/Evans%20Thomas%20M.pdf?osu1343410278

37. Evans TM, Lampman RT. Comparison of stable isotope ratios in larval Pacific lamprey tissues and their nutritional sources when reared on a mixed diet. Aquaculture. 2019;503: 499–507. doi:10.1016/j.aquaculture.2019.01.012

38. Barceló LP, Seminoff JA, Vander Zanden HB, Jones TT, Bjorndal KA, Bolten AB, et al. Hydrogen isotope assimilation and discrimination in green turtles. Journal of Experimental Biology. 2021;224: jeb231431. doi:10.1242/jeb.231431

39. R Core Team. R: A Language and Environment for Statistical Computing. Vienna, Austria: R Foundation for Statistical Computing; 2022. Available: http://www.R-project.org/

40. Martins E, Almeida PR, Quintella BR, da Silva MG, Lança MJ. Muscle fatty acid profiles of sea lamprey (*Petromyzon marinus* L.) indicate the use of fast metabolized energy during ontogenesis. Fish Physiol Biochem. 2018 [cited 30 Oct 2018]. doi:10.1007/s10695-018-0580-3

41. Finlay JC, Power ME, Cabana G. Effects of water velocity on algal carbon isotope ratios: Implications for river food web studies. Limnol Oceanogr. 1999;44: 1198–1203. doi:10.4319/lo.1999.44.5.1198

42. Moore JW, Mallatt JM. Feeding of larval lamprey. Can J Fish Aquat Sci. 1980;37: 1658–1664. doi:10.1139/f80-213

43. Moore JW, Beamish FW. Food of larval sea lamprey (*Petromyzon marinus*) and American brook lamprey (*Lampetra lamottei*). J Fish Res Board Can. 1973;30: 7–15.

44. Boecklen WJ, Yarnes CT, Cook BA, James AC. On the use of stable isotopes in trophic ecology. Annu Rev Ecol Evol Syst. 2011;42: 411–420.

45. Yurkowski DJ, Hussey NE, Semeniuk C, Ferguson SH, Fisk AT. Effects of lipid extraction and the utility of lipid normalization models on $\delta^{13}C$ and $\delta^{15}N$ values in Arctic marine mammal tissues. Polar Biol. 2015;38: 131–143. doi:10.1007/s00300-014-1571-1

Figure 1

Click here to access/download;Figure;Fig 1.tiff

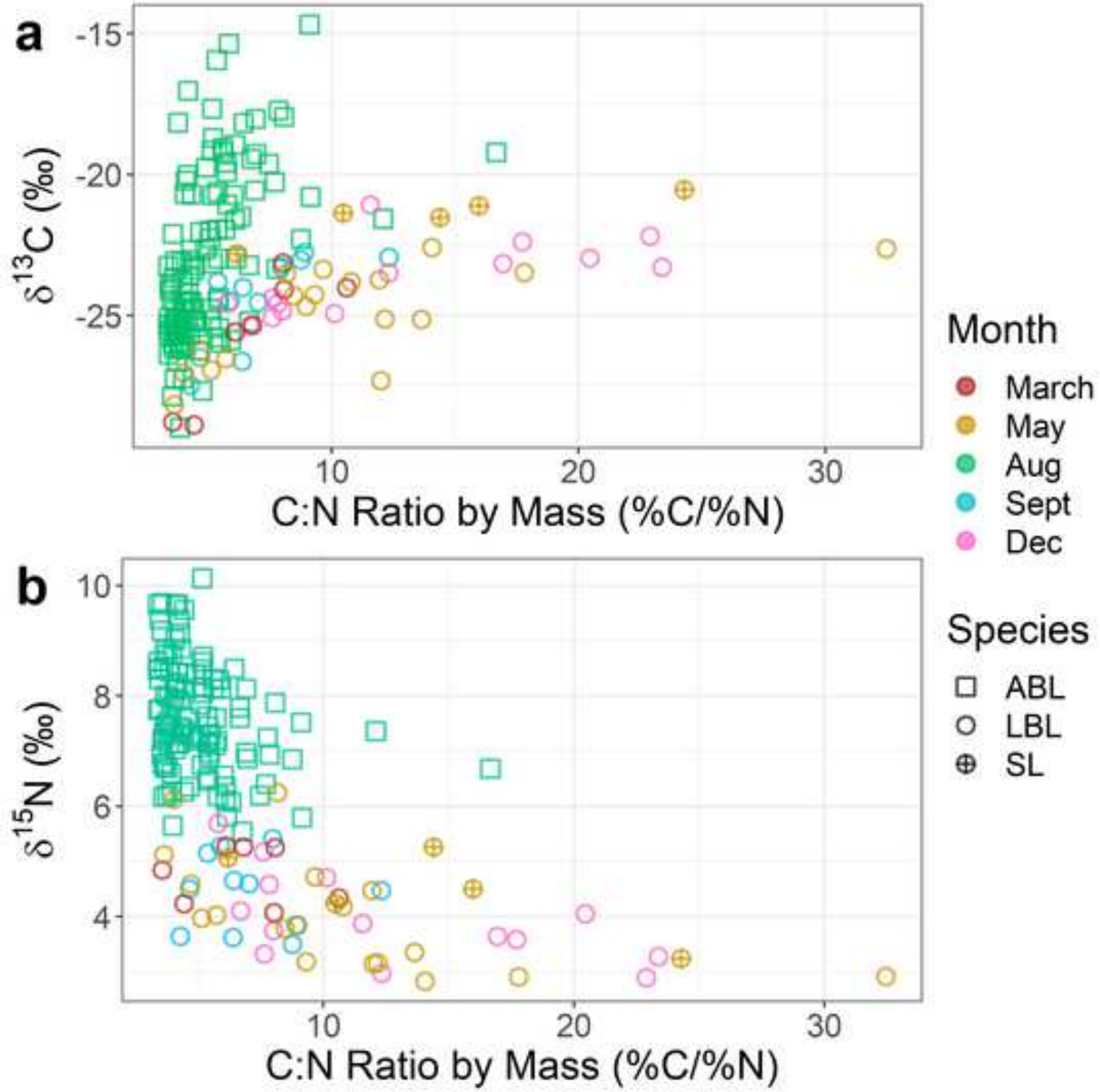

Figure 2

Click here to access/download;Figure;Fig 2.tiff

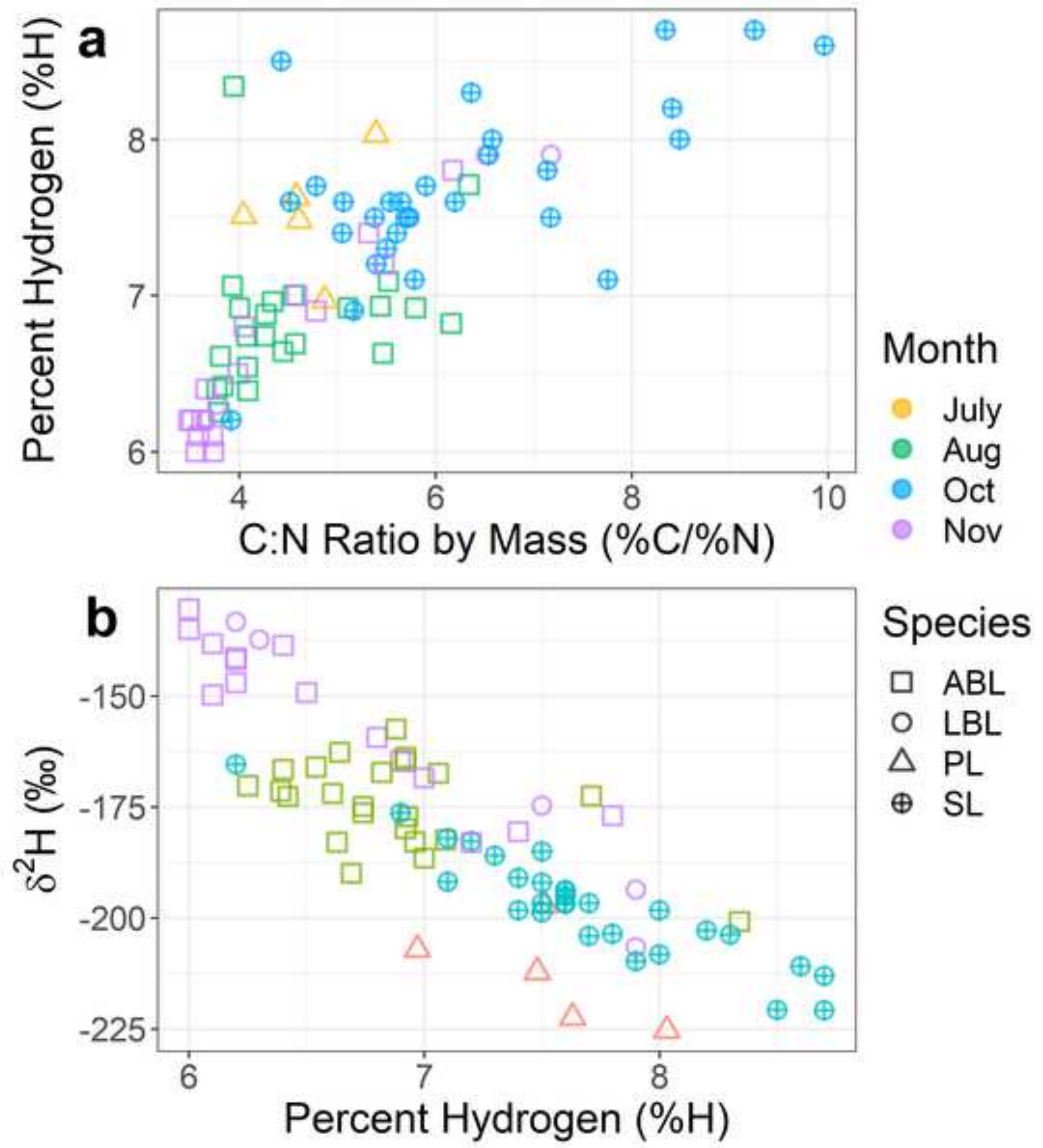

 Click here to access/download;Figure;Fig 3.tiff

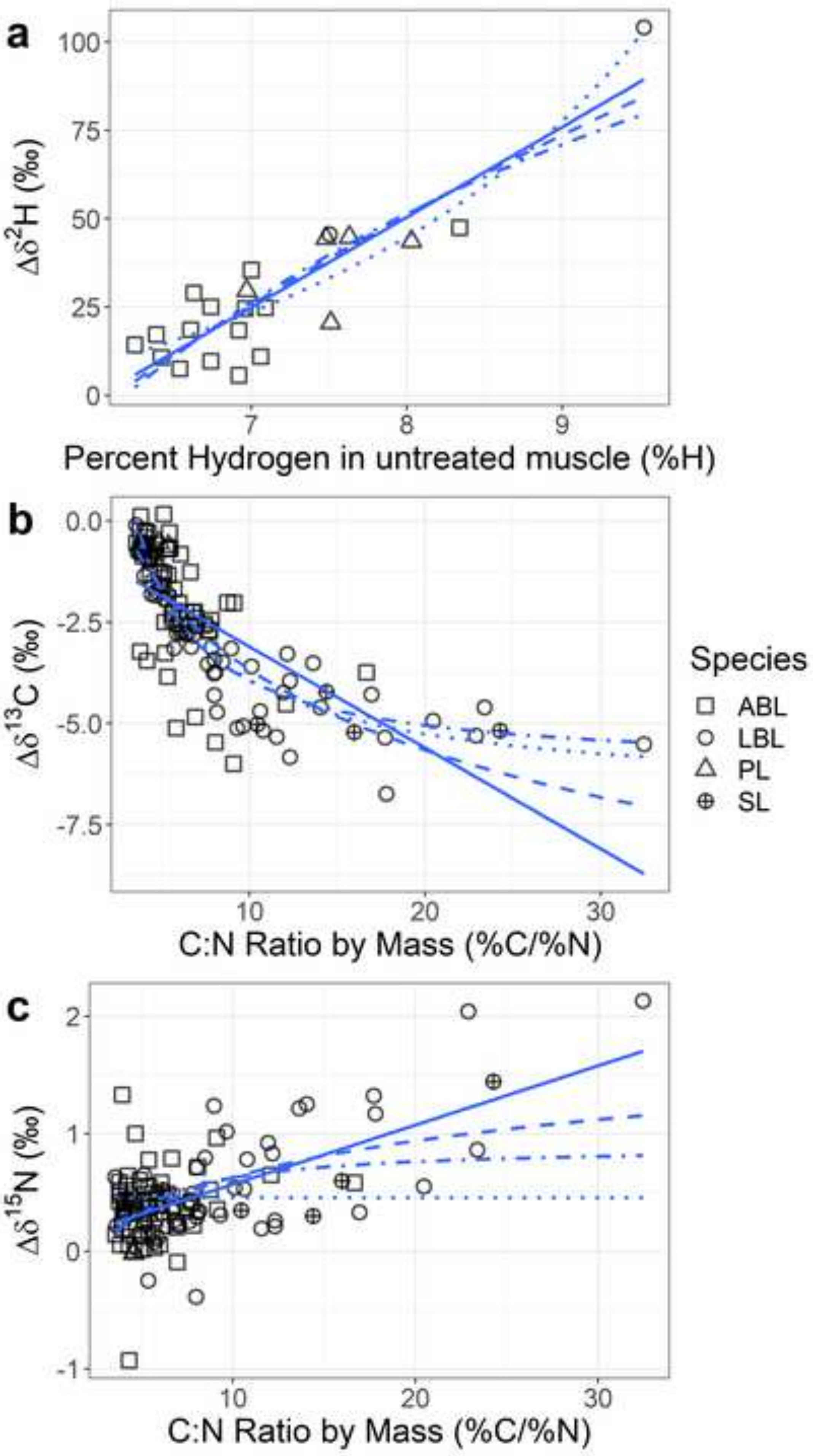

Figure 4

Click here to access/download;Figure;Fig 4.tiff

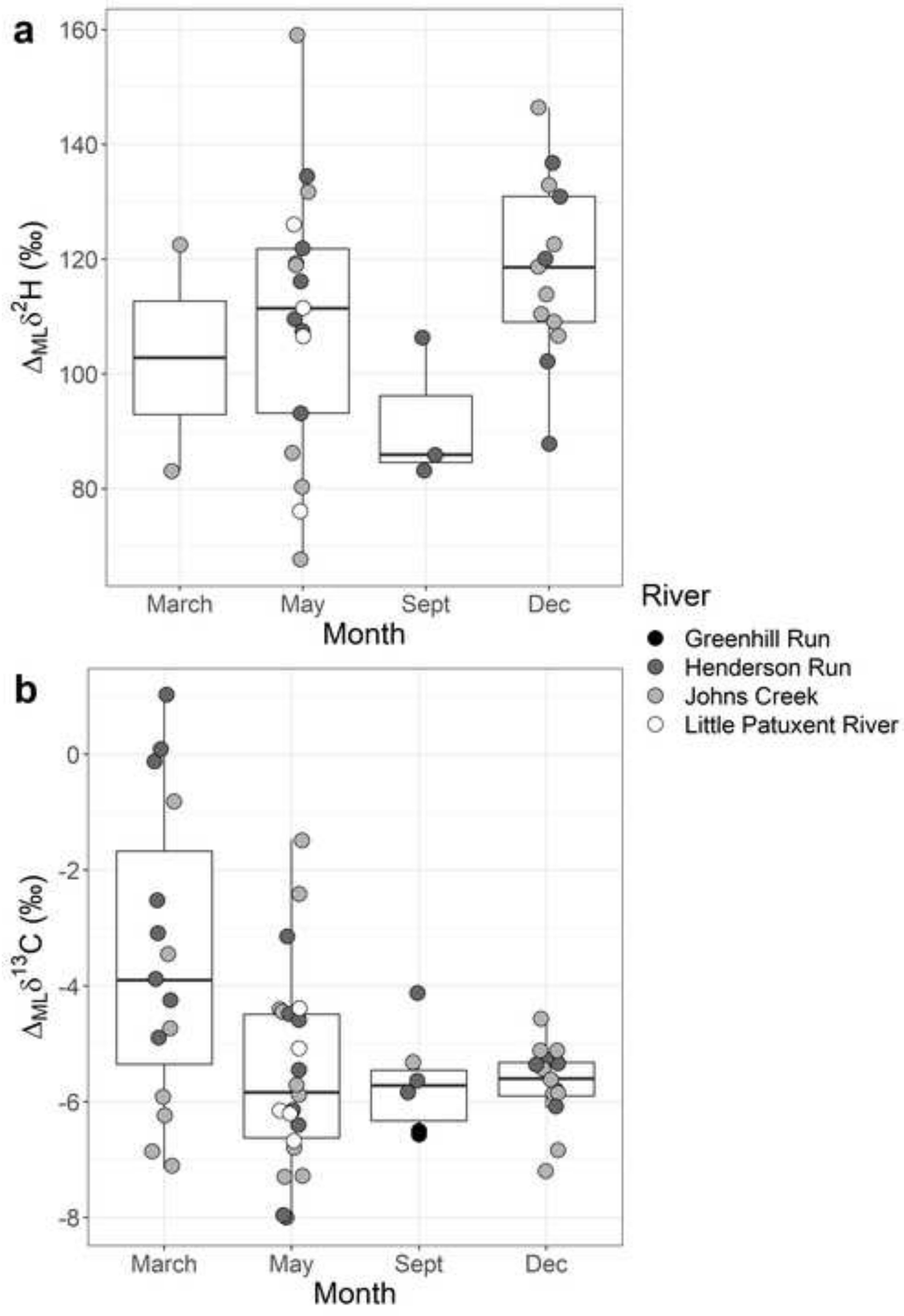

Click here to access/download
Supporting Information - Compressed/ZIP File Archive
Plos One Supl data.zip
