# Supplementary material for: Are lipids always depleted? Comparison of hydrogen, carbon, and nitrogen isotopic values in the muscle and lipid of larval lampreys
Source: PLoS One. 2024 Jan 11;19(1):e0286535. doi: 10.1371/journal.pone.0286535 (PMC10783746; doi:10.1371/journal.pone.0286535)
Supplement: Attachment — Submitted filename: Response to Reviewers.docx [file pone.0286535.s005.docx]

PONE-D-22-27112

Are lipids always light? Lipids in larval lampreys are high in 13C but low in 2H relative to muscle

PLOS ONE

Dear Dr. Evans,

Thank you for submitting your manuscript to PLOS ONE. After careful consideration, we feel that it has merit but does not fully meet PLOS ONE’s publication criteria as it currently stands. Therefore, we invite you to submit a revised version of the manuscript that addresses the points raised during the review process.

==============================

As you will see below, your manuscript has now received two reviews from experts in your field. Both the reviewers and myself agree that this manuscript has substantial value and would be of great interest to those working in stable isotope ecology. However, I also agree with both reviewers that the manuscript requires major revisions before it can be accepted for publication.

At times I found the manuscript difficult to follow. Specifically, I think the results need to be improved so that they are easier to understand and more directly address the original hypotheses. I thought the paper did a good job of setting up of the aims and goals, but that the methods and results did not clearly answer the hypotheses. For example, I think there needs to be more direct discussions in the results about the comparisons between treated and untreated samples, in particular for carbon. Those direct comparisons are not explicitly discussed in the results, despite it being stated as one of the main purposes of the paper. Given the introduction is clear about its hypotheses, addressing those hypotheses should be made equally clear in the results.

The results regarding the difference in delta13C values between untreated and extracted samples isn’t mentioned in the results text and instead the results jump straight to focusing on the best fit model. I realize the comparisons between untreated and extracted can be determined from figure 3, but because they are not clearly discussed in the text, that important and baseline result gets lost to the reader.  Perhaps a simpler figure than figure three with direct comparisons between treated and untreated samples as well as some specific commentary on the differences between each group would make things clearer.

Both reviewers have also raised valid concerns about the statistical analysis that need to be addressed or clearly rebutted. Again, some of these concerns may stem from a lack of clarity in the text.

Please read over all the suggestions for improvements with particular attention to comments on improving the overall clarity, detail, methods, and flow of the manuscript. Once again, thank you for your interesting work.

==============================

Please submit your revised manuscript by Feb 19 2023 11:59PM. If you will need more time than this to complete your revisions, please reply to this message or contact the journal office at [plosone@plos.org](mailto:plosone@plos.org). When you're ready to submit your revision, log on to <https://www.editorialmanager.com/pone/> and select the 'Submissions Needing Revision' folder to locate your manuscript file.

Please include the following items when submitting your revised manuscript:

- A rebuttal letter that responds to each point raised by the academic editor and reviewer(s). You should upload this letter as a separate file labeled 'Response to Reviewers'.
- A marked-up copy of your manuscript that highlights changes made to the original version. You should upload this as a separate file labeled 'Revised Manuscript with Track Changes'.
- An unmarked version of your revised paper without tracked changes. You should upload this as a separate file labeled 'Manuscript'.

If you would like to make changes to your financial disclosure, please include your updated statement in your cover letter. Guidelines for resubmitting your figure files are available below the reviewer comments at the end of this letter.

If applicable, we recommend that you deposit your laboratory protocols in [protocols.io](http://protocols.io) to enhance the reproducibility of your results. Protocols.io assigns your protocol its own identifier (DOI) so that it can be cited independently in the future. For instructions see: <https://journals.plos.org/plosone/s/submission-guidelines#loc-laboratory-protocols>. Additionally, PLOS ONE offers an option for publishing peer-reviewed Lab Protocol articles, which describe protocols hosted on [protocols.io](http://protocols.io). Read more information on sharing protocols at <https://plos.org/protocols?utm_medium=editorial-email&utm_source=authorletters&utm_campaign=protocols>.

We look forward to receiving your revised manuscript.

Kind regards,

Samantha E.M. Munroe
Academic Editor
PLOS ONE

Journal Requirements:

Following the need to include a marked and unmarked manuscript, we have chosen to include line numbers that match the marked document in this letter to allow reviewers to see exactly how their comments have been incorporated.

When submitting your revision, we need you to address these additional requirements.

1. Please ensure that your manuscript meets PLOS ONE's style requirements, including those for file naming. The PLOS ONE style templates can be found at <https://journals.plos.org/plosone/s/file?id=wjVg/PLOSOne_formatting_sample_main_body.pdf> and 
<https://journals.plos.org/plosone/s/file?id=ba62/PLOSOne_formatting_sample_title_authors_affiliations.pdf>

We apologize for issues around the style. We worked hard, but the links provided demonstrated that we failed with several requirements. We have carefully gone back through the document and tried to exactly match the manuscript to the examples.

2. We note that you have included the phrase “data not shown” in your manuscript. Unfortunately, this does not meet our data sharing requirements. PLOS does not permit references to inaccessible data. We require that authors provide all relevant data within the paper, Supporting Information files, or in an acceptable, public repository. Please add a citation to support this phrase or upload the data that corresponds with these findings to a stable repository (such as Figshare or Dryad) and provide and URLs, DOIs, or accession numbers that may be used to access these data. Or, if the data are not a core part of the research being presented in your study, we ask that you remove the phrase that refers to these data.

We did not originally show residual plots but have now included these as a supplementary figure and cited them in the text. We felt this was the most appropriate way to address this concern, given most readers will be unlikely to want to see these, but, as both reviewers noted, we needed to do a better job of showing model fitting/testing.

3. We note that you have stated that you will provide repository information for your data at acceptance. Should your manuscript be accepted for publication, we will hold it until you provide the relevant accession numbers or DOIs necessary to access your data. If you wish to make changes to your Data Availability statement, please describe these changes in your cover letter and we will update your Data Availability statement to reflect the information you provide.
All relevant data are now within the manuscript and its Supporting Information files.

Reviewers' comments:
Reviewer's Responses to Questions

**Comments to the Author**

1. Is the manuscript technically sound, and do the data support the conclusions?

The manuscript must describe a technically sound piece of scientific research with data that supports the conclusions. Experiments must have been conducted rigorously, with appropriate controls, replication, and sample sizes. The conclusions must be drawn appropriately based on the data presented.

Reviewer #1: Yes

Reviewer #2: No

2. Has the statistical analysis been performed appropriately and rigorously?

Reviewer #1: Yes

Reviewer #2: No

3. Have the authors made all data underlying the findings in their manuscript fully available?

The [PLOS Data policy](http://www.plosone.org/static/policies.action#sharing) requires authors to make all data underlying the findings described in their manuscript fully available without restriction, with rare exception (please refer to the Data Availability Statement in the manuscript PDF file). The data should be provided as part of the manuscript or its supporting information, or deposited to a public repository. For example, in addition to summary statistics, the data points behind means, medians and variance measures should be available. If there are restrictions on publicly sharing data—e.g. participant privacy or use of data from a third party—those must be specified.

Reviewer #1: Yes

Reviewer #2: Yes

4. Is the manuscript presented in an intelligible fashion and written in standard English?

PLOS ONE does not copyedit accepted manuscripts, so the language in submitted articles must be clear, correct, and unambiguous. Any typographical or grammatical errors should be corrected at revision, so please note any specific errors here.

Reviewer #1: Yes

Reviewer #2: Yes

5. Review Comments to the Author

Please use the space provided to explain your answers to the questions above. You may also include additional comments for the author, including concerns about dual publication, research ethics, or publication ethics. (Please upload your review as an attachment if it exceeds 20,000 characters)

Reviewer #1: I have had the opportunity to review the manuscript titled “Are lipids always light? Lipids in larval lampreys are high in 13C but low in 2H relative to muscle.” Overall, I found this to be an important study that is generally clear and well-written. The authors found that 13C in lampreys becomes depleted with lipid extraction instead of enriched, opposite the general pattern. This has important implications for ecological inferences with stable isotopes, as the authors point out it makes them appear more like predators instead of detritivores. As such, important caveats in isotope ecology such as these are important to address. I think the authors do a good job of highlighting the importance of this and in setting up and carrying out their study.

We thank the reviewer for their time and effort, their work has greatly improved the manuscript.

I do have several issues that should be addressed before moving forward with the manuscript. Namely, other causal sources for the pattern the authors found should be included, such as difference in the effects of lipids and lipid extraction among trophic levels and potential effects of growth patterns throughout the season on isotopic discrimination. In addition to these perspectives on their results, the authors should include more information of the errors of their predictive models.

The reviewer has made many excellent suggestions which we have worked to incorporate, and this has greatly improved the manuscript. We now more fully explore models fits and incorporated papers which they cited. We are thankful for their patience and interest in seeing the paper improved.

Title
Should the end of the title be “but low in 2H in muscle?” The authors used muscle as their main tissue, so it isn’t in relation to muscle, it is in muscle.

L4-6: The title has been reworked following both reviewers’ comments.

**Abstract**
Line 17: Change to ‘Isotope ratios…’
L30: Changed to mirror the preceding section dealing with carbon.

Introduction
Line 41: I wouldn’t call this manipulation internal sources because it is really discrimination of isotope values during incorporation.

L60-61: Changed ‘manipulation internal sources’ to ‘isotope discrimination during the generation of new biomolecules’ following the reviewer’s comments.

Line 48: Nitrogen isotopes can be affected by lipid extraction in some tissues, which is why lipid extraction can be expensive because samples potentially need to be run twice, once lipid extracted for carbon and then another non-lipid extracted for nitrogen.

L74-78: We have now added wording highlighting these points in the paragraph emphasizing the issues with nitrogen measurements when accounting for lipids.

Line 57: comma before ‘and.’

This section has been substantially rewritten.

Line 69: Over what sort of time period? The entire larval stage?
L99: Changed to clarify this refers to the entire larval period.

Line 72: Do the authors mean at the end of their larval stage or do they undergo a metamorphosis at the end of each summer that they are larvae. Please clarify.

L102-103: We have clarified metamorphosis occurs at the termination of the larval period.

**Methods**
Line 109: Suggest moving “with a high-powered setting” so it is after “immobilized” and before “when they were observed.” Delete “was activated.”
L145-146: Changed following the reviewer’s recommendation.

Line 204-207 and Table 1: Most lipid correction studies use standard measures of error between predicted and observed values and include mean squared error, mean absolute error, and proportion predicted values with < 0.5 per mil difference from observed values. Also, provide delta AIC and AIC weights. These are standard for these types of analyses and should be used here as a measure of error and to compare the models.

L371-377: These components have now been added to the manuscript and into Table 1. We have added a ‘model efficiency’ estimate, not using a set 0.5‰ value, but relying on two times machine standards. We felt this provided a fairer comparison given the variability in machine measures of each isotope ratio. Our approach to selecting models has also been updated in the results, though we continue to present all models to allow readers the opportunity to interpret these results as well.

Table 1 and associated results. Use other forms of error MSE, etc… This should help in selecting for the correct model. Also deltaic should also be presented since that is what matters most.
L352-403: Following the reviewer’s comment we have added ΔAIC, MSE, and MAE to Table 1 to help readers compare models. We have also used these to show how we selected the top models in the results.

Line 263: Awkward phrasing, maybe “fitted values closer to a CN of 3.5 had less variation than values further from 3.5.” Was there some pattern to the residuals that might be better fit by adding a variance structure as a random effect? See Zuur et al. 2009 Chp 4. Mixed effects models and extensions in ecology with R.

We considered mixed effects models, and explored using site as a random effect, but these models did not improve fits. The inclusion of MSE and the plots of the residuals will hopefully provide evidence that the models we consider include members that fit the data well.

**Discussion**
Consider trophic level in lipid effects. Other studies have found that lower trophic level species have less discrimination of carbon-13 during lipid synthesis, but I’m not aware of the effects for detritivores. See:

Cloyed, CS, DaCosta, KP, Hodanbosi, MR, Carmichael RH (2020). The effects of lipid extraction on carbon-13 and nitrogen-15 values and use of lipid-correction models across tissues, taxa, and trophic groups. Methods in Ecology and Evolution 11, 751-762.

L609-615: We have included this paper when we discuss glycogen in larval lampreys to further stress the importance of body stores and the crude nature of C:N values. We agree with the reviewer that glycogen is likely to play an important role in the isotope values we observed.

Also, consider how growth may affect the different patterns among months? Are there different growth patterns throughout the year, especially since the authors mention they are very slow growing. Oftentimes, more growth results in less discrimination. One potential explanation is that during growth, more available material is needed, and organisms are more likely to have less discrimination.
L582-607: We have added considerably to an older paragraph and an entirely new paragraph to the discussion to deal with these points (i.e., seasonality, growth rate, discrimination variation) and increased the number of sources discussing this.

Line 299: You mean low metabolic rates?

L594-596: We have deleted this sentence

Lines 317: The Cloyed et al. 2020 above is a recent meta-analysis that also found lipid-extraction can affect nitrogen values. Can’t remember what their results were exactly but would be worth including here.

L582-607: We have included this paper during this section, and stressed that prior work has often focused on fishes, but the importance of diet should also be considered.

Line 344: Other work has also found similar patterns.
Patterson, HK, Carmichael, R.H. (2016). The effect of lipid extraction on carbon and nitrogen stable isotope ratios in oyster tissues: implications for glycogen-rich species. Rapid Communications in Mass Spectrometry 30, 2594-2600

We agree with the reviewer that this work demonstrates that C:N values in an organism are a bulk measure and not necessarily reflective of lipids alone. The authors do a good job in this paper demonstrating these differences are driven by stored glycogen in tissues. However, when they measured tissue with high lipid content, as predicted, δ^13^C rose because the lipids were presumably depleted. We are not aware of a paper which has measured lipids directly and found that they are the driving component of rising δ^13^C values.

L582-594: Larval lamprey glycogen content has been studied before (O’Boyle and Beamish 1977), and these authors found that it changes seasonally. This was not included in our discussion, and should have been because of the importance of stressing that C:N is a bulk measure and does not necessarily reflect lipids. We have included Patterson and Carmichael now and discussed the possibility of glycogen’s impact on our stable isotope values.

O'Boyle, R. N., & Beamish, F. W. H. (1977). Growth and intermediary metabolism of larval and metamorphosing stages of the landlocked sea lamprey, Petromyzon marinus L. *Environmental Biology of Fishes*, *2*(2), 103-120.

**Reviewer #2:**

Dear Authors, thanks for the opportunity to review this manuscript that explored the effect of lipids on isotopic signatures of carbon, nitrogen, and hydrogen in larvae lampreys, and the relationship with lipid proxies (%H and CN ratio). I am convinced that your manuscript deserves publication, but based on my experience and review; it may require some major revisions because this study has a number of flaws (kindly refer to the comments below and the comments in the manuscript pdf for more details), which makes it challenging to accept it for publication at this stage.

We thank the reviewer for their kind words, and their help in improving the manuscript.

The reviewer made numerous small edits to improve the grammar and readability of the manuscript. To keep this response concise, we have not noted when each has been accepted, but we have gone through and considered each one. However, whenever a comment was left, we have transcribed it here and responded to it. We thank the reviewer for the time they dedicated to this, we appreciate how much effort and care they took in reading the document.

My major comment is you need to clarify the objectives of the study, the statistical tests that you used, and the results. My concern is that the statistical analyses and results presented in the manuscript seem disconnected from the objective and hypotheses. The interpretation of some results observed in the results section is unclear in the discussion section and did not match with the hypotheses. Some results were presented in the result section but data were not presented in the manuscript neither in a table or a figure. The flow of arguments is not always easy to understand.

We have worked throughout the document to clarify why we approached the data in the way we did (including considerably rewriting the end of the introduction, L118-129). We have also added more explorations with linear models and hope that the review feels that it is easier to understand.

I agree the importance of identifying the effect of lipids on carbon, hydrogen, and nitrogen isotopic signatures in larvae lampreys because such information will contribute to better interpreting the hydrogen isotopic values in lampreys and to better understanding the lipid effect on stable isotope signatures of carbon, nitrogen, and hydrogen. I’ve listed below some points that can be considered to continue improving the manuscript before publication:
The reviewer worked very hard to provide a rich review. Their work has helped us as we improved the manuscript. We thank them for this effort and time commitment.

**Major comments**
1- I would change the title because “light” is confusing. Did you mean depleted? Also, you measured nitrogen and it doesn’t appear in the title. An idea: “Are lipids always isotopically depleted? Comparison of carbon, nitrogen, and hydrogen isotopic values of lipids in larval lampreys relative to muscle”

L4-6: Based on this comment and the first reviewer we have changed the title to better match the content of the paper and purpose of the work. We appreciate the reviewer providing a descriptive and concise title for consideration.

**Abstract**

L18-21: I would suggest to modify this sentence to clarify. Something like this: "... in Maryland. The isotopic ratios were measured in both bulk and lipid-free muscle samples, but also in extracted lipid samples."

L35-37: We have rewritten this sentence following the reviewer’s comment.

L27-28: I'm not sure that I would finish my abstract with this sentence. Why don't you change for the implication of your study?

L44-46: We have deleted the sentence and agree with the reviewer it was not appropriate. We feel the preceding sentence is a better summary of our findings and points to the wider impacts of our findings.

**Introduction**

L35: Please clarify. Enzymatic reaction? Metabolic reaction? Photosynthetic reaction?

L54: Clarified to enzymatic reaction in the text now following the reviewer’s comment.

L37: What do you mean by starting materials? food sources? Diet?

L56: We have clarified that this paper is dealing with dietary sources.

L38: What do you mean by predictable? Not sure that it is predictable. It is assumed 1‰ for carbon and 3‰ for nitrogen but it is highly variable among organisms, tissues, body condition, etc.

L57: We have deleted ‘predictable.’ Originally our intent was to imply that there are expectations based on enzymatic kinetics, but we agree it implied a discrete exact quantity, which is not true; the actual difference is quite variable.

L47: I would change the reference here. Because Logan et al. 2008 did not study that and its sentence said that most lipid classes contain no nitrogen had 3 references: Schmidt et al. 2003, Bodin et al. 2007, Post et al. 2007.

L66-68: We have removed this sentence.

L47-48: Have you a reference to support that affirmation?

L67-68: We have deleted this sentence.

1- I found the paragraph at lines 49-61 confusing. It seems to have two ideas: one about the need to correct for the lipid effect for the carbon and hydrogen isotopes and not for nitrogen isotope, and the second one about the estimation of lipid content and the proxy associated (%H, CN ratio). I do not understand the idea of this paragraph. Did you talk about the extraction of lipids in sample that increase the cost of a study to measure stable isotope ratios? If yes, I would change the beginning of the sentence for something like this: “However, extraction of lipids in sample can increase the cost of the study and the number of samples necessary because carbon is analyzed on a lipid-extracted subsample while nitrogen is analyzed on a bulk subsample”. If you talk about the necessity to measure the lipid content, I think you must clarify this entire paragraph.

L69-80: Reviewer 1 also recommended changes to this paragraph, and we have substantially altered it. This paragraph now deals with the issue of lipid extraction and mathematical corrections as preferred. The following paragraph then deals with C:N ratios and the use of this to mathematically predict the lipid-free value.

L50: I would add a sentence about the nitrogen here such as "In contrast, nitrogen isotopes are not affected by the presence of lipids..."

L75-76: Here we point to the mechanism that could alter N isotope ratios.

L77-78: High means enriched? And the diet of larvae lamprey is also highly variable and include detritus, terrestrial and aquatic sources? Is it a possible explanation? Why carbon ratios of lamprey larvae are unexplainably high? Do you have some possible explanations?

L107-110: We did not want to spend long periods of time covering the prior papers, so we kept the language here brief.

Prior to this work there was no good explanation for the enriched ^13^C values unless lipids drive them. The prior studies have considered the solutions the reviewer puts forward, and none have been satisfactory in fully explaining the behavior.

2- At lines 81-86, I think that the good approach is to account for the lipid effect either by extracting lipids or by correcting mathematically (if mathematical models were available and validated for lamprey) for carbon and hydrogen isotopes prior to trophic and foraging interpretation. It is well known in the literature that lipids may biases interpretation of food web structure and diet estimates. Indeed, it is necessary to correct for the presence of lipids where body condition vary substantially due to lipid accumulation or loss, especially in your case for larvae of lampreys.

This and the next comment are closely related We agree this is the best approach, but this has not been done for lamprey.

3- I found the objectives of the study very confusing at lines 87-98 and also the statistical analyses used. If I understand well, you had 2 objectives: 1) to evaluate C, N, H isotopic signatures of lipids and compare to isotopic signatures of larvae lamprey muscles lipid-free and bulk, and to see if there is difference between lipids and muscle in order to confirm or not the expectation that the δ2H will be enriched in muscle after lipid-extraction, the δ13C will be depleted, and the δ15N will be unchanged, and 2) to evaluate the best relationship (linear or not) between the difference ΔδxX in lamprey muscle vs. the lipid proxy (%H, CN)? I may be confused but you need to clarify the objectives of the study to avoid misleading.
L124-129: We have worked to rewrite the last paragraph of the discussion and clearly introduce the objectives of this study, following the pattern laid out by the reviewer.

4- Also, in an another manuscript, you will be able to establish a mathematical equation to correct for the lipid effect if samples were analyses with lipids (see Post et al. 2007, Logan et al. 2008, Lesage et al. 2010, GroB et al. 2021 and more other). It will be a great article.

We appreciate the encouragement. We would like to do such work with both wild populations and rearing experiments.

5- You first hypothesis was that larvae lamprey lipids would be enriched in 13C relative to muscle, and therefore the δ^13^C of muscle would decline after lipid extraction. You had no data and results presented in our article about this hypothesis. You may present a graph δ^13^Cbulk vs. δ^13^Clipid-free and test the relationship. The second hypothesis was that larvae lamprey lipids would be depleted in ^2^H. Same here, no data was presented in the article. Also, the larvae lamprey lipids would be depleted in ^2^H relative to muscle lipid-free? The third hypothesis was that the δ^15^N would be unchanged by lipid extraction. No data presented again. You may test it with a paired t-test and presents a graph δ^15^Nbulk vs. δ^15^Nlipid-free.

L214-334: The results of these types of analyses are shown in Fig 4 because it is the Δδ^x^Y (the difference between bulk and lipid-extracted samples). However, it is clear we did a poor job of stepping through this analysis and demonstrating the need for this figure. Therefore, we have added the recommendation to include three t-tests, examining if lipid extraction had any effect of samples a priori, and then exploring how a proxy for lipids might better predict this difference.

**Methods**
6- You used the passive form and the active form in this section. You may choose one or the other.

We have tried to correct these now.

L108-110: “…when they were observed outside the sediment, larvae were immobilized with a high-power setting.”

L145-146: This sentence was also highlighted by the first reviewer and has been rewritten.

7- Lines 114-115, please add more information about the dissection. Which part did you dissect? Muscle? Location of the muscle sample? Dorsal? Lateral?

L153-157: We have added detail of how muscle was dissected.

L116: I would put the permit number here in [ ]

L159: We have added the permit number in the manuscript now.

8- Lines 117-124, please add more information about the protocol you used. Did you dry using a freeze-drier? Did you freeze sample at -80 before? After drying, did you powdered your muscle sample with a mortar and pestle? Cryomill? How long did you wash your sample in the chloroform-methanol solution? In a ultrasonic bath? At 4°C? Ambient temperature?

L161-162, 169: We have added these details to the methods now.

L143-144: Add references here

L190: We have now cited Post et al 2007 here, as they use this language.

9- Lines 144-145, you mentioned that you had the larvae lipid samples from Maryland only. What did you measure? Lipid content? Stable isotope ratios?
L190-191: This has been clarified, it was the stable isotope ratios.

L152-153: Were the samples powdered? Give more details about the weight in brackets (e.g. 0.350-0.500 mg).

The muscle samples were powder, but lipids were an oily fluid. We mention the appearance of lipids and muscles in the preparation section (L167-170). We have now added the amount of material packed in the description (L198-199).

L157: Which standards? I think that it is very important for H because of the air moisture (Vander Zander et al. 2016)

L204-205: We have added the H internal standards because these include ones available for purchase. The standards for ^13^C/^15^N were things such as powder deer hoof are not available outside of this lab and would not provide much help for comparison between labs.

10- Lines 170-183, if you have not created these equations, you should put the references associated with equations 1 to 4 (e.g. Logan et al. 2008 for eqn 2, McConnaughey et McRoy for eqn 3…).

Both equations 2 and 3 are cited as being drawn from Logan already. McConnaughey and McRoy did not suggest any of the equations we relied upon. We did not cite the linear model (because of its simplicity) or eqn 4 because it is a simplification of the Logan et al. model. We included the function in R for completeness that was used as well. For instance:

“We also fit the data with a non-linear model [11] (fit by the nls function in R)”

[11] Is the reference to Logan. We want to make it clear that we are relying on Logan’s excellent summary and not proposing a new approach. If the reviewer still feels we failed to attribute these we are open to specific language to alter the sentence.

11- Lines 170-183, you need to clarify the utility of the four models used. Is it to establish the best relationship (linear or not) between the difference ΔδxY in lamprey muscle vs. the lipid proxy (%H, CN)? These models did not answer to your hypotheses previously mentioned.

L214-225, 264-269: We have worked to clarify the introduction following the reviewer’s suggestions, added considerable explanation to this in the methods, and now feel this should more naturally explain why we used models at this point.

12- You selected the best supported model with the AIC criteria but did you evaluate the models? Did you compare the predicted and observed ΔδxY values? You will obtain R2, MAE, and you will see if the slope is close to 1 (no difference between the predicted vs. the observed values)? I think it is what you did at lines 237-241 with the t-test but it was not clear enough.

L246-248, 264-269: Based on this comment and reviewer 1’s comments, we have greatly increased the materials presented around the models and modified this section in the manuscript.

13- Lines 199-201, I have not read in details about that, but if the larvae accumulate a lot of lipids in their bodies during spring and their conditions declined throughout the summer to undergoes a metamorphosis at the end of the summer which they do not feed and rely on accumulated lipid stores (lines 69-74), do you think that it could impact the lipid synthesis and metabolism? Do the lipid accumulation vs. the conversion of lipid for metabolism use the same procedure for synthesis? Also, at lines 207-209 and figure 4b, there is a difference among month. I think that we must account for the month effect in the analysis.

L276-280: We assumed enzymatic processing during lipid synthesis would be the same year-round. Our testing around seasonality emerged after we began to explore the data. Initially we did not expect it to matter, but were surprised when the data began to suggest seasonality maybe important. Based on this and the first reviewer, we have added materials in the discussion related to glycogen production (L447-459), which does help explain seasonal differences.

L203-204: I don't understand this test. You compare the isotopic signature of lipid-free of muscle vs. lipid sample? Reformulate.

L204-207: I don't understand this analysis. You want to compare the predicted difference vs. the observed difference? The results must be presented in the article. A table with all data?

L264-269: We have modified this section to more clearly lay out how we are checking the model efficiency.

14- Δ_ML_δ2H and Δ_ML_δ13C values are only presented in figure 4 by months. I think you must present these data in the article and in a table.
Each point is individually plotted on these graphs (Fig 5), the data are also available as supplementary files (S2 Fig), but we feel that the figure provides a concise way to visualize these.

15- At lines 204-207, you evaluated the reliability of the top ΔxY model, I do not understand this analysis. Did you compare the isotopic signature of lipid-free of muscle vs. lipid sample? Please clarify.
L264-280: Both reviewers were uncertain about how we examined models. We modified the end of the methods to clarify.

16- At lines, 205-207, I also don't understand this analysis. Do you want to compare the predicted difference vs. the observed difference? The results must be presented in the manuscript. A table with all data?

We used the model to estimate a value that we also measured so we felt it appropriate to see how close the model was to the measured value. We have added another figure showing the measured lipid and the lipid extracted muscle (S2 Fig).

**Results**

L214-217: Did you test it? Not really scientific to mention "was not universal"? What is the meaning of universal?

L219-222: It is mentioned regressed against, but you have not the relationship on these figures and the equation of the linear regression? non-linear? Which test did you use?

L224-226: Which test did you use to affirm that? Give the name of the test and the equation.

18- In order to affirm that the relation is positive, negative, or “appear linear”, you must use statistical tests to support these affirmations (e.g. lines 215, 216-217, 224-226).

20- I think you need to present the relationship and equations (linear or not) on figures 1 and 2, and the significance of the relationship. If linear, you need to have a R2.

L298-334: We felt that our approach here relied too much on visual inspection of figures. Both reviewers clearly felt that our approach to interrogating our data was insufficient. As a result, we have now added simple linear models to the δ^2^H vs C:N, %H vs C:N, δ^2^H vs %H because these were very linear in appearance. For the δ^13^C/δ^15^N, we have modified the language here to make it clear this is a visual exploration, not a mathematical quantification, because the relationship looked more complex.

L228: LE or not LE?

We have introduced the work ‘bulk’ to refer to dried muscle samples before lipid extraction. We added this as a subscript to the isotope ratio notation throughout the document.

L237-241: Where the data are presented?

Our statement refers to the linear model’s lack of asymptote. This was more a statement of type of model, and not a reference to the data directly. We have added information clarifying that these models can be found in Table 1 and located in Figure 4.

L243: I would put different color of each model to facilitate the visualization.

We currently use a different line type. We considered the inclusion of color, but found it made it harder to distinguish where models overlap because the layering would tend to imply the last drawn model was the top model.

L254: Which one? Try to be clearer. eqn 1 X for example

L382: Added a refence to the two non-linear equations that seemed to fit the data well.

L257-261: I did not see these data in the article.

L388-392: For this analysis we are using the model parameters (presented in table 1) to make estimates, which we present in the text. Therefore, all data are available to the reader.

17- It is not usual to have the legend of figures in the main text (see at lines 219-226, 228-232, 243-248, 285-287).

The PLOS style requires legends in text immediately after the paragraph the figures are presented in.

19- At lines 237-238, where the data is presented in the manuscript? Also, at lines 238-241, 257-261, and 265-266.

We are unsure what data are not presented in the manuscript. It appears the reviewer is concerned that by showing a difference between measured samples we are not presenting our lipid data (all other data are now presented directly in the text). We have added this as supplementary boxplot (S2 Fig) to ensure we have all materials in the text.

L274: You must test it with statistical test. Visually is not a good method.

We use the visual method first and then test this statement with an ANOVA.

21- Line 275, you have only 2 samples for March and only 3 for September. I'm not sure that you can test it with an ANOVA with this small sample size for these 2 months.
L508: We bring up this limitation now in the discussion. We agree that the sample size is very small for some months, and therefore we anticipate the power of the test to be lower. \

L287: Figure was in black and white.

We apologize if the reviewer’s image was black and white. The uploaded figure should be in greyscale, and its shades of grey identify different rivers.

**Discussion**
22- I did not review in details this section because we need to clarify the introduction, objectives, methods, and results before.

We appreciate that the reviewer may have more comments after seeing the revised manuscript.

L290: Not sure I understand why you are talking about backbone. You analysis only muscle and lipid right?

L423: Here backbone refers to the carbon scaffolding. We have modified the language to try to highlight this and avoid confusion with the term as a definition for the bones in the back of vertebrates.

L299-307: This part of the discussion help to understand which results?

L432-433: This paragraph is still discussing the unusual accumulation of ^13^C in larval lipids. We have tried to clarify this in the topic sentence.

L326: You have only a proxy of the lipids and not the lipid content. I would change the formulation.

L442-459: We have modified the text to emphasize that we support lipid as important, but we are relying on a proxy not a direct measure.

L333-336: Did you powder your samples to insure homogenization?

We tried and have elaborated in the text, but as we note, the protein component does not mix with the lipid content and tend to separate. Since the sample sizes are so small, it is nearly impossible to ensure true homogenization for δ^2^H and to a lesser degree δ^13^C/δ^15^N samples.

23- There are limitations in all studies, but none are mentioned in your study. I would have appreciated to have a section covering the potential limits or your study.
L506-517: Following the reviewer’s comments, we have added a whole paragraph dealing with this topic now.

So thanks for this interesting manuscript that explored the lipid effect on stable isotope signatures in larvae lampreys. I think that your manuscript deserves publication following major revisions.

We thank the reviewer for all their work.

6. PLOS authors have the option to publish the peer review history of their article ([what does this mean?](https://journals.plos.org/plosone/s/editorial-and-peer-review-process#loc-peer-review-history)). If published, this will include your full peer review and any attached files.


If you choose “no”, your identity will remain anonymous but your review may still be made public.


**Do you want your identity to be public for this peer review?** For information about this choice, including consent withdrawal, please see our [Privacy Policy](https://www.plos.org/privacy-policy).

Reviewer #1: No

Reviewer #2: No

[NOTE: If reviewer comments were submitted as an attachment file, they will be attached to this email and accessible via the submission site. Please log into your account, locate the manuscript record, and check for the action link "View Attachments". If this link does not appear, there are no attachment files.]

While revising your submission, please upload your figure files to the Preflight Analysis and Conversion Engine (PACE) digital diagnostic tool, <https://pacev2.apexcovantage.com/>. PACE helps ensure that figures meet PLOS requirements. To use PACE, you must first register as a user. Registration is free. Then, login and navigate to the UPLOAD tab, where you will find detailed instructions on how to use the tool. If you encounter any issues or have any questions when using PACE, please email PLOS at [figures@plos.org](mailto:figures@plos.org). Please note that Supporting Information files do not need this step.

*In compliance with data protection regulations, you may request that we remove your personal registration details at any time.* [*(Remove my information/details)*](https://www.editorialmanager.com/pone/login.asp?a=r)*. Please contact the publication office if you have any questions.*
