# Supplementary material for: Are lipids always depleted? Comparison of hydrogen, carbon, and nitrogen isotopic values in the muscle and lipid of larval lampreys
Source: PLoS One. 2024 Jan 11;19(1):e0286535. doi: 10.1371/journal.pone.0286535 (PMC10783746; doi:10.1371/journal.pone.0286535)
Supplement: Attachment — Submitted filename: Response to Reviewers.docx [file pone.0286535.s006.docx]

The article in its present form cannot be accepted for publication and authors are advised to revise the article as major revision. The article particularly has flaws presentation in terms of coherence in result and discussion part. In introduction latest refences to be used at least 80 % beyond 2017 onwards.
Thank you for allowing us to revise the manuscript. We have worked to address the comments throughout the document following the reviewer’s comments. We are not able to change the references in the introduction to meet the 80% requirement for 2017 and beyond. We selected relevant recent literature, and our sources are composed of primarily studies from the last 20 years (~70%), but the field of study is not so active as to have sufficient studies to cite since 2017.


Reviewers' comments:
Reviewer's Responses to Questions

**Comments to the Author**

1. If the authors have adequately addressed your comments raised in a previous round of review and you feel that this manuscript is now acceptable for publication, you may indicate that here to bypass the “Comments to the Author” section, enter your conflict of interest statement in the “Confidential to Editor” section, and submit your "Accept" recommendation.

Reviewer #2: All comments have been addressed

Reviewer #3: All comments have been addressed

2. Is the manuscript technically sound, and do the data support the conclusions?

The manuscript must describe a technically sound piece of scientific research with data that supports the conclusions. Experiments must have been conducted rigorously, with appropriate controls, replication, and sample sizes. The conclusions must be drawn appropriately based on the data presented.

Reviewer #2: Yes

Reviewer #3: Yes

3. Has the statistical analysis been performed appropriately and rigorously?

Reviewer #2: Yes

Reviewer #3: Yes

4. Have the authors made all data underlying the findings in their manuscript fully available?

The [PLOS Data policy](http://www.plosone.org/static/policies.action#sharing) requires authors to make all data underlying the findings described in their manuscript fully available without restriction, with rare exception (please refer to the Data Availability Statement in the manuscript PDF file). The data should be provided as part of the manuscript or its supporting information, or deposited to a public repository. For example, in addition to summary statistics, the data points behind means, medians and variance measures should be available. If there are restrictions on publicly sharing data—e.g. participant privacy or use of data from a third party—those must be specified.

Reviewer #2: Yes

Reviewer #3: Yes

5. Is the manuscript presented in an intelligible fashion and written in standard English?

PLOS ONE does not copyedit accepted manuscripts, so the language in submitted articles must be clear, correct, and unambiguous. Any typographical or grammatical errors should be corrected at revision, so please note any specific errors here.

Reviewer #2: Yes

Reviewer #3: Yes

6. Review Comments to the Author

Please use the space provided to explain your answers to the questions above. You may also include additional comments for the author, including concerns about dual publication, research ethics, or publication ethics. (Please upload your review as an attachment if it exceeds 20,000 characters)

Reviewer #2: Dear Editor and Authors, thanks for the opportunity to review this updated manuscript “Are lipids always depleted? Comparison of hydrogen, carbon, and nitrogen isotopic values of lipids in larval lampreys” that explored the effect of lipids on isotopic signatures of carbon, nitrogen, and hydrogen in larvae lampreys, the relationship of the difference between muscle samples before and after lipid extraction, and lipid proxies (%H and CN ratio), and the difference of isotopic signatures between muscle and lipid samples. I can testify that the authors have greatly improved the manuscript based on my previous comments and those made by the other reviewers. I’ve listed below some minor points that can be considered to continue improving the manuscript before publication.

General comments
1- There are few typos’ errors throughout the manuscript, I suggest that the authors reread the manuscript and pay attention to it. For example, here are some of them:
a. Line 27, a comma is missing between nitrogen, and hydrogen
b. Line 41, a comma is missing after natural systems
c. Line 54, a comma is missing after hydrogen (2H)
d. Line 201, a space is missing between (1) and where
e. Line 204, a space is missing between (2) and and
f. Line 293, a space is missing before while
g. Line 384, a space is missing between δ13C and between
h. Line 250, paragraph title is not written with the same writing style
i. etc…
We have gone through the document and made these corrections. We have also gone through the document again to correct some instances where spaces and minor grammatical issues were missed in the last revision.

2- I find that the context for the first hypothesis about the δ13C depletion after lipid extraction at line 106 is confusing. We did not understand why it could have a δ13C depletion after lipid extraction. According to the literature, if you removed depleted lipids in organism (or mathematically correct for them), the organism will become enriched in δ13C values. Also, at line 53, the authors mentioned that lipids are depleted in δ13C and δ2H. If depleted lipids are removed in animal tissues, its tissues will be enriched in δ13C, δ2H, and possibly in δ15N due to solvent that removed protein that are lipophilic. At lines 88-91, the authors mentioned that the δ13C values of larvae lamprey become unexplainably enriched after mathematical corrections, thus lipids seem depleted in δ13C for this specific example and it is consistent with the literature. In the literature, there is a negative relationship between CN and δ13C values, as the CN values increase, the δ13C values in tissues decreases. However, for larvae lampreys, there is a positive relationship between CN and δ13C values (line 72), which is very intriguing. When the CN is high, the δ13C is also high. Did the authors expect a depletion in δ13C values of muscle after lipid extraction due to this relationship? Why did the authors expect a δ13C depletion after lipid extraction? I would suggest clarifying the idea here because it is not intuitive.

We have worked to clarify the logic behind the hypotheses. We summarize our thinking here to clarify as well. If tissues of larval lamprey δ^13^C are corrected with standard equations, they will rise because all current equations anticipate that lipids will have lower δ^13^C values than muscle. This results in values that are so high that the lampreys can only be explained by sources outside the system; even piscivorous fishes in the same stream have lower δ^13^C. Another possibility, the one we support, suggests that something is wrong with the expectations around lipids.

Minor comments
Abstract:
1- Line 20, I would change “source contributions” for “dietary source contributions”.

We have made the change following the reviewer’s comment.
2- Line 22, I would remove “for”.

We retained ‘for’ as its removal causes the sentence to be grammatically incorrect (‘for’ is part of a multi-word verb starting with ‘accounted’).
3- Line 26, I would add a sentence about nitrogen isotope in order to introduce the objective that include the three isotopes.

We have added a clause to the sentence to make it appear more naturally in the abstract.
4- Line 28, I would change “novel” for “different”.

We have made the change following the reviewer’s comment.
5- Lines 31-32, I would change “bulk and lipid-extracted muscles, as well as the extracted lipids” for “bulk and lipid-extracted muscle samples, as well as in the extracted lipid samples”.

We have made the change following the reviewer’s comment.
6- Line 36, I would replace “but” by “and” and what is the meaning of negative? The δ13C of lipids is enriched compared to the δ13C values of muscle? Please clarify and modify the wording.

We have modified the sentence. We moved the word negative closer to the contrast in the statement (le muscle – lipid) to make it clearer.

Introduction:
7- Line 56, I would suggest adding something about nitrogen. This point is not mentioned anywhere in the article.

We have clarified the origin for discrimination during lipid synthesis and added a sentence at the end of the paragraph expressly dealing with nitrogen.
8- Lines 59-60, I would suggest changing “by chemically extracting them from a sample or mathematically correcting for them prior to interpretation” for “by chemically extracting them from a sample before stable isotope analysis or mathematically correcting for them a posteriori using validated normalization model” to clarify.

We have made the change following the reviewer’s comment.
9- Lines 61-62, I would change “the number of samples” for “the number and quantity of samples as lipid extraction can remove lipophilic proteins which could alter nitrogen stable isotope ratios.”.

We have not added ‘and quantity’ because it is not clear how number and quantity are different, but we have added a period a source following the reviewer’s comment.
10- Lines 62-65, please clarify these sentences regarding the samples used for the carbon vs. nitrogen analysis. You may change for “The best practice is to run the lipid-extracted sample for carbon and hydrogen analysis and the bulk sample for nitrogen analysis. If workers choose to account for lipids a posteriori through a mathematically correction, they should use a validated model that typically used a proxy of lipid content and isotopic values of the bulk samples.”

We have modified the sentence following the reviewer’s comment.
11- Lines 66-72, I would suggest adding a few sentences about the relationship (or not) between CN ratio and δ2H, but also %H and δ2H. This will add context to the objectives/hypotheses at lines 107-108.

Relatively little has been done with δ^2^H in larval lampreys, but we cite them here and note the dissertation where bulk samples were measured for δ^2^H and C/N analysis.
12- Line 72, could the lipids of larvae lampreys have a role to play in this relationship? Are there any possible explanations/hypotheses?

We have now explicitly stated that these correlations suggest lipids alter bulk values in stable isotopes.
13- Line 90, please clarify what it is marine predators and why the δ13C signatures of larvae lampreys resemble more to them? Because at line 96, when lipids are extracted (and it correct to do that), they are entirely dependent on allochthonous detritus.

We have deleted this cause because, while true, it is a qualitative observation. The point we were trying to make is that larval lamprey δ^13^C can be the highest value in streams. It is already clear from the introduction that larval lamprey δ^13^C is unusual.
14- At line 106, the authors hypothesized that δ13C would be depleted in muscle after lipid extraction but according to the literature, if deleted lipids in organism are removed, the organism muscles will become enriched. Something is missing to better understand why the authors hypothesized that. Because at lines 88-91, δ13C values of larvae remain unexplainably enriched after mathematically corrections. I would suggest clarifying the idea in the introduction.
We have clarified this is based on lamprey work. We expect that δ^13^C will decline in muscle after lipid extraction based on larval lamprey reports, not on the general trend expected for other species. We do not assume larval lamprey will have the same response as other groups because prior literature has already observed unusual behavior in their isotope ratios.

Methods:
15- Line 142, did the authors measure the % of lipid in the supernatant?

We did not measure the % lipid in the supernatant.
16- Line 169, please confirm that the exact values are 0.2 to 1.2mg? Usually, it is 1.0 to 1.2mg or around 0.500mg. The range is very large.

This is the correct range, generally we tried to pack around 0.5 mg, but for lipids especially we preferred to use smaller quantities.
17- Line 227, do you think it can have a year effect on stable isotope signatures? It could be confused with the month.
We did not measure across multiple years but given larval dependence on benthic sources we would not expect isotopic signatures to vary greatly year to year.

Results:
18- Lines 264-267, I would remove these sentences because it is not a result. I would be more concise in the result section. It is not necessary to repeat the why of the analysis (e.g., lines 265-267, 269-272).

We deleted the rationale for the approach, leaving these in entirely in the methods now.
19- Lines 269-275, results are from linear regression? I would also write the r value in brackets along with the F and p values).

We have included the Pearson’s correlation coefficient (ρ).

Discussion:
20- Lines 456-458, please clarify the link between running analysis in duplicate and the seasonal effect on mathematical correction? It seems 2 ideas from me. It is not the mathematical correction that depend on the season but the difference muscle-lipid that depend on the season? We run analysis in duplicate because δ13C is run on lipid-extracted sample while δ15N is run on bulk sample because lipid extraction affects δ15N values.
We have changed the sentence to explain more clearly what we mean, as written, the reviewer is correct, it sounds as if we are asking people to run four analyses for each sample.

Figure 1. It would be interesting to add boxplot of the lipid isotopic values beside the isotopic values of lamprey muscles (bulk and lipid-extracted) for the three isotopes. It would facilitate the visualization of the isotopic values of lamprey’s lipids.

We considered this and went back and forth on how best to present these data. Ultimately, we left the figure as is for three reasons: 1) the hypothesis being evaluated only considers the difference before and after lipid extraction (i.e., there is no test associated with the lipids to reference when these data would be introduced), 2) the δ^15^N will not have a lipid value, 3) those lipid data are already available in S2 Fig.
Figure 2a. Please write the whole equation, it is truncated.

We have moved the equations to be fully in frame, we apologize that we missed this.

So, thanks for this interesting manuscript that explored the lipid effect on stable isotope signatures in larvae lampreys. It is very intriguing why the lamprey’s lipids are enriched in δ13C instead to be depleted and the positive relationship between CN and δ13C values.

We thank the reviewer for their comments and their work throughout this process. Their efforts have refined and improved the manuscript at all levels.

Reviewer #3: Dear authors,
This interesting piece of work presents a study on the stable isotope ratios of carbon, nitrogen, and hydrogen in the muscle of four lamprey species. The objective of the study was to determine if the stable isotope ratios of these elements behaved as expected, or if the lipids in the muscles presented novel isotopic behavior. I feel the author deserves publication of this work, however there are few queries that need to be addressed first.

Abstract
Overall, the abstract presents a clear and concise summary of the study's results and provides sufficient detail for the reader to understand the methodology and findings.

Introduction
It is well-written and informative. It provides a clear explanation of stable isotope ratios and their importance in studying biological processes. It also addresses the issue of accounting for lipids in stable isotope analysis, which is an important consideration that researchers need to take into account when interpreting results. The mention of the specific example of larval lampreys and the discrepancies in their stable isotope ratios adds an interesting layer to the discussion. However, I feel that few more recent works are available which can be incorporated.

We reconsidered every reference in the introduction but did not find any that we would want to replace. The work we are referencing is specific to larval lamprey isotopes, so while more work has been done on isotopes in lampreys, we have not included all isotopes studies with lampreys. If the reviewer feels we have missed a critical study, we would be happy to add it.

Discussion
Perfectly fine, and issues detected.
My general view is the paper can be accepted with slight changes as suggested.

We thank the reviewer for their time and effort.

7. PLOS authors have the option to publish the peer review history of their article ([what does this mean?](https://journals.plos.org/plosone/s/editorial-and-peer-review-process#loc-peer-review-history)). If published, this will include your full peer review and any attached files.


If you choose “no”, your identity will remain anonymous but your review may still be made public.


**Do you want your identity to be public for this peer review?** For information about this choice, including consent withdrawal, please see our [Privacy Policy](https://www.plos.org/privacy-policy).

Reviewer #2: No

Reviewer #3: No
